# Evolving Trends and Burden of Inflammatory Bowel Disease in Asia, 1990–2019: A Comprehensive Analysis Based on the Global Burden of Disease Study

**DOI:** 10.1007/s44197-023-00145-w

**Published:** 2023-09-01

**Authors:** Xuejie Chen, Xin Xiang, Weitong Xia, Xindi Li, Sidan Wang, Shuyu Ye, Li Tian, Lian Zhao, Feiyan Ai, Zhaohua Shen, Kai Nie, Minzi Deng, Xiaoyan Wang

**Affiliations:** Department of Gastroenterology, The Third Xiangya Hospital, Central South University, 138 Tongzipo Road, Changsha, 410013 Hunan People’s Republic of China

**Keywords:** Global Burden of Disease, Inflammatory bowel disease, Comprehensive analysis

## Abstract

**Background:**

Asia’s inflammatory bowel disease (IBD) burden has rapidly increased recently, but the epidemiological trends in Asia remain unclear. We report IBD’s incidence, prevalence, mortality, and Disability-Adjusted Life Years (DALY) in 52 Asian countries from 1990 to 2019.

**Methods:**

Data from the Global Burden of Disease 2019 were analyzed for IBD burden across 52 countries, using metrics like incidence, prevalence, mortality rates, and DALY. The epidemiological trend of IBD from 1990 to 2019 was assessed with the Joinpoint and APC methods. Decomposition and frontier analyses examined factors behind IBD case and death changes. The NORPRED forecasted Asia's morbidity and mortality trends from 2019 to 2044.

**Results:**

From 1990 to 2019, The incidence and prevalence of IBD increased in Asia, while mortality and DALY decreased. East Asia had the highest increase in disease burden. IBD incidence was highest among the 30–34 age group, with prevalence peaking in the 45–49 age group. In high-income regions, IBD peak age shifted to younger groups. Decompose analysis showed population growth as the primary factor for the increasing IBD cases in Asia. NORDPRED model predicted a continued IBD burden increase in Asia over the next 25 years.

**Conclusions:**

Between 1990 and 2019, ASIR and ASPR of IBD in Asia increased, while ASMR and ASDR decreased. Due to population growth and aging, the IBD burden is expected to rise over the next 25 years, particularly in East Asia.

**Supplementary Information:**

The online version contains supplementary material available at 10.1007/s44197-023-00145-w.

## Introduction

Characterized by chronic, systemic inflammation of the gastrointestinal tract, inflammatory bowel disease (IBD) encompasses both ulcerative colitis (UC) and Crohn's disease (CD). UC lesions are limited to the colon, whereas CD can affect any portion of the gastrointestinal tract, exhibiting non-continuous inflammation. IBD presents with diverse clinical symptoms, including diarrhea, abdominal discomfort, hemorrhage, anemia, and body weight loss. IBD patients typically experience persistent inflammation with limited prospects for complete resolution, which elevates the risk of developing digestive and other malignancies [1, 2].

The incidence and prevalence of IBD in the Western world have remained high in the 21st Century, although they have plateaued in recent years [3]. Simultaneously, with the advancement of industrialization, newly industrialized Asian nations have witnessed a considerable rise in both the incidence and prevalence of IBD [4]. Most research on the IBD disease burden originates from Western countries [5–7], while comprehensive studies analyzing the IBD burden in Asia are still being determined.

Moreover, specific data on the IBD disease burden in Asia contradicts the findings on global trends. For instance, Scholars have documented a higher burden of IBD in Chinese males, contrary to previous studies indicating a more significant worldwide burden of IBD in females [8, 9]. Although some publications examine fundamental indicators, such as the incidence and prevalence of IBD in Asia [10, 11], no published articles have specifically addressed the overall trends of disease burden, future trend predictions, or the contributions of biological and social factors to IBD trends in Asia.

In summary, the IBD burden in Asia is rapidly increasing and exhibits regional uniqueness. However, research on the IBD burden in Asia remains limited and warrants more comprehensive statistical approaches. Given the inherent challenges associated with data collection for the Global Burden of Disease Study 2017 (GBD 2017) database, certain regions, including but not limited to India and China, exhibited discrepancies with the established epidemiological trends for IBD. These anomalies were subsequently addressed and rectified to a significant degree in the Global Burden of Disease Study 2019 (GBD 2019).

## Method

### Data Source

The GBD study provides estimations of incidence, prevalence, sources of morbidity and mortality, and health deterioration across age, gender, year, location, income category, and time for a wide array of diseases, injuries, and crucial risk factors [12–14]. IBD, primarily CD and UC, is identified via endoscopy, imaging examinations, or biopsy in individuals exhibiting pertinent clinical manifestations and indicators. The International Classification of Diseases version 10 (ICD-10) assigns the code K50 to CD, K51 to ulcerative colitis, and K52 to indeterminate colitis [9, 15].

We utilized data from the GBD study 2019 (https://vizhub.healthdata.org/gbd-results/) and extracted annual values and rates of incidence, prevalence, mortality, and DALYs for IBD in Asia from 1990 to 2019, segregated by sex and age. Subsequently, the data were arranged into successive 5-year age brackets, starting from 0 to 4 years and extending to 95 years and beyond.

### Asian Regions

We analyzed the IBD burden across four Asian regions: East Asia, South Asia, Southeast Asia, and Central Asia. Except for four regions in Asia, we also analyzed the burden of IBD in high-income regions of Asia [16]. East Asia encompasses China, Japan, the Republic of Korea, Taiwan (Province of China), and the Democratic People’s Republic of Korea. South Asia includes Bangladesh, Bhutan, India, Nepal, and Pakistan. Southeast Asia comprises Brunei Darussalam, Cambodia, Indonesia, Lao People’s Democratic Republic, Malaysia, Maldives, Mauritius, Myanmar, the Philippines, Seychelles, Singapore, Sri Lanka, Thailand, Timor-Leste, Vietnam, American Samoa, Cook Islands, Fiji, Guam, Kiribati, Marshall Islands, Micronesia (Federated States of), Nauru, Niue, Northern Mariana Islands, Palau, Papua New Guinea, Samoa, Solomon Islands, Tokelau, Tonga, Tuvalu, and Vanuatu. Central Asia consists of Armenia, Azerbaijan, Georgia, Kazakhstan, Kyrgyzstan, Mongolia, Tajikistan, Turkmenistan, and Uzbekistan. High-income Asia Pacific countries are Brunei Darussalam, Japan, the Republic of Korea, and Singapore.

### Estimated Annual Percentage Change

The Estimated Annual Percentage Change (EAPC) is a commonly utilized approach for quantifying trends during a defined time frame [17, 18]. This study utilized the age-standardized Incidence rates (ASIR) for 5-year age groups to calculate the EAPC for Asian countries and regions [19].

### Joinpoint Poisson Regression

The joinpoint regression model comprises a set of linear statistical models employed to assess the trends in disease burdens attributable to IBD over time [8, 20–22]. We assessed the Average Annual Percent Changes (AAPCs) along with their respective 95% CIs to ascertain the extent of temporal trends regarding incidence, prevalence, and mortality rates by employing joinpoint regression analysis [20]. The AAPC was computed as a geometrically weighted mean of diverse annual percentage change values obtained from the regression assessment [23]. The analysis was performed utilizing the ‘Joinpoint’ software supplied by the Surveillance Research Program of the US National Cancer Institute [22].

### Age-Period-Cohort Modeling Analysis

Our study utilized the Age-Period-Cohort (APC) model to examine prevalence trends by age, period, and birth cohort within various Asian regions from 1990 to 2019. The APC model is executed using accessible R tools, with methodological specifics outlined in previous scholarly works [24–26]. The output of the APC model includes the net drift of prevalence and the local drift of age-specific prevalence, representing the average of various annual percentage and birth cohort effect trends, respectively. Additionally, the output includes adjusted longitudinal age-specific rates for the reference cohort, accounting for period deviations and representing age effects. Period and cohort effects are expressed as the relative prevalence risk for each period and cohort. The referent period and cohort choice are arbitrary and do not impact result interpretation.

### Decomposition

Decomposition analysis determines the additive contribution of factor differences in two populations to the disparity in their overall values [27]. By breaking down IBD incidence and mortality according to age structure, population expansion, and epidemiological shifts, it is possible to quantify each element's contribution to the cumulative impact.

### Frontier Analysis

The Frontier analysis determines the minimal attainable IBD burden based on the development status, as indicated by the Sociodemographic Index (SDI) [27, 28]. The frontier delineates nations or regions demonstrating exemplary performance (advancing the boundaries) by having the lowest IBD burden relative to their SDI. The distance from the frontier, referred to as the “effective difference, ” signifies the disparity between the observed burden and the potentially attainable disease burden for a specific country or region, considering their SDI. This gap may be minimized or eradicated based on the sociodemographic resources of the respective country or territory.

### NORDPRED

In order to predict the new cases and incidence rates from 2019 to 2044 across regions, gender, and age, a log-linear age-period-cohort model was employed based on recent trends [29, 30]. For the three or four most recent 5-year observed periods, a power function was utilized to extrapolate and modulate growth, projecting the recent linear trend for the past decade to be diminished by 25%, 50%, and 75% in the second, third, and fourth forecast periods, respectively. The number of new cases for 2044 was estimated by taking a weighted average of the projected incidence rates for the final two prediction periods and applying the rates to the U.N. national population forecasts accessible for each nation in that year [31]. Functions were available in R Studio and the NORDPRED Package [32] (http://www.kreftregisteret.no/en/Research/Projects/Nordpred/Nordpredsoftware/). Statistical tests were two-sided, and *P* < 0.05 was considered significant. All analyses were conducted in R (version 4.2.1).

### Patient and Public Involvement Statement

Patients and the public were not involved in this study.

## Result

### Overall IBD Burden and Time Trends in Asia

**Fig. 1:**
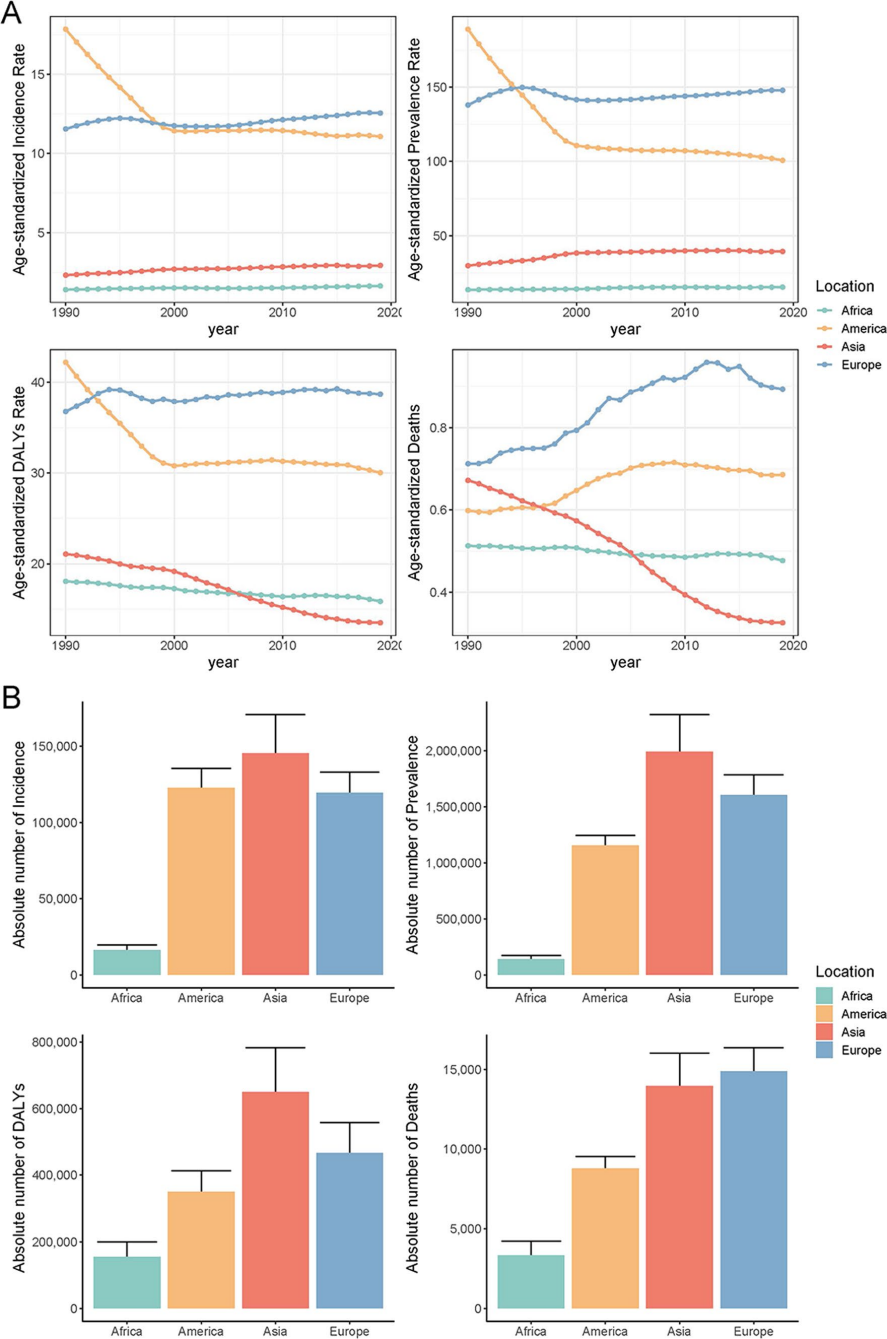
Age-specific numbers and age-standardized prevalence, incidence, and mortality rates of IBD in four continents. **A** Trends of ASIR, ASPR, ASDR, and ASMR of IBD in Asia, America, Africa, and Europe from 1990 to 2019. **B** Absolute incidence number, prevalence number, DALY number, and deaths number of IBD in Asia, America, Africa, and Europe in 2019. IBD, Inflammatory bowel disease; DALY, disability-adjusted life year; ASIR, Age-standardized incidence rates; ASPR, Age-standardized prevalence rates; ASDR, Age-standardized DALY rates; ASMR, Age-standardized mortality rates

During 1990 and 2019, the cases of IBD in Asia increased from 763,544 (95% UI: 634,873.9, 905,118.8) to 1,992,221.5 (95% UI: 1,707,091.6, 2,322,097.7). In 2019, the absolute incidence and prevalence of IBD in Asia were the highest among the four continents (Fig. 1).

**Fig. 2:**
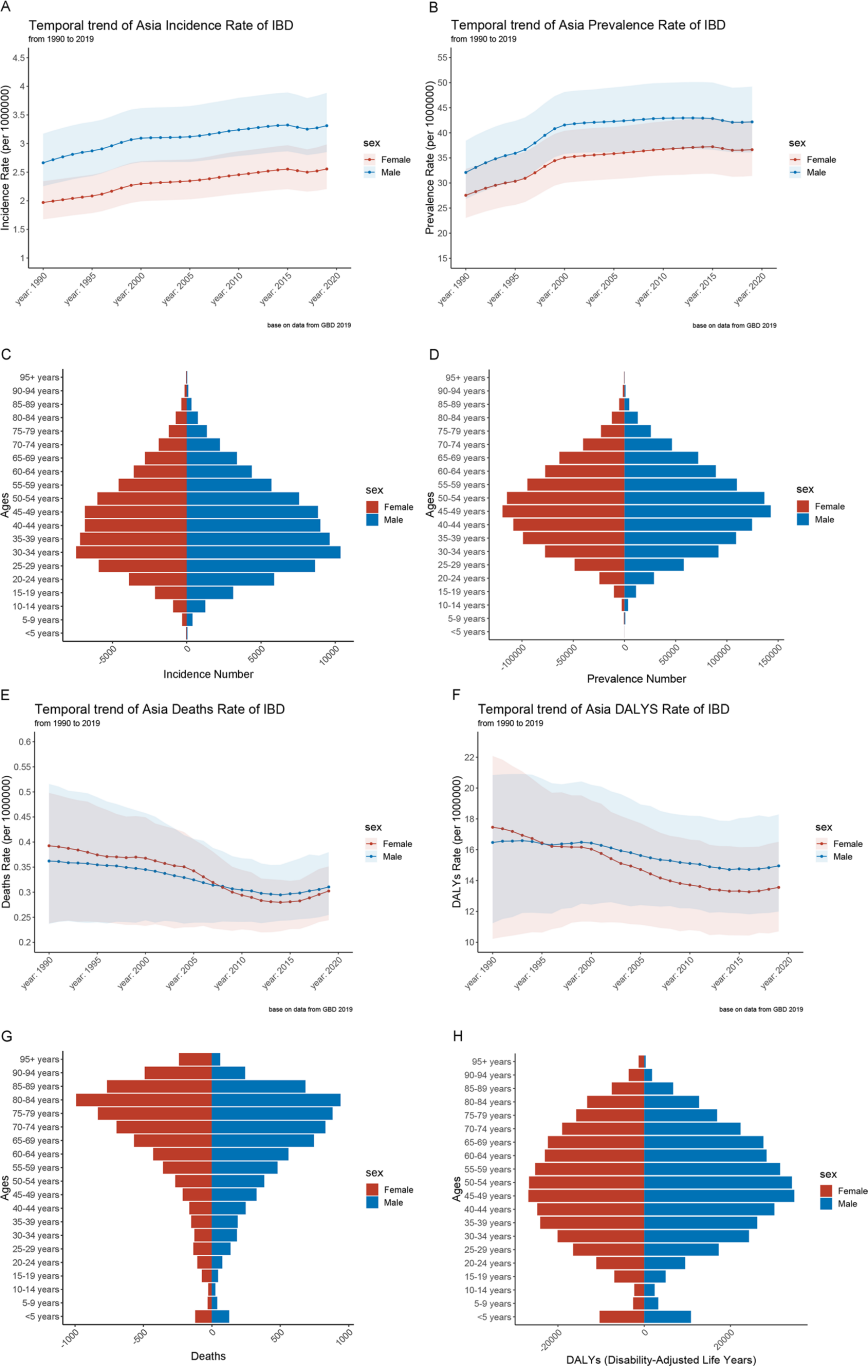
Age-specific numbers and age-standardized incidence, prevalence, mortality and DALY rates of IBD in Asia. **A** Trends from 1990 to 2019 in ASIR of IBD in Asia; **B** Trends from 1990 to 2019 in ASPR of IBD in Asia; **C** Asia cases of incidence across age groups, 2019; **D** Asia cases of prevalence across age groups, 2019; **E** trends from 1990 to 2019 in ASMR of IBD in Asia; **F** trends from 1990 to 2019 in ASDR of IBD in Asia; **G** Asia cases of deaths across age groups, 2019; **H** Asia number of DALY across age groups, 2019. IBD, Inflammatory bowel disease; ASIR, Age-standardized incidence rates. ASPR, Age-standardized prevalence rates; ASMR, Age-standardized mortality rates. DALY, disability-adjusted life year. ASDR: age-standardized DALY rate

From 1990 to 2019, although the age-standardized incidence rates (ASIR) and age-standardized prevalence rates (ASPR) of IBD in Asia were lower than those in America and Europe, Asia experienced a substantial rise in the ASPR of IBD, from 29.81 (95% UI: 24.93, 35.53) per 100,000 population in 1990 to 39.37 (95% UI: 33.70, 45.81) per 100,000 population in 2019. Both incidence and prevalence were higher in males than females during the study period. In 2019, overall male prevalence cases were 1,068,534.5 (95% UI: 913,133.7, 1,246,869.8) (53.64%), while female cases were 923,687.0 (95% UI: 793,213.7, 1,072,032.4) (46.36%). The 2019 ASPR was 42.18 (95% UI: 36.08, 49.20) per 100,000 population for males and 36.64 (95% UI: 31.42, 42.45) per 100,000 population for females. Gender had no impact on the age distribution of IBD, as peak incidence was observed in both males and females within 30–34 years, and peak prevalence occurred at 45–49 years (Fig. 2A–D, Appendix table 1, Appendix table 2).

From 1990 to 2019, the cases of IBD-related deaths increased from 12,029.6 (95% UI: 8,669.2, 15,526.3) to 13,957.4 (95% UI: 11,897.9, 16,020.8). Despite the rise in death count, Asia’s age-standardized mortality rates (ASMR) declined from 0.67 (95% UI: 0.46, 0.90) per 100,000 population in 1990 to 0.33 (95% UI: 0.28, 0.37) per 100,000 population in 2019. Between 1990 and 2019, female mortality decreased faster than male mortality. In 2019, most deaths occurred among elderly individuals, with peak deaths observed between ages 80 and 84 for both males and females (Fig. 2E, G, Appendix table 3).

Between 1990 and 2019, the total number of disability-adjusted life years (DALYs) attributed to IBD in Asia increased from 540,948.6 (95% UI: 382,320.4, 666,324.4) to 649,760.1 (95% UI: 530,394.6, 783,180.6). However, the age-standardized DALY rates (ASDR) decreased from 21.08 (95% UI: 15.82, 25.98) per 100,000 population in 1990 to 13.51 (95% UI: 11.08, 16.21) per 100,000 population in 2019. In 2019, the highest number of DALY was observed in the 45–49 age group, with a subsequent decline in older age groups. From 1990 to 2019, the trend in ASDR rates was similar to that of ASMR, with females exhibiting a more significant decline than males (Fig. 2F, H, Appendix table 4).

The burden of the disease varies among Asian regions. Between 1990 and 2019, ASIR and ASPR increased in East, Central, South, and Southeast Asia. East Asia exhibited the highest increase in incidence rates (AAPC =  + 2.5, 95% CI: 2.4, 2.6). Southeast Asia maintained the lowest incidence and prevalence rates among the four regions.

Age-standardized mortality rates (ASMR) declined in all four Asian regions from 1990 to 2019, with East Asia exhibiting the steepest decline (AAPC = − 3.5, 95% CI: − 3.3, − 3.7). However, East Asia still had the highest number of deaths, decreasing from 5734.25 (95% UI: 4169.43, 7373.88) in 1990 to 4982.78 (95% UI: 4074.27, 5769.10) in 2019. In comparison, Central Asia had the lowest number of deaths in 2019, at 249.12 (95% UI: 218.34, 287.60) (Appendix figure 1, Appendix figure 2).

**Fig. 3:**
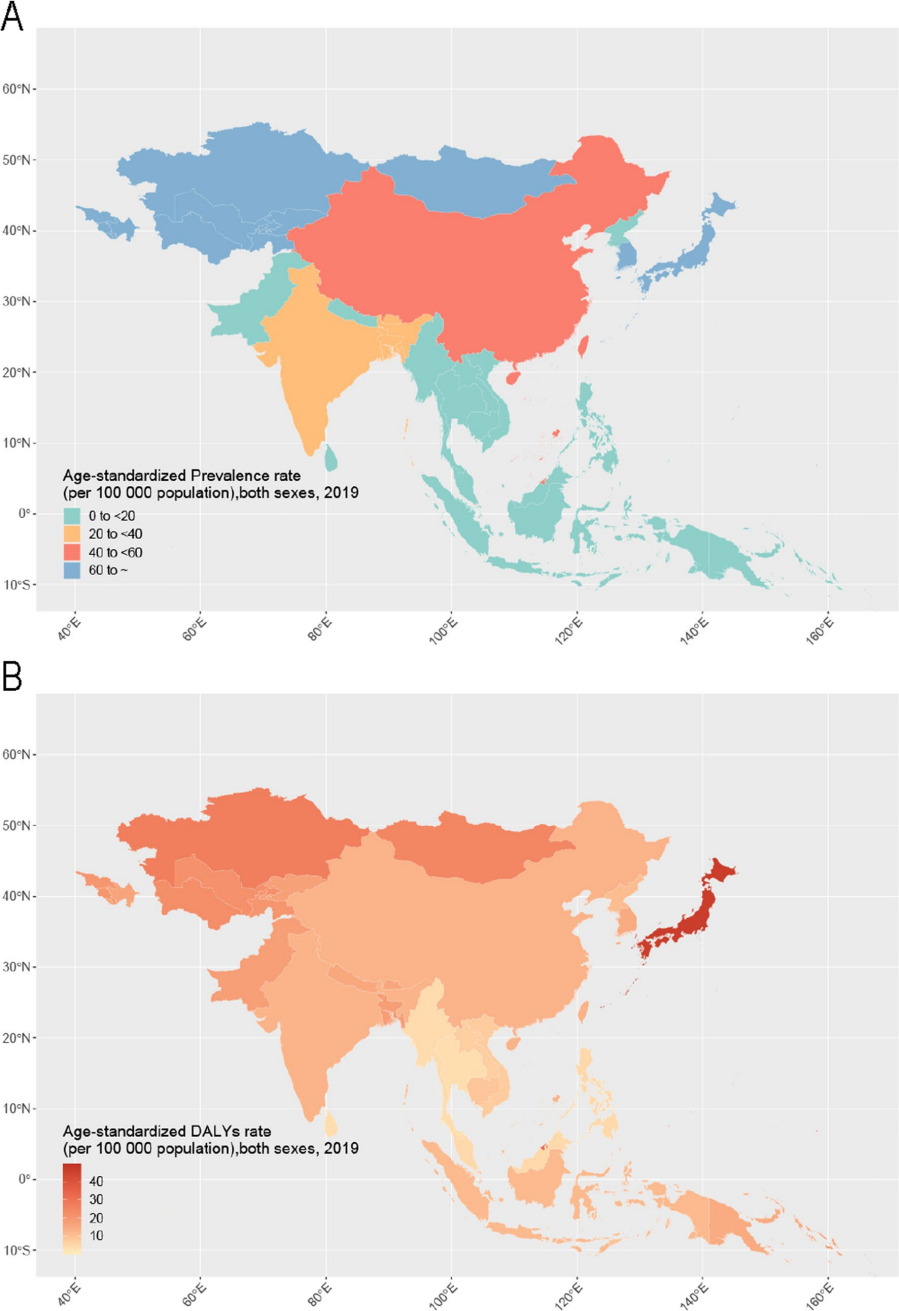
Maps of age-standardized prevalence and DALY rates of IBD in Asian countries and regions. **A** ASPR, **B** DALY of IBD burden in Asian countries and regions. IBD, Inflammatory bowel disease; ASPR, Age-standardized prevalence rates; DALY, disability-adjusted life year; ASDR, Age-standardized DALY rates

Figure 3 presents the Asian country-level ASPR and ASDR maps for IBD in 2019. Japan had the highest ASIR (19.65 per 100,000) and ASPR (291.90 per 100,000), far higher than the country in second place. The country had second ASIR was Turkmenistan (7.69 per 100,000), the country had second ASPR was Georgia (87.97 per 100,000) the country had the second ASDR was Brunei (32.76 per 100,000). Thailand had the lowest ASIR (0.460 per 100,000), while the Solomon Islands had the lowest ASPR (3.35 per 100,000) (Fig. 3A, Appendix figure 3, Appendix table1, Appendix table2). Thailand (3.64 per 100,000) and Singapore (0.073 per 100,000) had the lowest DALY and mortality rates, respectively (Fig. 3B, Appendix figure 4, Appendix table 3, Appendix table 4).

Throughout the study, the burden of IBD demonstrated varying increase rates across numerous Asian countries and regions. The ASIR exhibited the most rapid growth in the Taiwan region of China, with an annual average increase of 3.87% (95% CI: 3.48, 4.27%), followed by Vietnam at 2.62% (95% CI: 2.13, 3.11%) and mainland China at 2.54% (95% CI: 2.4, 2.68%). In contrast, Singapore (EAPC = − 0.35, 95% CI: − 0.56, − 0.14) and Georgia (EAPC = − 0.16, 95% CI: − 0.21, − 0.12) experienced a decline in ASIR, as illustrated in Appendix figure 5.

### Trends in Incidence, Prevalence, and Mortality Rates Across Different Age Groups Based on Joinpoint Regression Analysis

We conducted the joinpoint regression analyses of ASIR, ASPR, and ASMR for IBD in Asia from 1990 to 2019 to describe the burden of IBD disease in Asia. The incidence increased among females from 1990 to 1996 (APC =  + 1.2 (95% CI: 1.1, 1.2)), and the trend is more evident between 1996 and 1999 (APC =  + 2.6 (95% CI: 2.3, 2.8)). In contrast, the incidence trend for males differed slightly, increasing from 1992 to 2000 (APC =  + 1.5 (95% CI: 1.4, 1.5)). Notably, the increasing trend of incidence rates slowed down after 2000 (APC =  + 0.1 (95% CI: − 0.2, 0.4)) from 2000 to 2005) (Appendix figure 6–14).

The trend of ASPR mirrored that of the incidence rates. The prevalence rates increased from 1990 to 2019 (AAPC =  + 0.9 (95% CI: 0.8, 1.1)). Meanwhile, ASMR in Asia exhibited a significant downward trend during the study period (AAPC = − 2.5 (95% CI: − 2.5, − 2.4); however, the decline slowed after 2016. Between 1997 and 2013, ASMR decreased significantly for both males (APC = − 2.1 from 1997 to 2002 and APC = − 3.3 from 2002 to 2013) and females (APC = − 2.5 from 2000 to 2004, APC = − 5.3 from 2004 to 2010, and APC = − 4.0 from 2010 to 2013). Interestingly, males had lower AAPCs for incidence, prevalence, and mortality rates than females, as shown in Appendix table 5.

### Age, Period, and Cohort Effects on IBD Prevalence

**Fig. 4:**
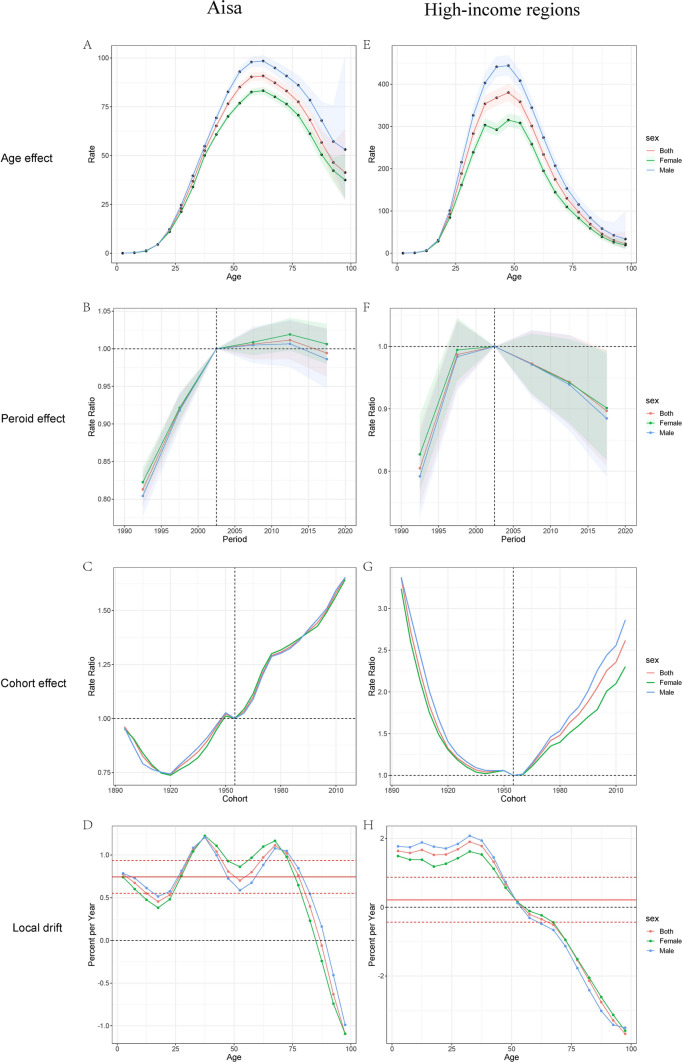
Age-period-cohort effects of prevalence from 1990 to 2019 in Asia and High-income regions in Asia. **A** Age effects are rep-resented by the fitted longitudinal age curves of prevalence (per 100,000 person-years) adjusted for period deviations. **B** Period effects are represented by the relative risk of prevalence (prevalence rate ratio) and computed as the ratio of age-specific rates in each period compared to the referent 2000–2004 period. **C** Cohort effects are represented by the relative risk of prevalence (prevalence rate ratio) and computed as the ratio of age-specific rates in each cohort compared to referent 1955 cohort. **D** Local drifts indicate the annual percentage change of prevalence (% per year) across five-year age groups (from 0 to 4 to 95 plus years). The shaded areas indicate the corresponding 95% CIs of each point estimate

Age, period, and cohort effects across multiple locations were estimated using the Age, period, and cohort model, and the corresponding results are depicted in Fig. 4. We observed similar age-effect relationships across different regions, the 60–64 age group was associated with the highest risk. The risk increased with age up to 60 years and subsequently decreased after the age of 66 years. Notably, in the high-income Asia region, IBD peaked at younger ages, specifically in the 45–49 age group, compared to other Asian regions (Appendix table 6).

The period effects revealed an increased risk of IBD prevalence across various regions from 1990 to 2019. In Asia, females exhibited a higher risk of IBD due to the period effect. The period effect revealed that between 1900 and 1955 Asia experienced a significant rise in the incidence of IBD. However, from 1955 to 2019, the period effect had no considerable impact on the risk of IBD (Appendix table 7).

Age-specific trends for Asia were summarized by birth cohort using relative risks, with the 1955 birth cohort as the reference group. In Asia, cohort relative risks increased from 0.96 (95% CI: 0.2, 4.66) in 1895 to 1.0 in 1955, followed by an increase to 1.65 (95% CI: 0.21, 13.16) in 2015. Moreover, the cohort relative risks of the high-income Asia region decreased from 3.36 (95% CI: 0.16, 69.51) in 1895 to 1.0 in 1955 and then increased to 2.62 (95% CI: 0.001, 43,266.86) (Appendix table 8).

In Asia, the prevalence of IBD exhibited an increasing trend among individuals younger than 85 years and a decreasing trend among those older than 85. The most substantial prevalence increase occurred in the 35–39 age group (1.21% per year, 95% CI: 1.10, 1.32), while the 60–74 age group also experienced a significant increase in prevalence rates (1.11% per year, 95% CI: 0.93, 1.29). Moreover, the highest increase in high-income regions occurred in the 30–34 age group (1.90% per year, 95% CI: 1.61, 2.19). The trends differed between males and females, with females in the 35–69 age group experiencing a more significant rise in prevalence than other age groups (Appendix table 9).

### Factors Influencing IBD Epidemiology Include Population Growth, Aging, and Changes in Epidemiological Patterns

**Fig. 5:**
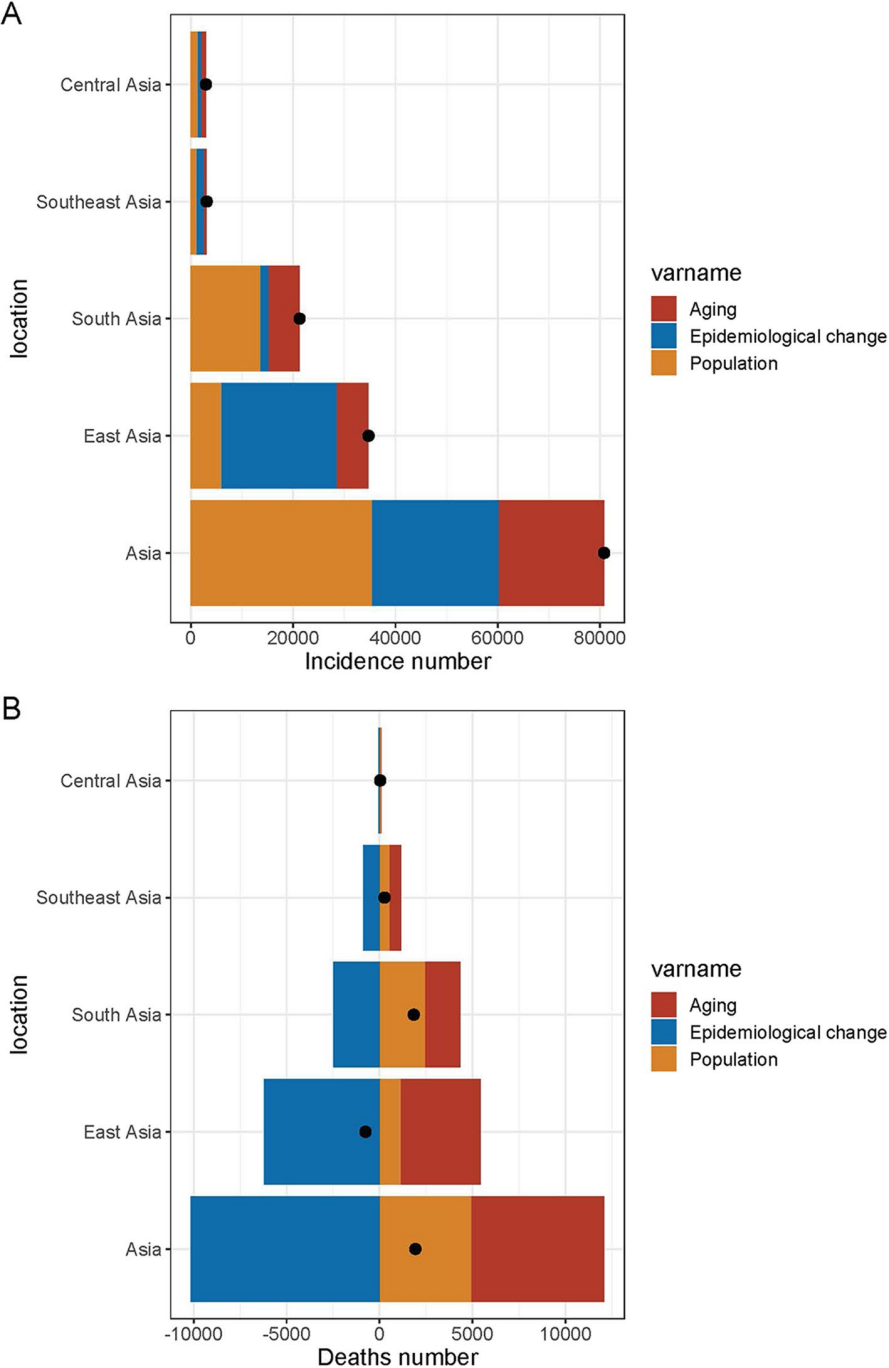
Changes in IBD **A** incidence, **B** deaths according to population-level determinants of population growth, aging, and epidemiological change from 1990 to 2019 across location. The black dot represents the overall value of change contributed by all three components. For each component, the magnitude of a positive value indicates a corresponding increase attributed to the component; the magnitude of a negative value indicates a corresponding decrease attributed to the related component

In order to evaluate the impact of aging, population growth, and epidemiological changes on IBD epidemiology over the past 30 years, we conducted a decomposition analysis of incidence and mortality rates (Fig. 5). The incidence of IBD has significantly risen in Asia, with the most significant increases observed in South and East Asia. In Asia, population growth and aging contributed to 43.94% and 25.56% of the increase in IBD morbidity burden from 1990 to 2019, respectively. Aging made the most significant contribution to the incidence of IBD in South Asia (28.46%), followed by Central Asia (28.16%), Southeast Asia (18.83%), and East Asia (17.88%). Population growth was the primary driver of the increase in IBD incidence (64.27% in South Asia, 49.1% in Central Asia, and 37.7% in Southeast Asia). In East Asia, population growth had a relatively minor contribution to the increase in IBD incidence (17.41%) (Fig. 5A, Appendix table 10).

Over the past 30 years, epidemiological changes reflecting potential shifts in age and demographically adjusted IBD incidence have increased in Asia, with a minor substantial increase in South Asia (7.26%) and more considerable increases in East Asia (64.71%) and Southeast Asia (43.47%). Aging and population growth have been the primary drivers of changes in IBD incidence in Asia throughout the study period.

IBD-related deaths have increased in all Asian regions except East Asia. Mortality rates have been declining across all regions, with the most rapid decline occurring in East Asia (-824.98%), which accounts for the most significant reduction in deaths. Despite the downward trend in mortality rates, the number of deaths has risen in Asia due to aging and population growth (Fig. 5B, Appendix table 11).

### The Association Between Sociodemographic Development and IBD Burden in Asia

We analyzed age-standardized DALY rates (ASDR) weighted by population, stratified by the World Bank income classification [27], revealing that high-income regions in Asia experienced a significantly more significant burden of IBD. Nevertheless, as a country develops, the DALY rates decrease. To better understand the potential improvements achievable in IBD ASDR, we conducted a frontier analysis based on ASDR and SDI for each Asian country (Appendix figure 19).

The frontier line distinguishes countries with the lowest DALY rates relative to the SDI. The “effective difference” reflects the gap between a country's observed and potentially achievable DALYs, which could be reduced or eliminated with additional sociodemographic resources. We calculated the practical difference from the frontier for each country and territory using 2019 DALY and SDI data. The top 15 countries with the highest adequate difference (range: 43.03–16.79) included Japan, Brunei, Kiribati, Kazakhstan, Nauru, Cook Islands, Marshall Islands, Vanuatu, Mongolia, Micronesia, Turkmenistan, Uzbekistan, Tonga, Armenia, and Georgia. Countries with similar sociodemographic resources exhibited lower IBD DALY rates than those observed in the countries of interest. The five countries with the lowest DALY rates and minor effective differences were among those with an SDI of less than 0.5 (range: 2.10–9.73), including Laos, Cambodia, Papua New Guinea, Nepal, and Bhutan. In the four regions with an SDI above 0.85, namely Japan, South Korea, Taiwan, and Singapore, their distance difference ranges from 43.03 to 4.72. Furthermore, we observed that the distance difference for Japan increased year by year, while the distance difference in most other countries displayed a downward trend (Appendix table 12).

### Changes in Incidence and Mortality in Asia Over the Next 25 Years Across Five Regions, Based on Nordpred

We observed an increasing incidence in all regions over the next 25 years, except for South Asia (ASIR: 2.3 in 2019 vs. 1.79 in 2044). East Asia was projected to remain the region with the fastest-growing ASIR (2.95 in 2019 vs. 3.43 in 2044). Central Asia (ASIR: 6.89 in 2019 vs. 6.91 in 2044) and Southeast Asia (ASIR: 0.7 in 2019 vs. 0.78 in 2044) were expected to maintain the highest and lowest ASIR in Asia, respectively. The ASIR in Asia (ASIR: 2.94 in 2019 vs. 2.9 in 2044) and Southeast Asia (ASIR: 0.70 in 2019 vs. 0.78 in 2044) have remained stable from 2019 to 2044. The ASIR for males was higher than for females in all regions from 2019 to 2044. Although the incidence rates were declining in South Asia and Asia, the absolute cases were expected to continue increasing due to population growth and other factors (Appendix figure 20A). Additionally, we found that the number of cases in high-income regions of Asia was projected to decrease from 27,083.76 in 2019 to 23,810.38 in 2044, despite the rising incidence rates (ASIR:14.98 in 2019 vs. 16.07 in 2044). The incidence number decline in High-income regions of Asia may be attributed to changes in the region’s population. Mortality rates were anticipated to continue decreasing in all regions over the next 25 years (ASMR: 0.33 in 2019 vs. 0.3 in 2044 for Asia) (Appendix figure 20B, Appendix table 13).

## Discussion

Our study provides the first comprehensive analysis of the burden of IBD in Asia, synthesizing data from the Global Burden of Disease Study 2019. This assessment encompasses overall and age-specific incidence, prevalence, mortality, and DALYs of IBD, as well as their long-term trends and variations across gender and distinct Asian regions. We found that nearly one million males and almost 900,000 females in Asia lived with IBD in 2019, and the prevalent cases are increasing. Our study supports Prior research that has reported that high-income North America and Europe have significantly contributed to the global number of IBD patients [33–35]. Although the ASIR and ASPR of IBD in Asia were lower than in America and Europe, Asia had the highest absolute incidence and prevalence of IBD among the four continents in 2019 due to its large population.

Regionally, Central Asia had the highest ASIR in Asia and remained relatively stable without significant increases during the study period [36–38]. East Asia has the fastest-growing ASIR, mainly in high-income countries. Previous studies have suggested that some common risk factors, including genetic susceptibility, smoking, diet in high-income countries, contribute to the high incidence of IBD [9, 39–41].

Furthermore, recent studies have shown that the risk factors of IBD in Asia are similar to those in European and American countries but also have some differences. Urbanization, industrialization, and other environmental factors are risk factors for IBD in Asia and have been associated with an increased incidence of the disease [42]. In Asia, urbanization and industrialization have rapidly expanded over the past few decades, leading to significant lifestyle and dietary changes. Many Asian countries have observed a shift toward a Westernized diet characterized by high consumption of red meat, processed foods, and refined sugars [43, 44]. This dietary change may contribute to the increased prevalence of IBD. Smoking is another known risk factor for IBD. While smoking rates have decreased in high-income countries, they remain high in some low and middle-income Asian countries [45, 46].

Moreover, the lowest prevalence is found in South and Southeast Asia which may could be attributed to the methodological approach employed in non-lethal models. In these models, where data shortages are particularly significant, the estimates for some regions are derived exclusively from a synthesis of these contributing factors, along with data procured from individual countries, including those in South and South-East Asia [9, 47]. Due to challenges in diagnosing IBD and the lower level of development in South and Southeast Asia compared to other regions, the lack of relevant medical experts and facilities makes IBD diagnosis even more complex [11, 37].

Over the past 30 years, the ASMR and ASDR of IBD in Asia have demonstrated a downward trend, which may indicate improved survival for patients with IBD in Asia. This improvement could be attributed to advancements in medical standards across Asian countries and regions, increased IBD specialists, early detection of IBD, and the wide use of biologics. As the lower prevalence of IBD in countries with a lower SDI, we observed a lower ASMR in these countries compared to those with a higher SDI. However, the low cases of reported deaths in countries with a low SDI may be due to the poor quality of death registries in these areas.

Our findings indicate that the number of IBD cases has increased substantially in Asia, especially in East Asia, with population growth and epidemiological changes serving as the primary drivers of this trend. We also conducted a decomposition analysis of mortality in Asia. Although the mortality rates were declining in Asia, the number of deaths increased due to aging and population growth. Only in East Asia, where healthcare has developed more rapidly, the effect of decreasing mortality rates surpassed that of aging and population growth, resulting in decreased deaths during the study period.

We draw several conclusions from the decomposition analysis. Firstly, the demographic expansion, including population growth and aging, has significantly contributed to the change in the burden of IBD in each Asian region. Secondly, although mortality rates due to IBD have decreased in each region, further measures are necessary to offset the impact of population growth and aging. Third, the increasing incidence, aging, and population growth have collectively contributed to a rise in annual cases in each Asian region. Considering the inevitability of the aging process and its significant impact on the rise in IBD cases, it's essential to strategize how to address this challenge. Preventive measures, early diagnosis, and community awareness can be crucial in mitigating the effects of aging on IBD incidence. By focusing on primary prevention, such as promoting healthy lifestyle habits and educating populations on potential risk factors, the impact of an aging population on the burden of IBD might be offset to some extent. Fourth, the burden of IBD is more heavily skewed towards developed, fast-growing economies.

The age distribution of IBD was not affected by gender, with peak incidence observed at 30–34 years and peak prevalence at 60–64 years for both males and females. Our study observed a single peak in the incidence of IBD, contrasting with the previously proposed bimodal pattern in prior studies. Additionally, we report a shift of IBD prevalence toward younger ages in high-income regions of Asia, with a peak age of 25–29, which may be due to earlier onset in high-income areas and the rapid increase in incidence and earlier detection of IBD in High-income Asia regions [48, 49].

The number of IBD patients in Asia is continuously mounting, yet the medical resources dedicated to IBD must be increased to address patients’ needs. Currently, three-quarters of the global IBD population reside in developing countries [50], with 2.7 billion living in China and India, which may bring a substantial economic and healthcare burden in Asia [4]. This surge not only places pressure on medical infrastructures but also translates to escalating economic burdens. Key contributors to this financial load include costs associated with treatment, long-term care, lost labor hours, and decreased productivity [51]. Furthermore, expenses linked to the management of IBD, such as medications, hospitalizations, surgeries, and routine monitoring, can be substantial. Especially for low- and middle-income families, these costs can be prohibitive [51]. With advancements in IBD therapeutics, there's an emerging trend towards the earlier introduction of biologics and small-molecule drugs. Such treatments, like Adalimumab, necessitate prolonged use, further exacerbating the economic strain. A study from Iran illustrated that the annual medical expenses for IBD patients reached up to $1229.74, while the global expenditure for Adalimumab alone touched $200 billion in 2019 [52]. Research from the U.S. indicated that the odds of IBD patients encountering financial hardships were 2.49 times higher than those without the condition [53]. In such contexts, there’s a pronounced inclination among patients to seek cost-reduction strategies, express preference for cheaper medications, or resort to alternative therapies. These choices, unfortunately, might compromise therapeutic outcomes, potentially leading to an increase in DALYs due to suboptimal disease management. Options to mitigate the financial burden on IBD patients are currently limited. Some studies suggest that a viable alternative could be the broader adoption of biosimilars. While the cost savings from introducing infliximab biosimilars have not fully met initial projections, it is anticipated that a larger number of adalimumab biosimilars will penetrate the market, potentially assisting in reducing costs. Additional strategies encompass task shifting in healthcare, such as leveraging IBD-specialized nurses more extensively, and the employment of digital technologies to streamline interactions and disease monitoring between patients and healthcare providers. Sustained research into personalized IBD therapeutics remains crucial [54].

The frontier analysis aims to assess the extent of the disease burden in Asian countries. In our analysis, many countries which have low SDI demonstrated a low disease burden. Conversely, countries with higher SDI, such as Japan, experienced a more significant IBD burden during the study period, necessitating targeted medical policies to ensure proper management of IBD [55–58]. Although the frontier analysis reveals a decreasing disease burden in most countries, most still possess the considerable potential to reduce IBD-related DALYs. Therefore, Asian countries and regions must devote increased attention to addressing IBD.

Considering demographic factors, our model anticipates an imminent increase in the burden of IBD in Asia and predicts that the Asia will transition to a composite epidemic phase around 2030 [59–61]. After 2035, the increasing incidence rates in East Asia will decline, transitioning from an Accelerated Incidence stage to a Compounding Prevalence stage [30]. The incidence in Southeast Asia, currently at a low level, will persistently rise and remain in the Emergence and Accelerated Incidence stages. Central Asia’s IBD incidence is relatively stable at a high level and has already reached the Compounding Prevalence stage, now transitioning to Prevalence Equilibrium [37].

Due to IBD's chronic and incurable nature, particularly in young people, prolonged periods of high morbidity and low mortality have led to a compounding disease epidemic in Western countries, despite stable or declining incidence rates [59]. This phenomenon suggests that for a lifelong disease such as IBD, prevalence steadily accumulates and increases wherever incidence surpasses mortality [3, 62]. In Asia, our projections indicate that IBD incidence will continue to rise until reaching a plateau by 2030. Additionally, high-income countries in Asia are already in the Compounding Prevalence stage and are transitioning to Prevalence Equilibrium [49, 56, 63]. Although the incidence has plateaued, the annual number of cases in high-income countries is expected to decrease, potentially due to demographic shifts resulting from declining fertility rates in these countries.

This study presents a comprehensive and long-term assessment of the burden of IBD in Asia, including incidence, prevalence, mortality, and DALYs from 1990 to 2019, as well as projections of incidence and mortality rates over the next 25 years. In comparison to the Global Burden of Disease Study 2017 (GBD2017), the Global Burden of Disease Study 2019 (GBD2019) incorporates revisions to the data from certain regions, thereby aligning the statistics more closely with the actual scenario. For instance, the prevalence rate for China, which was originally reported as 136.20 cases per 100,000 individuals in the GBD2017, was adjusted to 47.06 cases per 100,000 individuals in the GBD2019 report. However, there are some limitations to our study. Firstly, the GBD data do not differentiate between Crohn's disease and ulcerative colitis. Further analysis is necessary to separately investigate the epidemiological patterns of each subtype. Secondly, our study was constrained by the inherent limitations associated with Global Burden of Disease studies, which include the quality and accessibility of raw data, particularly concerning nonfatal outcomes. Despite the application of intricate statistical models by Global Burden of Disease studies to mitigate this issue, it remains imperative to bolster both the data collection processes and the model development to ensure the future availability of comprehensive, high-quality data.

## Conclusion

This study investigates the burden of IBD in Asia. Asia experiences many IBD cases compared to other continents, and incidence and prevalence rates are projected to continue rising. East Asia has exhibited the most rapid increase in incidence and prevalence, while Central Asia is expected to maintain the highest prevalence. Consequently, the burden of IBD in Asia will persistently escalate. Although ASMR and ASDR have decreased, there remains substantial potential for further reduction of ASDR across all countries, particularly in Japan, which necessitates prompt action to alleviate the IBD burden. Attention should be focused on the peak age of onset, between 30 and 45 years, and efforts should be made to enhance the training of IBD specialists, thereby enabling patients to access improved treatment options. Addressing the challenge of IBD in Asia demands international, multidimensional, and multisectoral collaboration.

### Supplementary Information

Below is the link to the electronic supplementary material.

Supplementary file1 (DOCX 3482 kb)

## Data Availability

Data can be obtained from the Global Health Data Exchange Global Burden of Disease Results Tool (https://ghdx.healthdata.org/gbd-results-tool).

## Abbreviations

GBD: Global Burden of Disease
IBD: Inflammatory bowel disease
EAPC: Estimated Annual Percentage Change
DALY: Disability-Adjusted Life Years
ASIR: Age-standardized incidence rates
ASPR: Age-standardized prevalence rates
ASMR: Age-standardized mortality rates
ASDR: Age-standardized DALY rates
APC: Annual percentage change
AAPC: Average annual percent change
