# Supplementary material for: Evolving Trends and Burden of Inflammatory Bowel Disease in Asia, 1990–2019: A Comprehensive Analysis Based on the Global Burden of Disease Study
Source: J Epidemiol Glob Health. 2023 Sep 1;13(4):725–39. doi: 10.1007/s44197-023-00145-w (PMC10686927; doi:10.1007/s44197-023-00145-w)
Supplement: Supplementary file 1 — Supplementary file1 (DOCX 3482 kb) [file 44197_2023_145_MOESM1_ESM.docx]

Supplementary appendix

Contents:

Appendix Figures:

Appendix figure 1. Trends from 1990 to 2019 in (A) ASIR; (B) ASPR; (C) ASMR of IBD in East, Central, South, Southeast Asia and Asia.

Appendix figure 2**.** Map of Asia regions: East Asia, Southeast Asia, West Asia, and Central Asia.

Appendix figure 3**.** Maps of age-standardized incidence rates of IBD in Asian countries and regions.

Appendix figure 4**.** Maps of age-standardized mortality rates of IBD in Asian countries and regions.

Appendix figure 5: The EAPCs map of IBD burden in Asian countries and regions. Abbreviations: EAPC, estimated annual percentage change; IBD, Inflammatory bowel disease.

Appendix figure 6. Joinpoint regression analysis of ASIR for IBD in Asia for both sex from 1990 to 2019. Abbreviations: IBD, Inflammatory bowel disease; ASIR, Age-standardized incidence rates.

Appendix figure 7. Joinpoint regression analysis of ASPR for IBD in Asia for both sex from 1990 to 2019. Abbreviations: IBD, Inflammatory bowel disease; ASPR, Age-standardized prevalence rates.

Appendix figure 8. Joinpoint regression analysis of ASMR for IBD in Asia for both sex from 1990 to 2019. Abbreviations: IBD, Inflammatory bowel disease; ASIR, Age-standardized mortality rates.

Appendix figure 9. Joinpoint regression analysis of ASIR for IBD in Asia for male from 1990 to 2019. Abbreviations: IBD, Inflammatory bowel disease; ASIR, Age-standardized incidence rates.

Appendix figure 10. Joinpoint regression analysis of ASPR for IBD in Asia for male from 1990 to 2019. Abbreviations: IBD, Inflammatory bowel disease; ASPR, Age-standardized prevalence rates.

Appendix figure 11. Joinpoint regression analysis of ASMR for IBD in Asia for male from 1990 to 2019. Abbreviations: IBD, Inflammatory bowel disease; ASMR, Age-standardized mortality rates.

Appendix figure 12. Joinpoint regression analysis of ASIR for IBD in Asia for female from 1990 to 2019. Abbreviations: IBD, Inflammatory bowel disease; ASIR, Age-standardized incidence rates.

Appendix figure 13. Joinpoint regression analysis of ASPR for IBD in Asia for female from 1990 to 2019. Abbreviations: IBD, Inflammatory bowel disease; ASPR, Age-standardized prevalence rates.

Appendix figure 14. Joinpoint regression analysis of ASMR for IBD in Asia for female from 1990 to 2019. Abbreviations: IBD, Inflammatory bowel disease; ASMR, Age-standardized mortality rates.

Appendix figure 15. Age-period-cohort effects for East Asia (A): Age effect (B): Period effect (C): Cohort effect (D): Local drift and net drift

Appendix figure 16. Age-period-cohort effects for Southeast Asia (A): Age effect (B): Period effect (C): Cohort effect (D): Local drift and net drift

Appendix figure 17. Age-period-cohort effects for South Asia (A): Age effect (B): Period effect (C): Cohort effect (D): Local drift and net drift

Appendix figure 18. Age-period-cohort effects for Central Asia (A): Age effect (B): Period effect (C): Cohort effect (D): Local drift and net drift

Appendix figure 19. Changes in IBD burden across Asian countries from 1990 to 2019, based on Frontier analysis.

Appendix figure 20. Changes in (A) incidence and (B) mortality in Asia, East Asia, Southeast Asia, South Asia, Central Asia, High-income regions over the next 25 years, based on Norepord.

Appendix Tables:

Appendix table 1. IBD incidence in 1990 and 2019 for both sexes in age-standardised rates by location in Asia.

Appendix table 2. IBD prevalence in 1990 and 2019 for both sexes in age-standardised rates by location in Asia.

Appendix table 3. IBD deaths in 1990 and 2019 for both sexes in age-standardised rates by location in Asia.

Appendix table 4. IBD DALY in 1990 and 2019 for both sexes in age-standardised rates by location in Asia.

Appendix table 5. Joinpoint regression analysis of ASIR, ASPR, ASDR and ASMR for IBD in Asia from 1990 to 2019.

Appendix table 6. Long age of APC analysis across to different regions.

Appendix table 7. Period RR of APC analysis across to different regions.

Appendix table 8. Cohort RR of APC analysis across to different regions.

Appendix table 9. Net drift and Local drift of APC analysis across to different regions.

Appendix table 10. Changes in incidence number according to population-level determinants and causes from 1990 to 2019 across to location

Appendix table 11. Changes in deaths number according to population-level determinants and causes from 1990 to 2019 across to location

Appendix table 12. Frontier analysis of 52 Asia countries.

Appendix table 13. Incidence and Deaths projections of inflammatory bowel disease across different regions from 2019 to 2044.


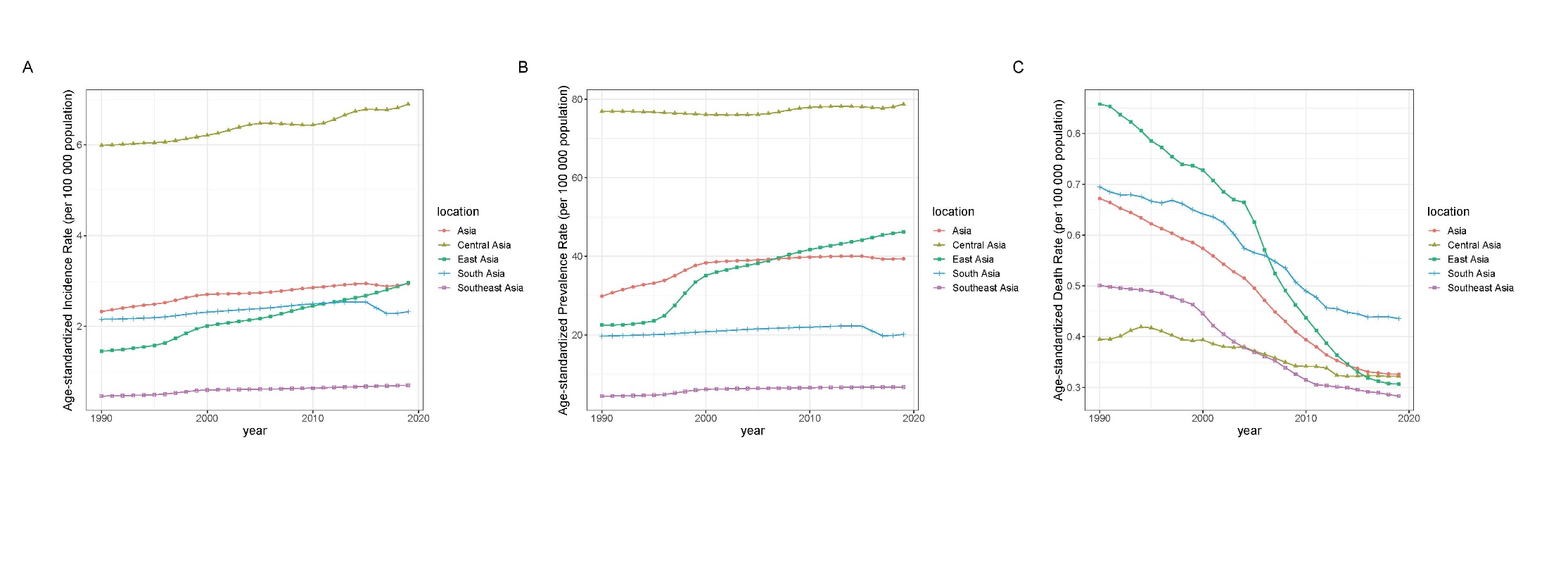


Appendix figure 1. Trends from 1990 to 2019 in (A) ASIR; (B) ASPR; (C) ASMR of IBD in East, Central, South, Southeast Asia and Asia. Abbreviations: IBD, Inflammatory bowel disease; DALY, disability-adjusted life year; ASIR, Age-standardized incidence rates; ASPR, Age-standardized prevalence rates; ASDR, Age-standardized DALY rates; ASMR, Age-standardized mortality rates.


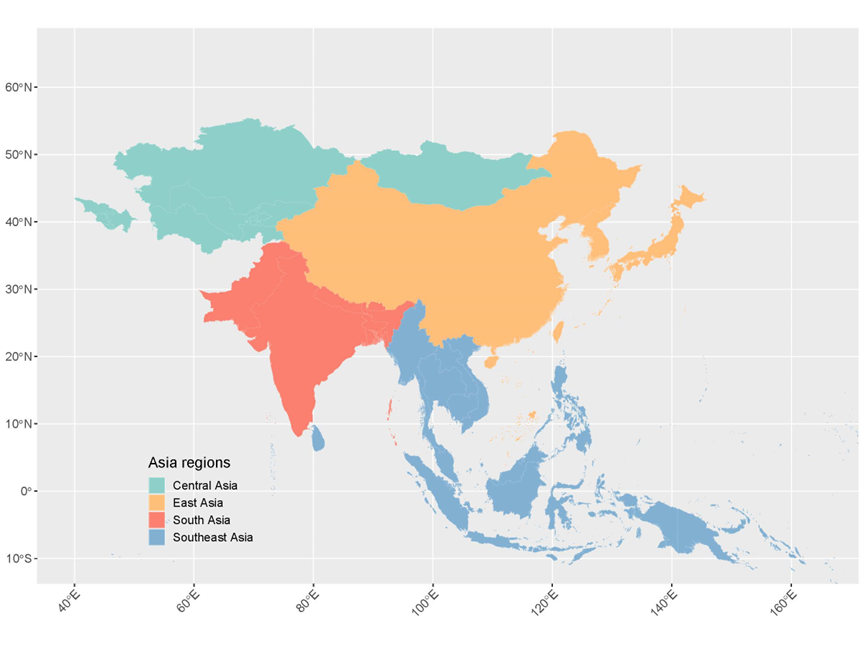


Appendix figure 2. Map of Asia regions: East Asia, Southeast Asia, West Asia, and Central Asia.
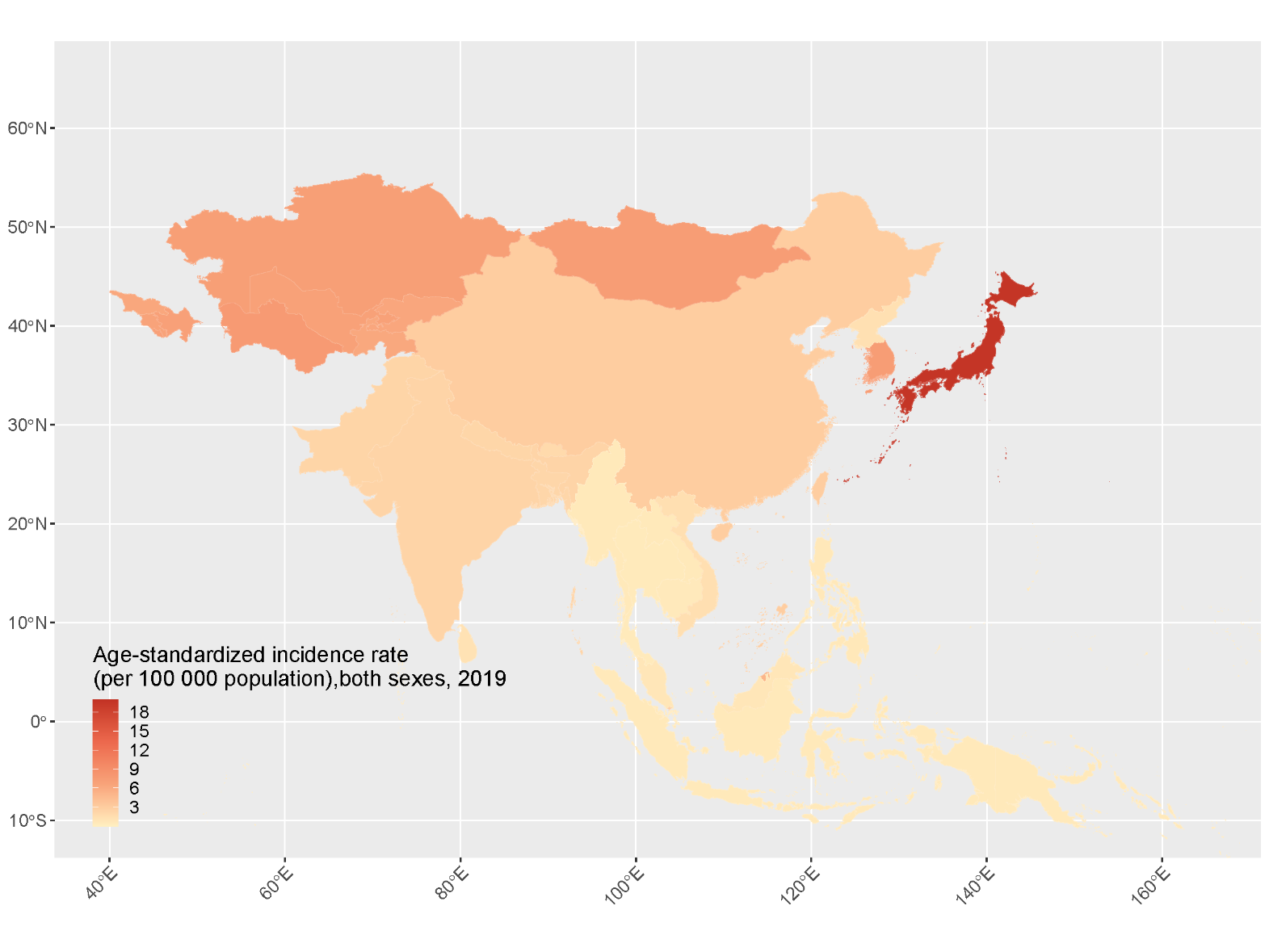


Appendix figure 3. Maps of age-standardized incidence rates of IBD in Asian countries and regions.


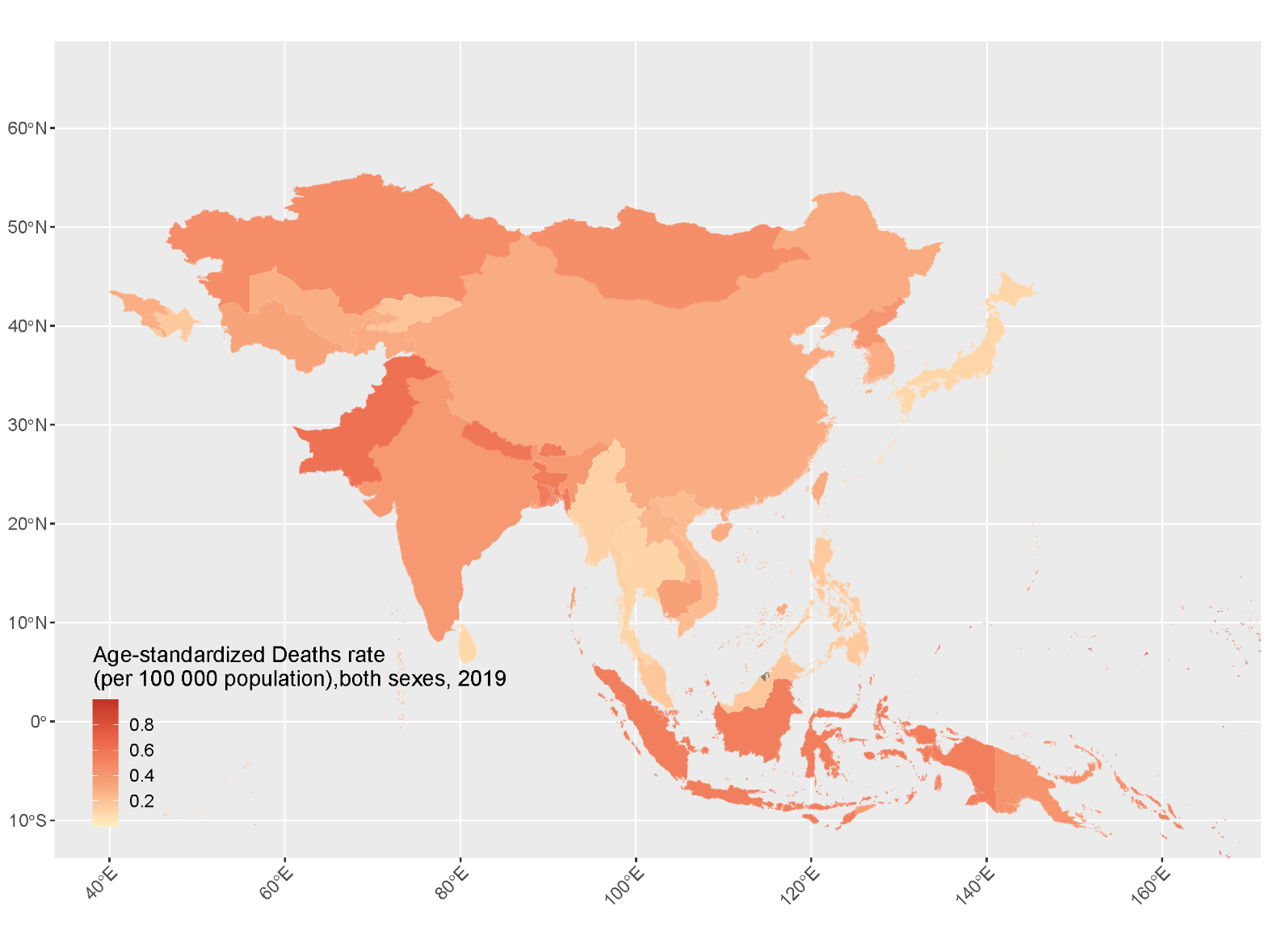


Appendix figure 4. Maps of age-standardized mortality rates of IBD in Asian countries and regions.


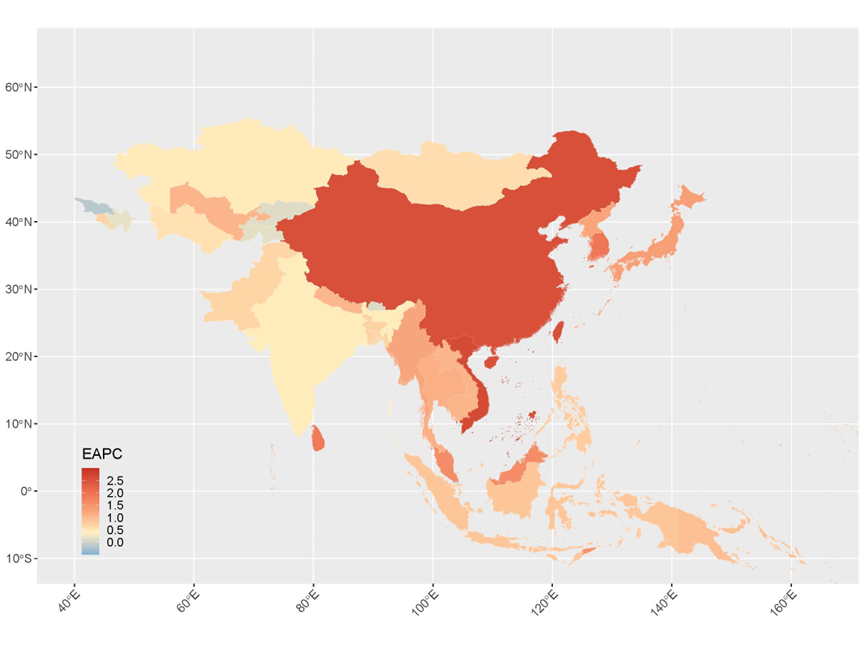


Appendix figure 5: The EAPCs map of IBD burden in Asian countries and regions. Abbreviations: EAPC, estimated annual percentage change; IBD, Inflammatory bowel disease.


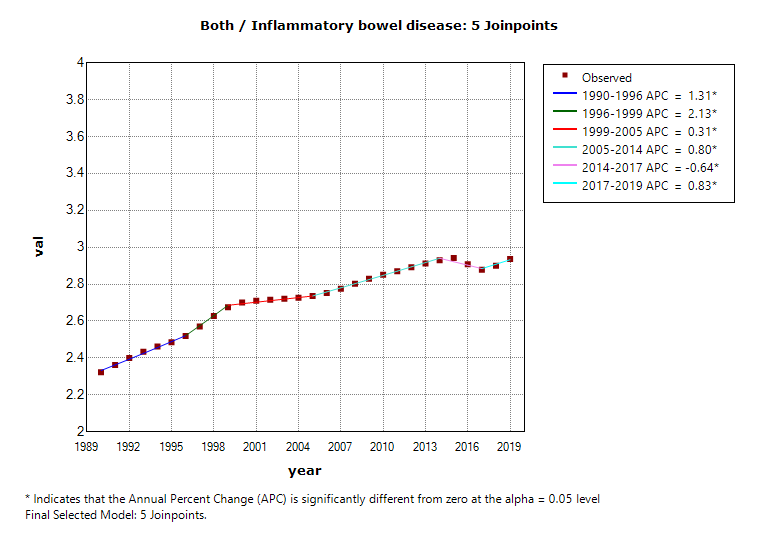


Appendix figure 6. Joinpoint regression analysis of ASIR for IBD in Asia for both sex from 1990 to 2019. Abbreviations: IBD, Inflammatory bowel disease; ASIR, Age-standardized incidence rates.


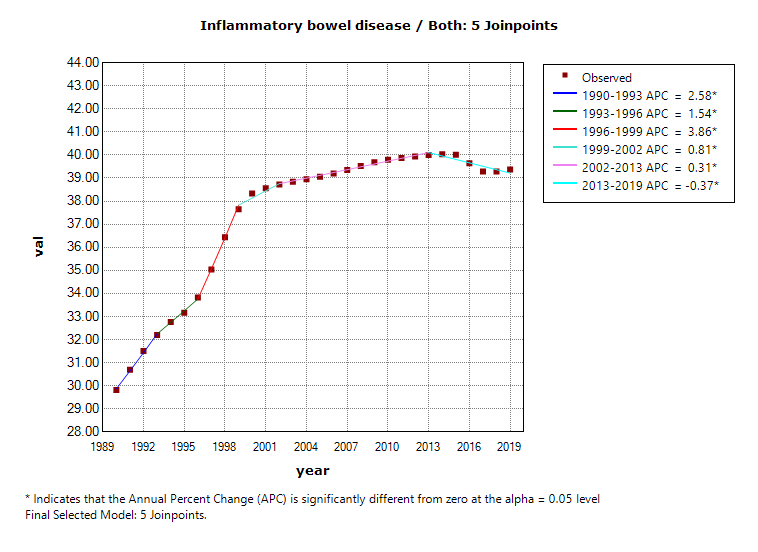


Appendix figure 7. Joinpoint regression analysis of ASPR for IBD in Asia for both sex from 1990 to 2019. Abbreviations: IBD, Inflammatory bowel disease; ASPR, Age-standardized prevalence rates.


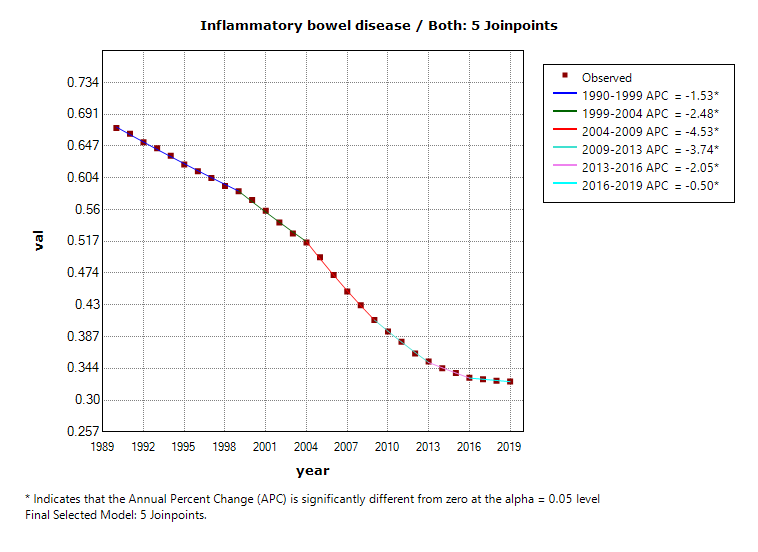


Appendix figure 8. Joinpoint regression analysis of ASMR for IBD in Asia for both sex from 1990 to 2019. Abbreviations: IBD, Inflammatory bowel disease; ASMR, Age-standardized mortality rates.


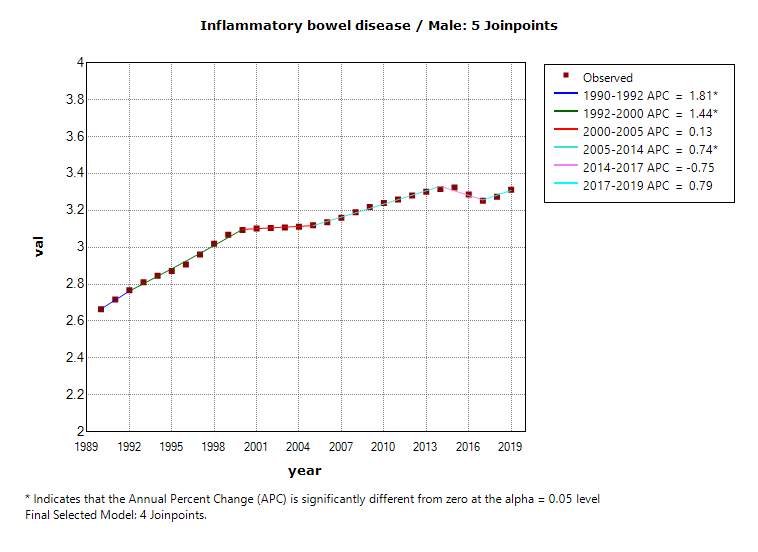


Appendix figure 9. Joinpoint regression analysis of ASIR for IBD in Asia for male from 1990 to 2019. Abbreviations: IBD, Inflammatory bowel disease; ASIR, Age-standardized incidence rates.


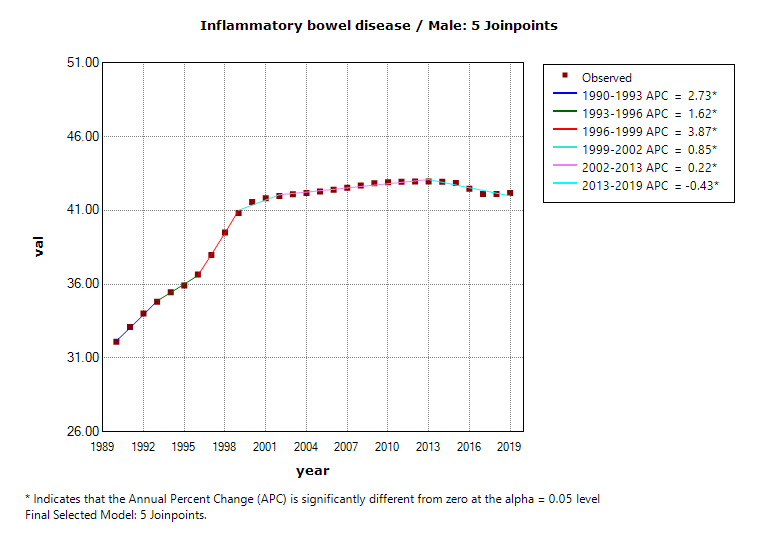


Appendix figure 10. Joinpoint regression analysis of ASPR for IBD in Asia for male from 1990 to 2019. Abbreviations: IBD, Inflammatory bowel disease; ASPR, Age-standardized prevalence rates.


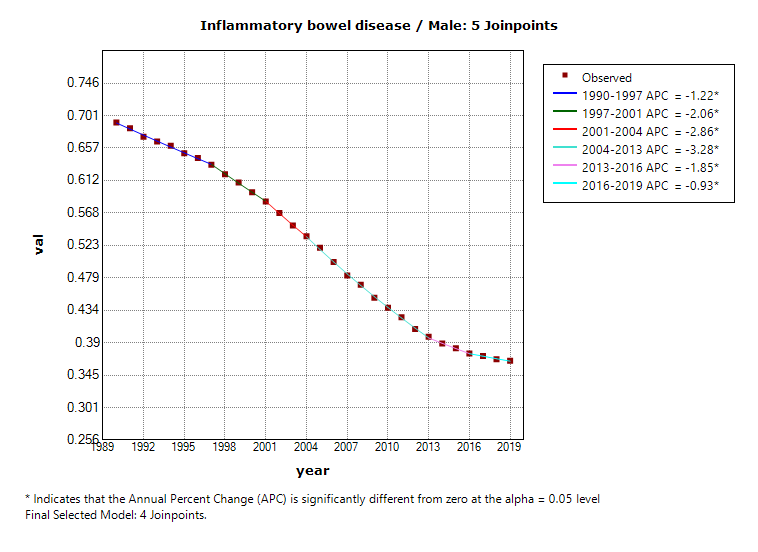


Appendix figure 11. Joinpoint regression analysis of ASMR for IBD in Asia for male from 1990 to 2019. Abbreviations: IBD, Inflammatory bowel disease; ASMR, Age-standardized mortality rates.


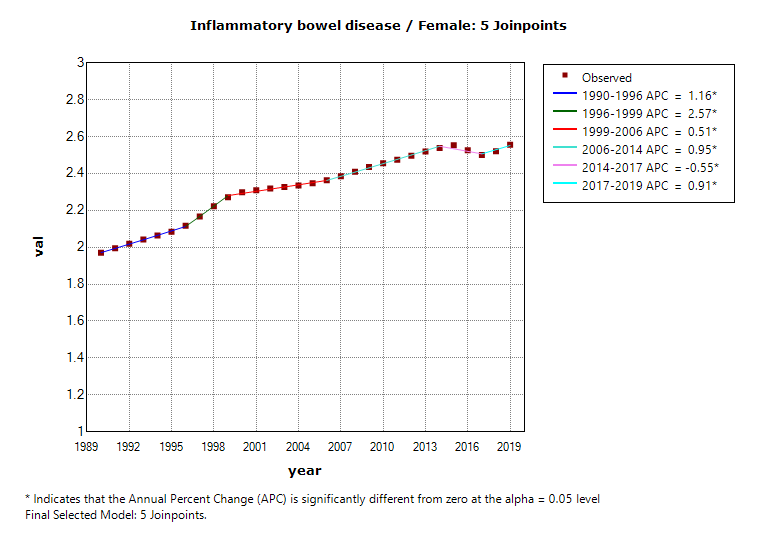


Appendix figure 12. Joinpoint regression analysis of ASIR for IBD in Asia for female from 1990 to 2019. Abbreviations: IBD, Inflammatory bowel disease; ASIR, Age-standardized incidence rates.


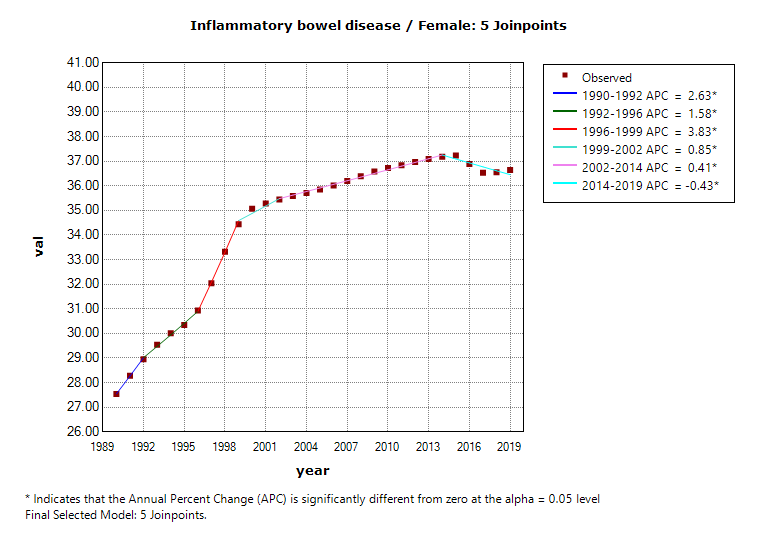


Appendix figure 13. Joinpoint regression analysis of ASPR for IBD in Asia for female from 1990 to 2019. Abbreviations: IBD, Inflammatory bowel disease; ASPR, Age-standardized prevalence rates.


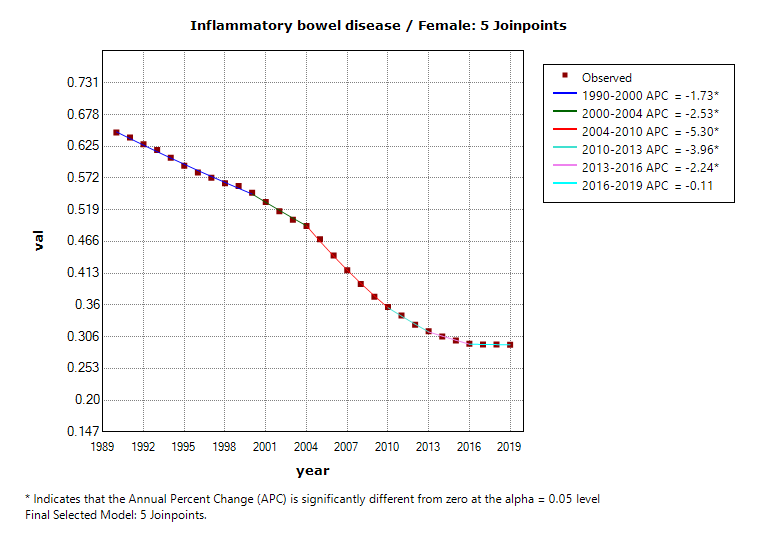


Appendix figure 14. Joinpoint regression analysis of ASMR for IBD in Asia for female from 1990 to 2019. Abbreviations: IBD, Inflammatory bowel disease; ASMR, Age-standardized mortality rates.


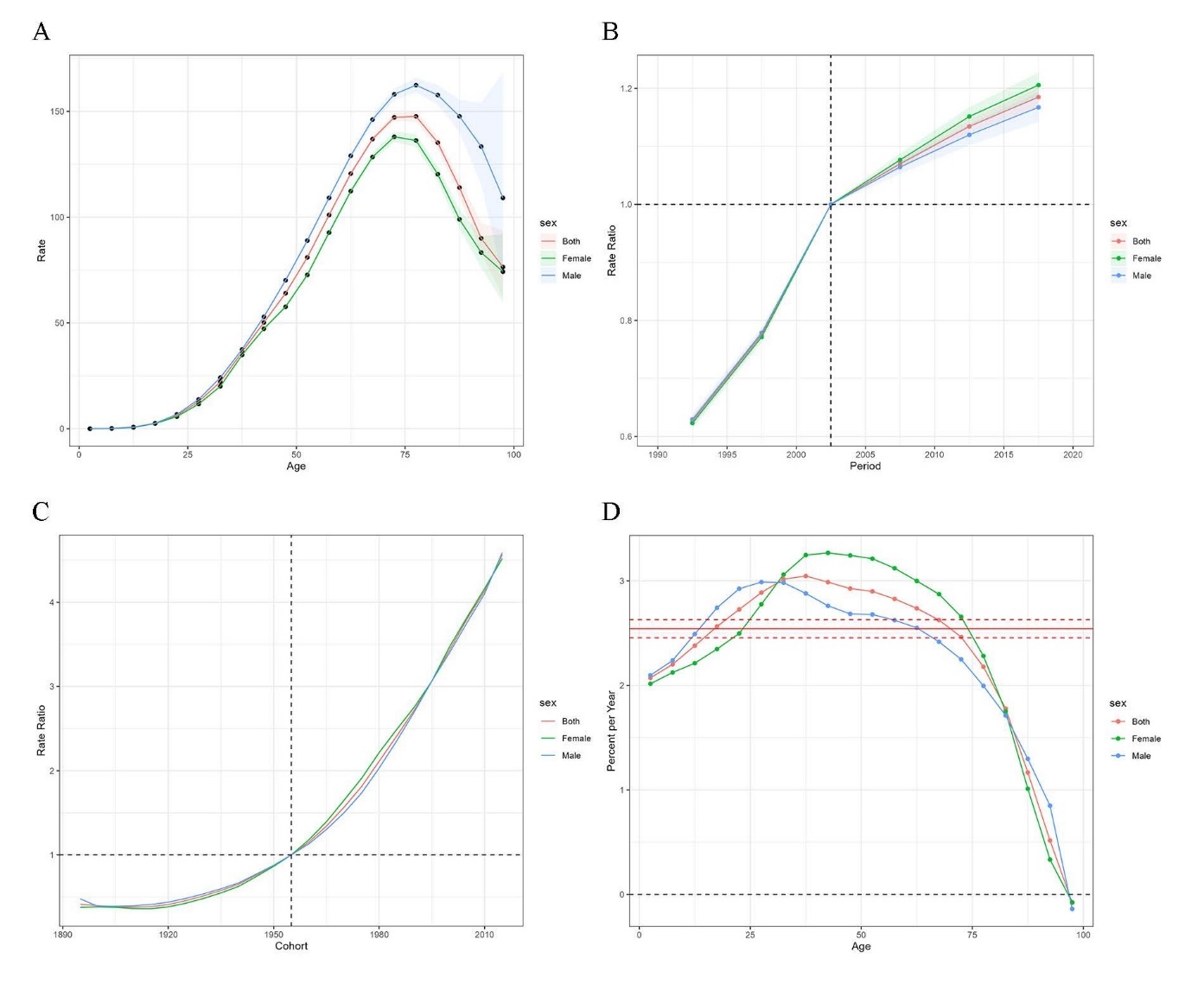


Appendix figure 15. Age-period-cohort effects for East Asia (A): Age effect (B): Period effect (C): Cohort effect (D): Local drift and net drift


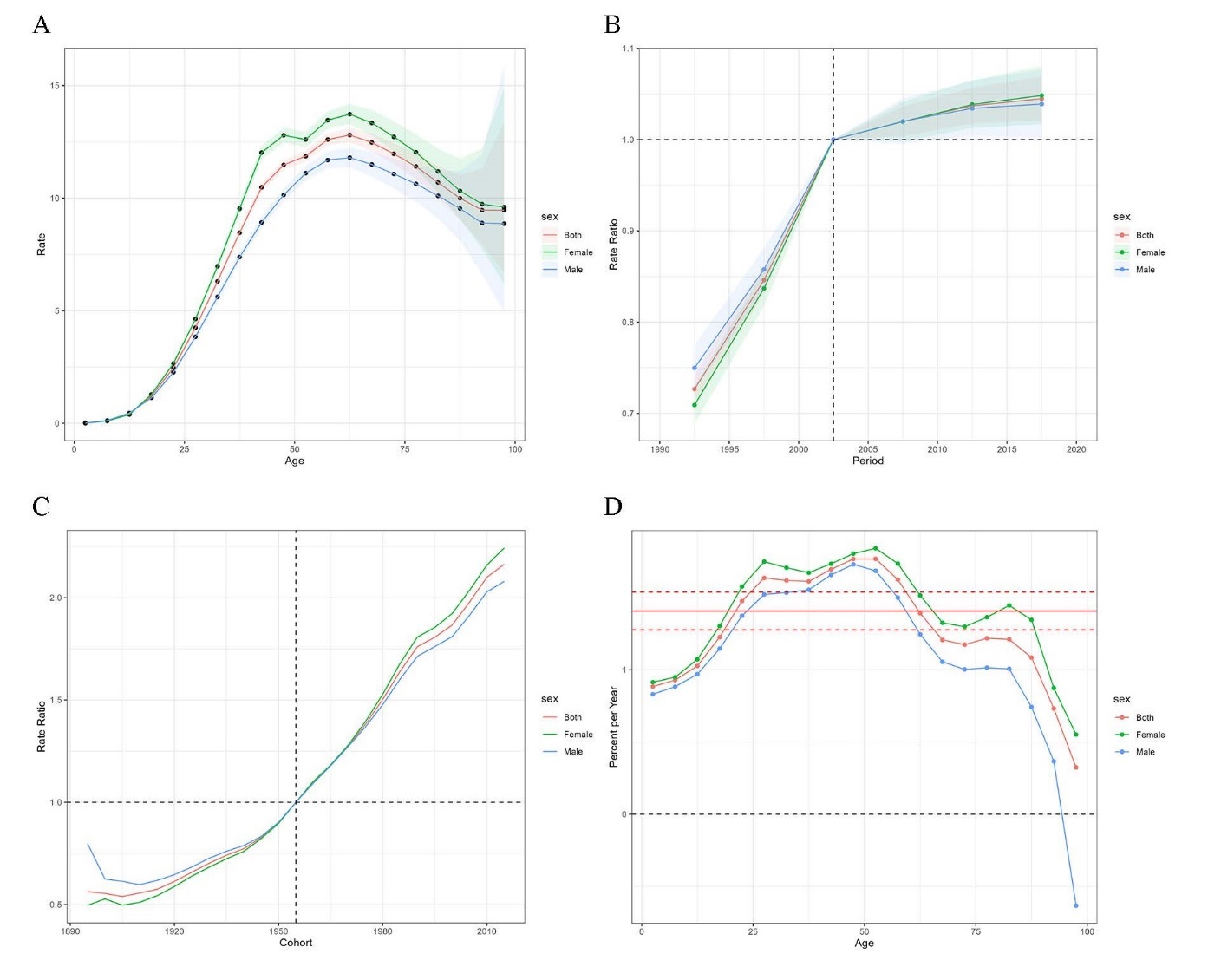


Appendix figure 16. Age-period-cohort effects for Southeast Asia (A): Age effect (B): Period effect (C): Cohort effect (D): Local drift and net drift


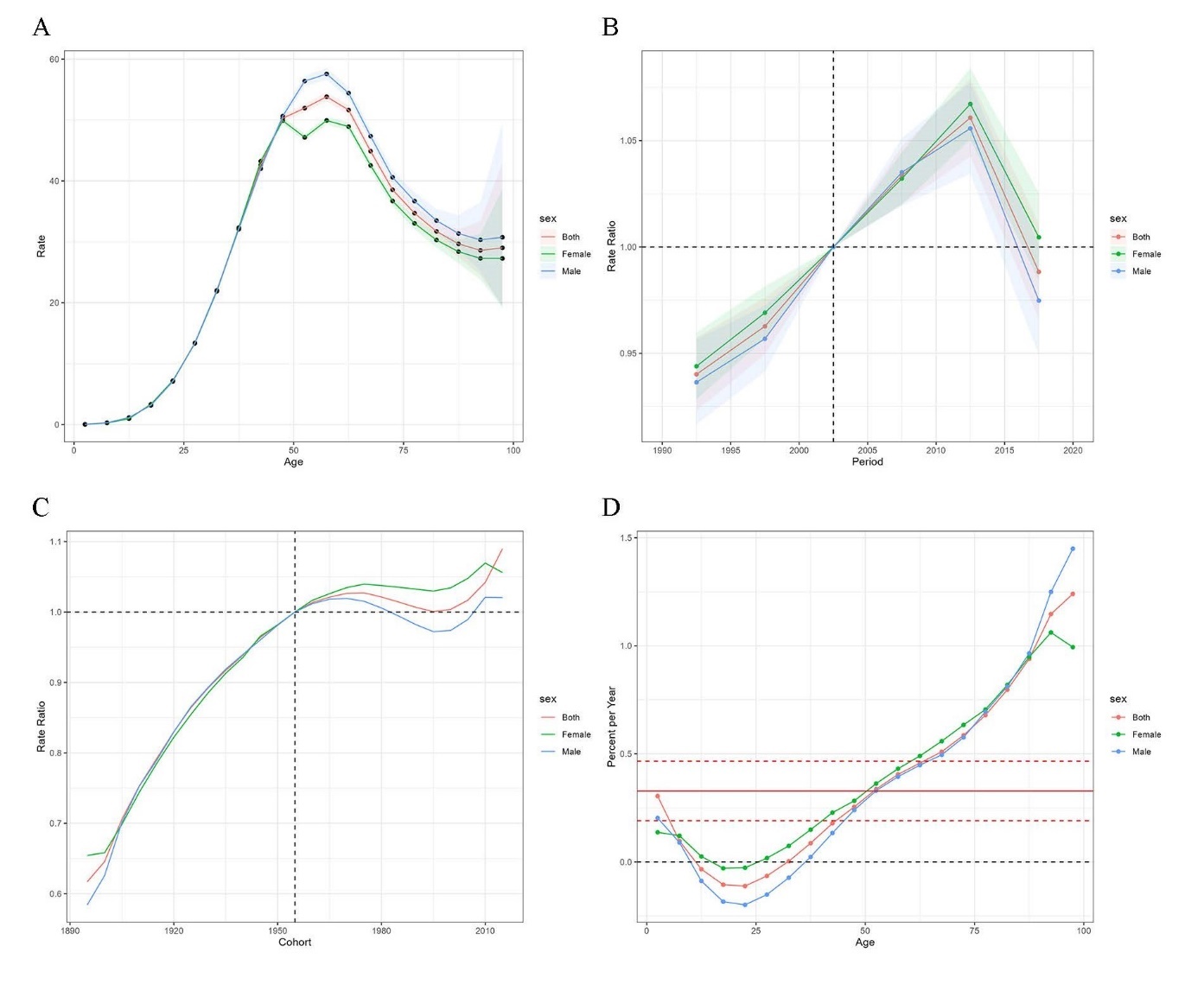


Appendix figure 17. Age-period-cohort effects for South Asia (A): Age effect (B): Period effect (C): Cohort effect (D): Local drift and net drift


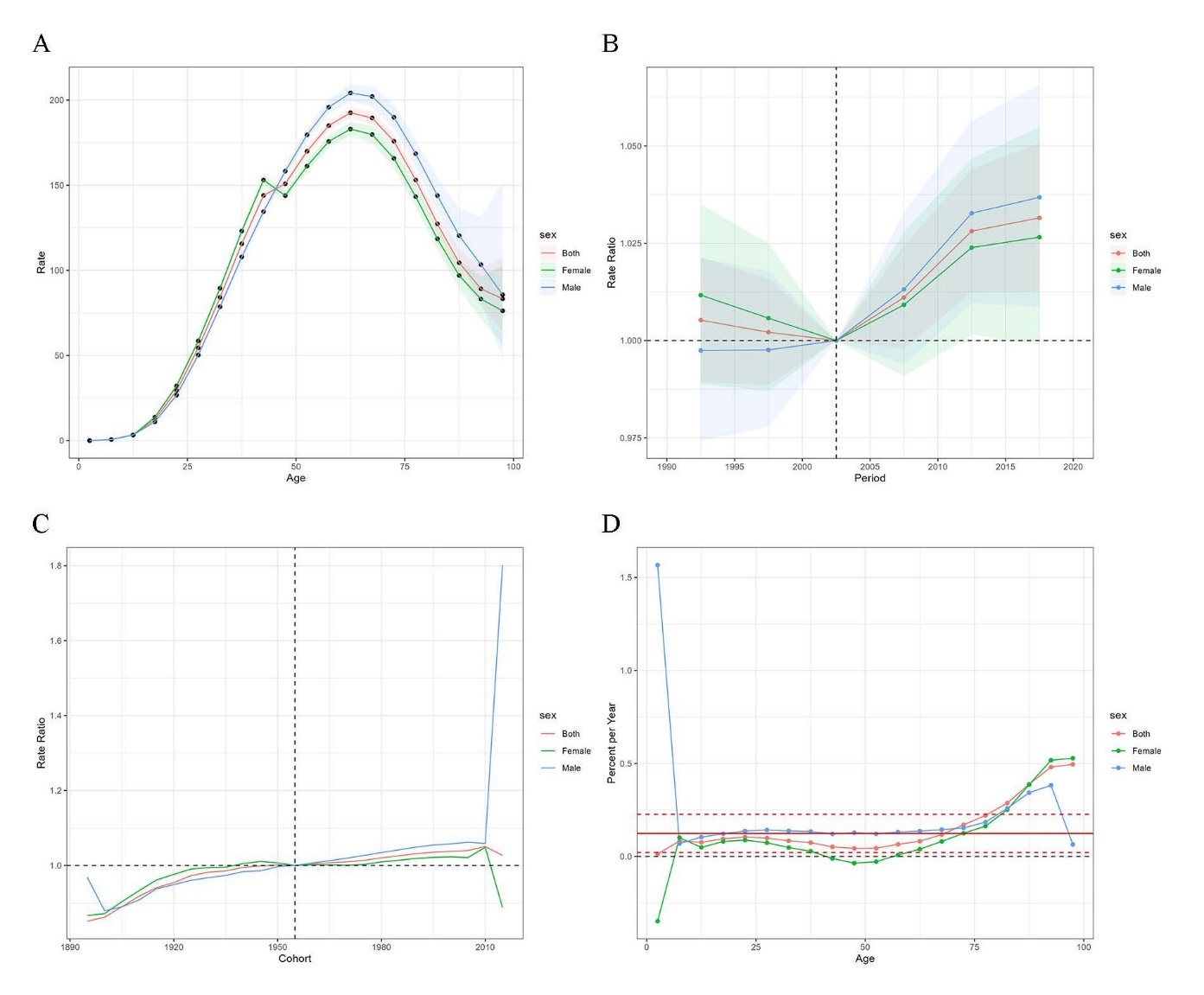


Appendix figure 18. Age-period-cohort effects for Central Asia (A): Age effect (B): Period effect (C): Cohort effect (D): Local drift and net drift


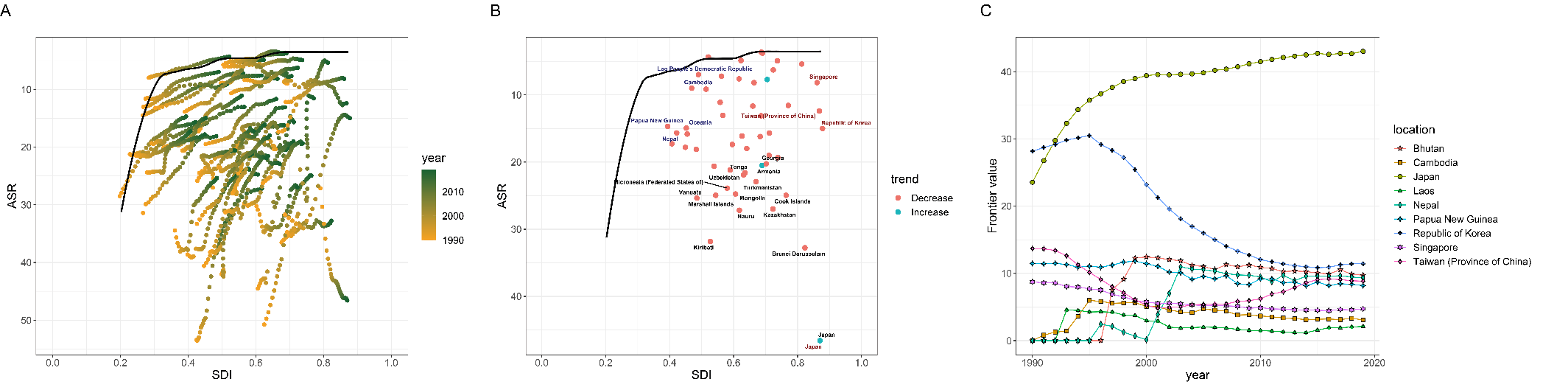


Appendix figure 19. **Changes in IBD burden across Asian countries from 1990 to 2019, based on Frontier analysis.** (A) Frontier analysis based on Sociodemographic Index (SDI) and age-standardized IBD disability-adjusted life-years (DALY) rate from 1990 to 2019. Color scale represents the years from 1990 depicted in yellow to 2019 depicted in green. The frontier is delineated in solid black color. (B) Frontier analysis based on SDI and age-standardized IBD DALY rate in 2019. The frontier is delineated in solid black color; countries and territories are represented as dots. The top 15 countries with the largest effective difference are marked with black; Low SDI (< 0.5) and examples of border countries with low effective differences are highlighted in blue (e.g., Laos, Cambodia, Papua New Guinea, Nepal, Bhutan), high SDI (> 0.85) and examples of countries and regions with relatively high effective differences relative to their level of development are highlighted in red (e.g., Japan, South Korea, Taiwan, Singapore). The red dots represent the increase in the age-standardized IBD DALY rate from 1990 to 2019; The blue dots represent the decline in the age-standardized IBD DALY rate between 1990 and 2019.


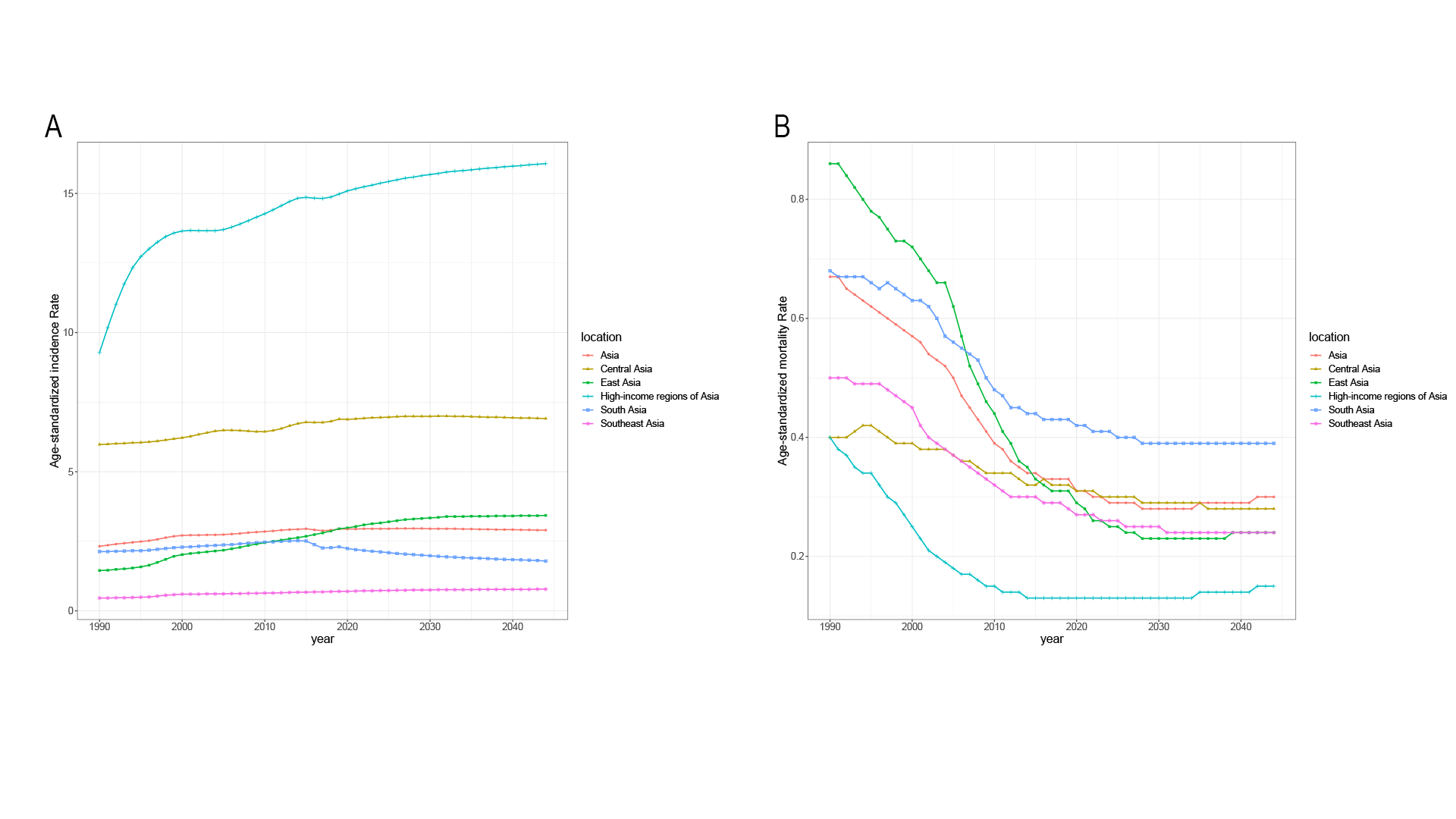


Appendix figure 20. Changes in (A) incidence and (B) mortality in Asia, East Asia, Southeast Asia, South Asia, Central Asia, High-income regions over the next 25 years, based on Norepord.

Appendix table 1. IBD incidence in 1990 and 2019 for both sexes in age-standardised rates by location in Asia.

| Location | 1990 | | 2019 | |
| --- | --- | --- | --- | --- |
|  | Counts (95% UI) | Rate (95% UI) | Counts (95% UI) | Rate (95% UI) |
| Asia | 64,767.7 (54,186.15, 76,818.52) | 2.32 (1.97, 2.76) | 145,560.68 (124,960.26, 170,894.99) | 2.94 (2.53, 3.44) |
| East Asia | 17,567.28 (14,626.86, 20,802.38) | 1.45 (1.22, 1.71) | 52,318.76 (44,680.37, 61,454.76) | 2.95 (2.55, 3.43) |
| South Asia | 18,439.38 (15,168.63, 22,581.85) | 2.15 (1.79, 2.64) | 39,704.7 (32,943.42, 48,453.53) | 2.32 (1.93, 2.84) |
| Southeast Asia | 1,881.96 (1,527.13, 2,270.35) | 0.46 (0.38, 0.56) | 4,979.99 (4,181.77, 5,972.30) | 0.7 (0.59, 0.84) |
| Central Asia | 3,514.12 (2,994.99, 4,146.25) | 5.99 (5.13, 7.07) | 6,461.31 (5,487.21, 7,636.70) | 6.9 (5.87, 8.13) |
| High-income Asia Pacific | 18105.05 (15391.42, 21421.78) | 9.25 (7.88, 10.91) | 27083.76 (23870.3, 30621.12) | 14.93 (13.14, 16.93) |
| American Samoa | 0.19 (0.16, 0.23) | 0.49 (0.41, 0.58) | 0.34 (0.29, 0.41) | 0.64 (0.54, 0.77) |
| Armenia | 178.48 (149.63, 209.32) | 5.48 (4.6, 6.46) | 236.7 (200.07, 282.62) | 6.64 (5.62, 7.95) |
| Azerbaijan | 431.54 (364.73, 510.13) | 6.74 (5.74, 7.97) | 804.68 (676.92, 965.26) | 7.01 (5.92, 8.4) |
| Bangladesh | 1,355.12 (1,104.55, 1,656.00) | 1.76 (1.45, 2.17) | 3,404.18 (2,788.48, 4,136.27) | 2.17 (1.78, 2.65) |
| Bhutan | 7.5 (6.02, 9.33) | 1.68 (1.38, 2.11) | 14.13 (11.68, 17.36) | 1.86 (1.55, 2.29) |
| Brunei Darussalam | 12.69 (10.24, 15.26) | 4.95 (4.11, 5.97) | 25.73 (20.77, 30.85) | 5.09 (4.17, 6.09) |
| Cambodia | 30.09 (23.48, 36.88) | 0.37 (0.3, 0.45) | 79.3 (64.29, 96.04) | 0.49 (0.4, 0.59) |
| China | 17,221.41 (14,326.45, 20,399.48) | 1.47 (1.24, 1.74) | 51,461.96 (43,932.97, 60,474.47) | 3.01 (2.59, 3.5) |
| Cook Islands | 0.09 (0.07, 0.11) | 0.54 (0.45, 0.64) | 0.15 (0.13, 0.18) | 0.74 (0.63, 0.9) |
| Democratic People's Republic of Korea | 196.52 (163.97, 236.26) | 0.96 (0.8, 1.15) | 387.24 (326.12, 462.93) | 1.29 (1.08, 1.53) |
| Fiji | 3.41 (2.77, 4.13) | 0.54 (0.44, 0.64) | 6.5 (5.46, 7.87) | 0.72 (0.61, 0.86) |
| Georgia | 381.42 (324.63, 448.9) | 6.48 (5.54, 7.62) | 275.43 (233.52, 326.56) | 6.32 (5.38, 7.44) |
| Guam | 0.7 (0.58, 0.84) | 0.55 (0.46, 0.66) | 1.34 (1.13, 1.59) | 0.75 (0.64, 0.9) |
| India | 15,360.33 (12,630.92, 18,814.76) | 2.23 (1.85, 2.73) | 31,774.51 (26,432.57, 38,972.12) | 2.34 (1.95, 2.86) |
| Indonesia | 681.12 (551.3, 828.13) | 0.43 (0.36, 0.52) | 1504.03 (1248.01, 1808.48) | 0.55 (0.46, 0.66) |
| Japan | 16,382.81 (13,832.98, 19,566.68) | 11.22 (9.49, 13.32) | 22,435.8 (19,353.24, 25,906.09) | 19.65 (16.87, 22.71) |
| Kazakhstan | 922.22 (781.91, 1089.31) | 6.01 (5.11, 7.11) | 1388.99 (1185.49, 1660.38) | 7.2 (6.14, 8.56) |
| Kiribati | 0.29 (0.23, 0.35) | 0.48 (0.4, 0.58) | 0.6 (0.49, 0.74) | 0.58 (0.48, 0.7) |
| Kyrgyzstan | 221.64 (188.52, 260.21) | 6.03 (5.14, 7.11) | 390.26 (329.08, 460.34) | 6.39 (5.41, 7.54) |
| Lao People's Democratic Republic | 12.49 (9.81, 15.38) | 0.37 (0.3, 0.46) | 35 (28.55, 43.28) | 0.51 (0.42, 0.63) |
| Malaysia | 72.05 (61.47, 82.81) | 0.46 (0.39, 0.52) | 251.19 (222.68, 283.41) | 0.76 (0.67, 0.85) |
| Maldives | 0.58 (0.46, 0.7) | 0.34 (0.28, 0.41) | 2.84 (2.29, 3.46) | 0.51 (0.43, 0.61) |
| Marshall Islands | 0.15 (0.12, 0.18) | 0.46 (0.38, 0.55) | 0.33 (0.27, 0.40) | 0.63 (0.53, 0.76) |
| Mauritius | 4.96 (4.02, 6.02) | 0.46 (0.38, 0.55) | 8.8 (7.41, 10.49) | 0.59 (0.5, 0.71) |
| Micronesia (Federated States of) | 0.38 (0.31, 0.46) | 0.49 (0.41, 0.59) | 0.64 (0.53, 0.77) | 0.67 (0.56, 0.81) |
| Mongolia | 97.76 (82.38, 115.55) | 6.23 (5.27, 7.49) | 251.06 (210.09, 300.12) | 7.35 (6.2, 8.69) |
| Myanmar | 141.32 (112.83, 173.2) | 0.4 (0.32, 0.49) | 306.41 (253.16, 377.81) | 0.55 (0.46, 0.68) |
| Nauru | 0.04 (0.03, 0.05) | 0.47 (0.39, 0.57) | 0.06 (0.05, 0.07) | 0.64 (0.54, 0.77) |
| Nepal | 242.04 (197.56, 300.94) | 1.66 (1.37, 2.07) | 624.25 (515.19, 764.14) | 2.24 (1.85, 2.76) |
| Niue | 0.01 (0.01, 0.01) | 0.51 (0.43, 0.62) | 0.01 (0.01, 0.02) | 0.73 (0.61, 0.87) |
| Northern Mariana Islands | 0.26 (0.21, 0.31) | 0.6 (0.5, 0.71) | 0.38 (0.32, 0.45) | 0.74 (0.63, 0.89) |
| Pakistan | 1,474.39 (1,218.12, 1,792.03) | 1.91 (1.59, 2.34) | 3,887.63 (3,192.87, 4,838.46) | 2.3 (1.91, 2.86) |
| Palau | 0.08 (0.07, 0.1) | 0.56 (0.47, 0.66) | 0.17 (0.14, 0.2) | 0.76 (0.64, 0.91) |
| Papua New Guinea | 13.07 (10.55, 15.63) | 0.4 (0.33, 0.48) | 43.44 (35.51, 52.8) | 0.52 (0.44, 0.64) |
| Philippines | 242.31 (196.7, 292.41) | 0.48 (0.39, 0.58) | 656.6 (541.1, 799.48) | 0.63 (0.52, 0.76) |
| Republic of Korea | 1,586.68 (1,390.2, 1,776.81) | 3.37 (2.96, 3.78) | 4,407.24 (4,219.72, 4,589.27) | 7.33 (7.03, 7.63) |
| Samoa | 0.6 (0.49, 0.72) | 0.46 (0.38, 0.55) | 1.13 (0.94, 1.35) | 0.6 (0.51, 0.73) |
| Seychelles | 0.33 (0.27, 0.4) | 0.49 (0.4, 0.59) | 0.7 (0.58, 0.84) | 0.61 (0.51, 0.73) |
| Singapore | 122.86 (100.52, 146.67) | 3.47 (2.87, 4.13) | 214.98 (177.87, 263.66) | 3.21 (2.68, 3.9) |
| Solomon Islands | 1.05 (0.85, 1.26) | 0.43 (0.36, 0.52) | 2.96 (2.39, 3.67) | 0.56 (0.46, 0.68) |
| Sri Lanka | 106.87 (87.73, 130.59) | 0.67 (0.56, 0.81) | 267.83 (238.71, 301.46) | 1.15 (1.02, 1.3) |
| Taiwan (Province of China) | 149.36 (129.38, 170.61) | 0.71 (0.62, 0.81) | 469.55 (420.46, 526.18) | 1.72 (1.54, 1.93) |
| Tajikistan | 225.24 (190.38, 266.63) | 5.83 (4.91, 6.93) | 518.41 (439.18, 611.95) | 6.23 (5.32, 7.3) |
| Thailand | 183.24 (142.17, 230.29) | 0.33 (0.26, 0.41) | 373.23 (309.33, 455.11) | 0.46 (0.38, 0.56) |
| Timor-Leste | 2.3 (1.81, 2.82) | 0.36 (0.29, 0.45) | 5.83 (4.72, 7.07) | 0.51 (0.42, 0.62) |
| Tokelau | 0.01 (0.01, 0.01) | 0.51 (0.43, 0.61) | 0.01 (0.01, 0.01) | 0.64 (0.54, 0.78) |
| Tonga | 0.34 (0.28, 0.41) | 0.45 (0.38, 0.53) | 0.59 (0.49, 0.71) | 0.66 (0.55, 0.78) |
| Turkmenistan | 187.71 (157.34, 222.18) | 6.75 (5.73, 7.99) | 382.78 (324.26, 450.53) | 7.68 (6.53, 9.05) |
| Tuvalu | 0.04 (0.03, 0.05) | 0.48 (0.4, 0.58) | 0.08 (0.07, 0.1) | 0.69 (0.57, 0.83) |
| Uzbekistan | 868.11 (731.72, 1,029.74) | 5.44 (4.62, 6.45) | 2,213 (1,878.26, 2,642.30) | 6.9 (5.9, 8.2) |
| Vanuatu | 0.51 (0.41, 0.62) | 0.45 (0.37, 0.55) | 1.46 (1.19, 1.77) | 0.59 (0.49, 0.71) |
| Viet Nam | 401.81 (315.52, 499.34) | 0.66 (0.53, 0.82) | 1,481.73 (1,200.76, 1,836.17) | 1.37 (1.11, 1.7) |

Appendix table 2. IBD prevalence in 1990 and 2019 for both sexes in age-standardised rates by location in Asia.

| Location | 1990 | | 2019 | |
| --- | --- | --- | --- | --- |
|  | Counts (95% UI) | Rate (95% UI) | Counts (95% UI) | Rate (95% UI) |
| Asia | 763,543.98 (634,873.91, 905,118.76) | 29.81 (24.93, 35.53) | 1,992,221.48 (1,707,091.60, 2,322,097.72) | 39.37 (33.70, 45.81) |
| East Asia | 244,790.11 (202,981.06, 291,054.01) | 22.51 (18.72, 26.72) | 925,073.73 (788,347.43, 1,085,237.55) | 46.22 (39.36, 53.98) |
| South Asia | 157,942.31 (127,076.74, 195,838.91) | 19.68 (15.98, 24.40) | 335,745.87 (272,329.97, 413,945.87) | 20.11 (16.38, 24.81) |
| Southeast Asia | 16,556.66 (13,040.43, 20,323.20) | 4.40 (3.55, 5.35) | 48,151.70 (3,9527.16, 58,062.71) | 6.69 (5.52, 8.04) |
| Central Asia | 42,099.57 (35,492.66, 49,944.35) | 76.86 (64.98, 91.56) | 72,033.98 (60,488.84, 86,073.07) | 78.72 (66.39, 93.40) |
| High-income Asia Pacific | 255,028.45 (213,480.49, 302,122.71) | 126.18 (105.73, 149.32) | 459,062.11 (403,027.23, 520,417.76) | 210.54 (183.69, 239.68) |
| American Samoa | 2.17 (1.76, 2.61) | 6.24 (5.14, 7.41) | 3.23 (2.65, 3.89) | 6.14 (5.03, 7.38) |
| Armenia | 2,546.77 (2,108.97, 3,013.19) | 81.15 (67.51, 95.76) | 3,262.72 (2,743.82, 3,956.31) | 86.67 (72.86, 104.56) |
| Azerbaijan | 4,622.64 (3,817.01, 5,491.03) | 76.23 (63.23, 90.47) | 9,462.94 (7,886.09, 11,457.75) | 81.93 (68.62, 99.02) |
| Bangladesh | 11,786.03 (9,494.47, 14,655.41) | 17.19 (13.9, 21.66) | 33,017.69 (26,284.98, 41,257.55) | 21.62 (17.31, 27.00) |
| Bhutan | 65.95 (52.30, 82.59) | 16.74 (13.31, 21.03) | 158.46 (128.30, 196.52) | 22.23 (18.11, 27.62) |
| Brunei Darussalam | 125.62 (100.39, 154.59) | 53.76 (43.58, 66.16) | 294.96 (237.09, 361.33) | 57.28 (46.33, 70.04) |
| Cambodia | 218.33 (168.39, 273.02) | 3.02 (2.34, 3.74) | 595.04 (466.52, 729.94) | 3.77 (3.01, 4.61) |
| China | 239,472.66 (198,363.11, 285,023.87) | 22.85 (18.98, 27.15) | 911,045.1 (776,346.59, 1,069,532.93) | 47.06 (40.05, 54.99) |
| Cook Islands | 0.99 (0.80, 1.19) | 6.37 (5.18, 7.65) | 1.27 (1.05, 1.55) | 6.17 (5.09, 7.51) |
| Democratic People's Republic of Korea | 3,096.55 (2,554.47, 3,713.64) | 15.93 (13.23, 19.03) | 4,964.73 (4,119.71, 5,977.53) | 15.61 (12.96, 18.71) |
| Fiji | 28.21 (22.68, 34.5) | 4.78 (3.9, 5.75) | 42.4 (34.57, 51.59) | 4.7 (3.85, 5.71) |
| Georgia | 5,099.72 (4,307.78, 6,037.32) | 84.45 (71.53, 99.72) | 4,209.88 (3,545.05, 5,059.97) | 87.97 (73.75, 104.83) |
| Guam | 7.92 (6.45, 9.47) | 6.83 (5.65, 8.11) | 11.82 (9.62, 14.18) | 6.54 (5.35, 7.82) |
| India | 133,926.94 (107,799.67, 166,434.23) | 20.68 (16.76, 25.67) | 270,718.9 (219,873.31, 332,264.31) | 20.34 (16.57, 24.97) |
| Indonesia | 5,024.62 (3,974.37, 6,141.45) | 3.37 (2.70, 4.07) | 11,410.49 (9,157.86, 13,703.69) | 4.16 (3.37, 5.00) |
| Japan | 236,120.44 (196,281.45, 281,825.31) | 151.17 (125.74, 179.69) | 408,631.58 (353,858.18, 468,152.05) | 291.9 (251.75, 336.57) |
| Kazakhstan | 11,689.39 (9,796.99, 14,063.23) | 80.02 (67.16, 96.26) | 15,822.90 (13,219.43, 18,930.39) | 81.77 (68.13, 97.46) |
| Kiribati | 2.21 (1.75, 2.71) | 4.05 (3.25, 4.96) | 3.55 (2.84, 4.38) | 3.55 (2.9, 4.32) |
| Kyrgyzstan | 2,525.82 (2,125.61, 3,007.71) | 73.48 (61.75, 87.12) | 4,283.4 (3,592.23, 5,082.54) | 73.31 (61.61, 87.09) |
| Lao People's Democratic Republic | 90.35 (69.76, 112.97) | 2.98 (2.32, 3.69) | 241.46 (190.87, 300.12) | 3.67 (2.95, 4.55) |
| Malaysia | 638.34 (532.91, 747.19) | 4.33 (3.64, 5.05) | 2,165.15 (1,879.08, 2,497.71) | 6.56 (5.71, 7.56) |
| Maldives | 5.92 (4.63, 7.29) | 4.1 (3.25, 4.98) | 27.09 (21.35, 32.97) | 4.94 (3.96, 5.96) |
| Marshall Islands | 1.15 (0.90, 1.40) | 4.22 (3.40, 5.11) | 2.09 (1.67, 2.53) | 4.10 (3.35, 4.93) |
| Mauritius | 47.08 (36.97, 58.41) | 4.55 (3.68, 5.63) | 85.52 (69.25, 102.04) | 5.5 (4.43, 6.63) |
| Micronesia (Federated States of) | 3.02 (2.40, 3.65) | 4.44 (3.61, 5.35) | 3.83 (3.10, 4.66) | 4.11 (3.40, 4.96) |
| Mongolia | 961.4 (800.51, 1,153.35) | 68.37 (56.95, 82.11) | 2,327.94 (1,906.8, 2,810.37) | 69.72 (57.44, 83.84) |
| Myanmar | 1,024.23 (796.82, 1,271.92) | 3.09 (2.42, 3.79) | 2,061.89 (1,655.90, 2,557.79) | 3.71 (3.00, 4.59) |
| Nauru | 0.36 (0.29, 0.44) | 5.33 (4.35, 6.41) | 0.41 (0.33, 0.50) | 5.02 (4.11, 6.11) |
| Nepal | 2,179.6 (1,726.32, 2,758.21) | 16.18 (12.92, 20.54) | 5,035.8 (4,048.55, 6,194.22) | 18.72 (15.04, 23.18) |
| Niue | 0.12 (0.10, 0.14) | 5.82 (4.77, 7.03) | 0.10 (0.09, 0.13) | 5.53 (4.56, 6.74) |
| Northern Mariana Islands | 2.59 (2.08, 3.15) | 6.6 (5.47, 7.98) | 3.05 (2.51, 3.7) | 5.79 (4.8, 6.96) |
| Pakistan | 9,983.78 (7,994.56, 12,369.41) | 13.75 (11.11, 17.15) | 26,815.02 (21,768.22, 33,849.02) | 16.77 (13.78, 21.25) |
| Palau | 0.74 (0.61, 0.90) | 5.62 (4.60, 6.79) | 1.18 (0.96, 1.44) | 5.22 (4.30, 6.32) |
| Papua New Guinea | 115.95 (91.86, 140.48) | 4.05 (3.24, 4.88) | 284.82 (229.11, 349.71) | 3.65 (2.96, 4.45) |
| Philippines | 2,067.23 (1,629.7, 2,519.00) | 4.47 (3.59, 5.38) | 5,072.91 (4,044.20, 6,165.55) | 4.96 (4.02, 6.00) |
| Republic of Korea | 17,167.24 (15,173.62, 19,498.03) | 37.89 (33.49, 42.88) | 46,956.28 (44,197.19, 49,996.79) | 70.21 (66.03, 74.79) |
| Samoa | 6.34 (5.20, 7.63) | 5.60 (4.60, 6.71) | 8.53 (7.00, 10.32) | 4.77 (3.92, 5.78) |
| Seychelles | 2.97 (2.37, 3.64) | 4.66 (3.75, 5.71) | 6.46 (5.25, 7.78) | 5.45 (4.43, 6.55) |
| Singapore | 1,615.15 (1,304.65, 1,933.67) | 47.14 (38.30, 56.56) | 3,179.3 (2,592.63, 3,962.20) | 41.88 (34.28, 51.91) |
| Solomon Islands | 7.51 (5.96, 9.23) | 3.49 (2.80, 4.25) | 16.82 (13.30, 20.94) | 3.35 (2.73, 4.12) |
| Sri Lanka | 908.78 (733.68, 1,119.1) | 5.98 (4.90, 7.28) | 2,295.04 (2,002.61, 2,636.90) | 9.51 (8.29, 10.90) |
| Taiwan (Province of China) | 2,220.90 (1,922.73, 2,556.93) | 11.23 (9.74, 12.86) | 9,063.90 (8,139.25, 10,153.03) | 27.8 (24.8, 31.28) |
| Tajikistan | 2,391.29 (1,972.31, 2,877.31) | 68.84 (56.59, 83.10) | 5,518.57 (4,622.75, 6,631.82) | 71.19 (59.54, 85.49) |
| Thailand | 1,733.83 (1,327.3, 2,180.04) | 3.31 (2.57, 4.15) | 3,808.42 (3,078.37, 4,629.82) | 4.28 (3.44, 5.22) |
| Timor-Leste | 18.47 (14.21, 22.97) | 3.2 (2.51, 3.91) | 41.04 (32.53, 49.90) | 3.9 (3.14, 4.72) |
| Tokelau | 0.06 (0.05, 0.07) | 4.65 (3.78, 5.66) | 0.07 (0.06, 0.09) | 5.36 (4.37, 6.54) |
| Tonga | 3.82 (3.13, 4.61) | 5.55 (4.56, 6.66) | 4.37 (3.57, 5.29) | 4.96 (4.08, 5.98) |
| Turkmenistan | 1,757.87 (1,462.97, 2,085.24) | 69.03 (57.31, 82.24) | 3,805.52 (3,154.54, 4,550.68) | 77.89 (64.42, 92.76) |
| Tuvalu | 0.36 (0.29, 0.44) | 4.48 (3.63, 5.44) | 0.46 (0.38, 0.55) | 4.08 (3.38, 4.88) |
| Uzbekistan | 10,504.66 (8,846.78, 12,563.04) | 74.25 (62.3, 88.98) | 23,340.11 (19,451.96, 27,951.89) | 75.79 (63.42, 90.5) |
| Vanuatu | 4.3 (3.37, 5.25) | 4.27 (3.44, 5.21) | 9.26 (7.49, 11.32) | 3.94 (3.23, 4.77) |
| Viet Nam | 4,754.49 (3,636.33, 5,967.47) | 9.12 (7.04, 11.43) | 20,278.1 (16,049.66, 25,062.95) | 18.35 (14.68, 22.55) |

Appendix table 3. IBD deaths in 1990 and 2019 for both sexes in age-standardised rates by location in Asia.

| Location | 1990 | | 2019 | |
| --- | --- | --- | --- | --- |
|  | Counts (95% UI) | Rate (95% UI) | Counts (95% UI) | Rate (95% UI) |
| Asia | 12,029.57 (8,669.15, 15,526.27) | 0.67 (0.46, 0.90) | 13,957.38 (11,897.93, 16,020.75) | 0.33 (0.28, 0.37) |
| East Asia | 5,734.25 (4,169.43, 7,373.88) | 0.86 (0.59, 1.14) | 4,982.78 (4,074.27, 5,769.1) | 0.31 (0.25, 0.35) |
| South Asia | 3,863.53 (2,533.48, 5,504.05) | 0.69 (0.47, 1.06) | 5,701.83 (4,286.07, 7,409.52) | 0.44 (0.33, 0.57) |
| Southeast Asia | 1,229.97 (741.27, 1,536.19) | 0.50 (0.27, 0.66) | 1,497.98 (1,139.48, 1,771.09) | 0.28 (0.21, 0.33) |
| Central Asia | 215.32 (166.61, 250.72) | 0.39 (0.30, 0.46) | 249.12 (218.34, 287.6) | 0.32 (0.28, 0.37) |
| High-income Asia Pacific | 684.99 (439.29, 788.96) | 0.40 (0.24, 0.47) | 694.14 (555.53, 912.49) | 0.13 (0.11, 0.18) |
| American Samoa | 0.38 (0.22, 0.54) | 1.54 (0.89, 2.28) | 0.24 (0.17, 0.41) | 0.53 (0.37, 0.90) |
| Armenia | 7.15 (5.40, 8.60) | 0.25 (0.19, 0.30) | 10.82 (8.15, 13.19) | 0.28 (0.21, 0.34) |
| Azerbaijan | 11.61 (8.64, 14.67) | 0.22 (0.16, 0.28) | 13.04 (9.62, 17.93) | 0.16 (0.11, 0.23) |
| Bangladesh | 544.42 (333.31, 804.71) | 1.00 (0.64, 1.64) | 712.65 (431.02, 1,068.27) | 0.56 (0.35, 0.83) |
| Bhutan | 1.84 (0.84, 3.98) | 0.68 (0.35, 1.53) | 2.88 (1.75, 6.60) | 0.55 (0.34, 1.23) |
| Brunei Darussalam | 1.13 (0.79, 1.63) | 1.52 (1.08, 2.01) | 2.57 (2.10, 3.24) | 1.48 (0.97, 1.89) |
| Cambodia | 25.79 (13.85, 39.38) | 0.50 (0.27, 0.71) | 37.76 (26.12, 47.73) | 0.35 (0.23, 0.45) |
| China | 5,559.54 (4,047.82, 7,202.71) | 0.86 (0.59, 1.16) | 4,675.97 (3,774.48, 5,461.44) | 0.30 (0.24, 0.35) |
| Cook Islands | 0.21 (0.15, 0.29) | 1.64 (1.17, 2.20) | 0.18 (0.12, 0.25) | 0.81 (0.53, 1.11) |
| Democratic People's Republic of Korea | 73.86 (40.78, 114.07) | 0.57 (0.33, 0.82) | 107.66 (74.79, 147.23) | 0.41 (0.28, 0.59) |
| Fiji | 1.39 (1.06, 1.81) | 0.35 (0.26, 0.45) | 1.78 (1.29, 2.38) | 0.26 (0.19, 0.34) |
| Georgia | 16.47 (10.09, 22.38) | 0.29 (0.18, 0.39) | 17.03 (10.48, 21.75) | 0.29 (0.19, 0.37) |
| Guam | 0.23 (0.18, 0.29) | 0.33 (0.26, 0.42) | 0.28 (0.20, 0.40) | 0.16 (0.12, 0.23) |
| India | 2,770.18 (1,782.59, 3,801.70) | 0.63 (0.43, 0.91) | 4,214.16 (2,950.21, 5,530.80) | 0.40 (0.29, 0.53) |
| Indonesia | 704.33 (401.48, 910.44) | 0.77 (0.37, 1.04) | 912.65 (582.35, 1,179.30) | 0.55 (0.33, 0.70) |
| Japan | 366.91 (270.32, 393.60) | 0.25 (0.18, 0.27) | 452.47 (350.1, 629.43) | 0.11 (0.09, 0.16) |
| Kazakhstan | 75.83 (55.84, 95.02) | 0.57 (0.41, 0.72) | 81.53 (65.76, 100.00) | 0.46 (0.37, 0.56) |
| Kiribati | 0.70 (0.32, 1.20) | 1.81 (0.99, 2.71) | 0.73 (0.41, 1.28) | 1.08 (0.68, 1.67) |
| Kyrgyzstan | 15.26 (9.93, 18.52) | 0.42 (0.29, 0.50) | 9.75 (8.08, 12.45) | 0.19 (0.16, 0.25) |
| Lao People's Democratic Republic | 9.78 (4.79, 14.73) | 0.45 (0.22, 0.72) | 10.64 (5.91, 17.48) | 0.26 (0.14, 0.41) |
| Malaysia | 16.09 (12.94, 19.91) | 0.18 (0.14, 0.22) | 41.44 (29.77, 55.84) | 0.17 (0.12, 0.23) |
| Maldives | 0.44 (0.26, 0.71) | 0.53 (0.32, 0.81) | 0.87 (0.67, 1.14) | 0.30 (0.23, 0.40) |
| Marshall Islands | 0.25 (0.15, 0.38) | 1.32 (0.84, 1.88) | 0.29 (0.16, 0.47) | 0.83 (0.50, 1.31) |
| Mauritius | 1.26 (1.1, 1.5) | 0.17 (0.14, 0.19) | 3.47 (2.61, 4.40) | 0.22 (0.17, 0.28) |
| Micronesia (Federated States of) | 0.68 (0.43, 1.08) | 1.36 (0.88, 1.97) | 0.53 (0.28, 0.95) | 0.80 (0.48, 1.32) |
| Mongolia | 10.71 (5.98, 15.47) | 0.73 (0.47, 1.01) | 11.93 (8.54, 16.42) | 0.45 (0.33, 0.61) |
| Myanmar | 59.71 (33.89, 94.72) | 0.22 (0.13, 0.31) | 59.3 (44.17, 81.61) | 0.13 (0.10, 0.17) |
| Nauru | 0.07 (0.03, 0.12) | 1.37 (0.82, 2.13) | 0.04 (0.02, 0.08) | 0.92 (0.54, 1.45) |
| Nepal | 93.29 (56.49, 165.03) | 0.98 (0.57, 1.86) | 114.84 (71.35, 190.36) | 0.59 (0.36, 0.98) |
| Niue | 0.02 (0.02, 0.03) | 0.96 (0.67, 1.38) | 0.01 (0.01, 0.02) | 0.63 (0.38, 0.91) |
| Northern Mariana Islands | 0.28 (0.18, 0.42) | 1.28 (0.85, 1.82) | 0.15 (0.10, 0.29) | 0.38 (0.26, 0.73) |
| Pakistan | 453.8 (240.40, 865.61) | 0.78 (0.39, 1.57) | 657.29 (446.77, 993.28) | 0.62 (0.41, 0.99) |
| Palau | 0.09 (0.05, 0.16) | 0.88 (0.53, 1.46) | 0.11 (0.05, 0.17) | 0.62 (0.29, 0.97) |
| Papua New Guinea | 12.92 (7.28, 20.07) | 0.54 (0.31, 0.80) | 25.31 (16.28, 38.24) | 0.42 (0.27, 0.63) |
| Philippines | 98.16 (53.74, 124.8) | 0.35 (0.16, 0.46) | 117.38 (95.03, 154.87) | 0.16 (0.13, 0.20) |
| Republic of Korea | 313.12 (143.27, 399.24) | 1.76 (0.56, 2.34) | 233.74 (182.56, 313.27) | 0.30 (0.23, 0.39) |
| Samoa | 0.86 (0.57, 1.26) | 0.98 (0.66, 1.49) | 0.92 (0.65, 1.27) | 0.63 (0.45, 0.85) |
| Seychelles | 0.17 (0.11, 0.22) | 0.30 (0.19, 0.38) | 0.22 (0.16, 0.29) | 0.22 (0.16, 0.30) |
| Singapore | 3.83 (2.99, 4.31) | 0.18 (0.14, 0.20) | 5.36 (4.24, 8.21) | 0.07 (0.06, 0.11) |
| Solomon Islands | 1.18 (0.59, 2.11) | 0.75 (0.42, 1.16) | 1.94 (1.12, 3.35) | 0.53 (0.33, 0.79) |
| Sri Lanka | 18.86 (14.26, 23.82) | 0.18 (0.14, 0.23) | 21.50 (15.23, 29.60) | 0.10 (0.07, 0.13) |
| Taiwan (Province of China) | 100.85 (66.95, 113.45) | 0.91 (0.57, 1.04) | 199.14 (152.47, 263.62) | 0.50 (0.38, 0.65) |
| Tajikistan | 20.60 (11.00, 33.72) | 0.41 (0.28, 0.58) | 18.29 (12.73, 25.72) | 0.30 (0.22, 0.41) |
| Thailand | 71.76 (51.56, 94.8) | 0.20 (0.14, 0.26) | 110.55 (79.96, 149.21) | 0.12 (0.08, 0.15) |
| Timor-Leste | 1.33 (0.61, 2.57) | 0.42 (0.17, 0.83) | 2.75 (1.52, 4.75) | 0.36 (0.20, 0.61) |
| Tokelau | 0.01 (0.01, 0.02) | 0.89 (0.57, 1.34) | 0.01 (0.01, 0.01) | 0.58 (0.42, 0.78) |
| Tonga | 0.62 (0.38, 0.88) | 1.14 (0.67, 1.64) | 0.62 (0.46, 0.83) | 0.76 (0.56, 1.02) |
| Turkmenistan | 9.76 (6.50, 13.36) | 0.37 (0.28, 0.49) | 14.18 (9.25, 19.89) | 0.33 (0.22, 0.46) |
| Tuvalu | 0.07 (0.04, 0.11) | 1.08 (0.65, 1.59) | 0.07 (0.04, 0.10) | 0.71 (0.47, 1.05) |
| Uzbekistan | 47.93 (31.48, 59.18) | 0.28 (0.20, 0.39) | 72.55 (58.49, 92.78) | 0.29 (0.23, 0.39) |
| Vanuatu | 0.85 (0.47, 1.34) | 1.22 (0.69, 2.03) | 1.52 (0.92, 2.29) | 0.84 (0.52, 1.24) |
| Viet Nam | 220.67 (119.52, 326.60) | 0.58 (0.30, 0.88) | 177.48 (118.78, 246.95) | 0.22 (0.15, 0.32) |

Appendix table 4. IBD DALY in 1990 and 2019 for both sexes in age-standardised rates by location in Asia.

| Location | 1990 | | 2019 | |
| --- | --- | --- | --- | --- |
|  | Counts (95% UI) | Rate (95% UI) | Counts (95% UI) | Rate (95% UI) |
| Asia | 540,948.59 (382,320.39, 666,324.37) | 21.08 (15.82, 25.98) | 649,760.15 (530,394.6, 783,180.56) | 13.51 (11.08, 16.21) |
| East Asia | 240,231 (167,509.95, 299,890.51) | 24.23 (17.74, 29.83) | 239,962.98 (187,122.67, 299,808.1) | 13.08 (10.31, 16.19) |
| South Asia | 171,447.62 (105,178.05, 227,334.12) | 21.48 (14.77, 29.47) | 221,977.56 (166,881.48, 278,494.47) | 14,00 (10.70, 17.52) |
| Southeast Asia | 45.316.22 (30,761.95, 56,838.41) | 13.16 (8.47, 15.90) | 46,685.83 (38,846.83, 55,186.44) | 7.45 (6.06, 8.76) |
| Central Asia | 15,951.88 (12, 800.38, 19,230.11) | 26.11 (21.03, 31.71) | 20,236.62 (16,356.51, 24,805.81) | 22.26 (18.04, 27.31) |
| High-income Asia Pacific | 51,699.43 (38,590.91, 66,562.64) | 26.43 (19.87, 33.86) | 78,601.43 (55,261.20, 105,049.27) | 34.55 (23.75, 46.76) |
| American Samoa | 14.77 (9.03, 21.29) | 44.49 (26.51, 63.51) | 7.77 (5.43, 13.67) | 15.67 (10.89, 28.11) |
| Armenia | 637.47 (491.59, 805.15) | 19.99 (15.55, 25.21) | 764.07 (592.93, 979.30) | 20.50 (15.81, 26.35) |
| Azerbaijan | 1,079.50 (815.45, 1,410.44) | 17.65 (13.27, 23.02) | 1,829.39 (1,288.83, 2,470.07) | 16.20 (11.65, 21.65) |
| Bangladesh | 23,780.62 (13,440.34, 35,324.16) | 31.40 (20.47, 45.16) | 26,353.91 (17,362.99, 37,248.03) | 18.10 (12.11, 25.27) |
| Bhutan | 85.59 (38.02, 159.70) | 21.23 (11.26, 43.14) | 101.77 (66.65, 201.93) | 15.82 (10.39, 31.52) |
| Brunei Darussalam | 57.09 (40.19, 84.88) | 36.51 (27.84, 47.59) | 113.86 (88.58, 146.98) | 32.76 (26.62, 39.71) |
| Cambodia | 1,175.08 (546.78, 2,166.50) | 14.51 (8.27, 21.45) | 1,221.73 (890.47, 1,546.25) | 8.98 (6.42, 11.33) |
| China | 234,245.17 (162,915.80, 293,663.24) | 24.47 (17.88, 30.19) | 232,463.85 (179,902.7, 291,090.42) | 13.1 (10.29, 16.31) |
| Cook Islands | 7.66 (5.26, 11.13) | 50.74 (35.37, 72.08) | 5.08 (3.26, 7.14) | 24.94 (15.58, 36.18) |
| Democratic People's Republic of Korea | 3,362.72 (1,696.82, 6,032.75) | 17.24 (9.70, 27.63) | 3,161.37 (2,322.5, 4,117.39) | 11.10 (8.17, 14.58) |
| Fiji | 59.47 (45.13, 79.91) | 10.67 (8.31, 13.7) | 68.20 (49.39, 91.15) | 8.17 (5.94, 10.77) |
| Georgia | 1,222.29 (911.29, 1,581.49) | 20.37 (15.24, 26.41) | 990.53 (741.28, 1269.24) | 20.25 (15.35, 26.28) |
| Guam | 9.13 (7.08, 12.22) | 9.01 (7.19, 11.32) | 9.40 (7.17, 13.32) | 5.39 (4.11, 7.68) |
| India | 127,567.73 (77,797.7, 169,842.98) | 20.11 (13.58, 26.72) | 164,416.35 (117,527.18, 208,276.12) | 13.04 (9.44, 16.50) |
| Indonesia | 26,026.06 (16,964.58, 34,444.86) | 19.48 (11.7, 24.6) | 24,942.78 (18,267.77, 32,251.58) | 11.66 (8.16, 14.98) |
| Japan | 41,769.34 (30,061.52, 56,026.48) | 27.14 (19.46, 36.26) | 67,423.73 (46,877.43, 91,494.79) | 46.58 (31.41, 63.54) |
| Kazakhstan | 4,457.76 (3,567.05, 5,515.47) | 30.23 (24.06, 37.42) | 5,172.4 (4,164.37, 6,466.26) | 26.97 (21.78, 33.61) |
| Kiribati | 28.43 (10.54, 57.96) | 53.42 (24.47, 92.59) | 29.59 (14.16, 54.87) | 31.83 (17.58, 55.40) |
| Kyrgyzstan | 1,109.15 (783.96, 1,375.87) | 28.00 (21.04, 34.06) | 1,030.99 (788.97, 1,325.19) | 17.37 (13.34, 22.37) |
| Lao People's Democratic Republic | 385.01 (176.94, 638.83) | 13.04 (6.62, 19.19) | 386.94 (233.61, 610.44) | 6.97 (4.13, 11.24) |
| Malaysia | 612.35 (503.48, 742.17) | 5.01 (4.15, 6.1) | 1,434.47 (1,090.08, 1,813.92) | 4.92 (3.71, 6.26) |
| Maldives | 18.52 (10.38, 33.46) | 13.21 (8.16, 20.38) | 29.28 (22.85, 38.33) | 7.20 (5.69, 9.23) |
| Marshall Islands | 10.2 (5.75, 16.89) | 38.63 (23.47, 58.16) | 11.78 (6.38, 20.44) | 24.94 (14.63, 40.72) |
| Mauritius | 49.81 (43.28, 60.51) | 5.3 (4.63, 6.29) | 116.63 (89.84, 146.68) | 7.70 (5.86, 9.69) |
| Micronesia (Federated States of) | 27.63 (15.65, 49.27) | 40.59 (25.23, 65.57) | 20.11 (9.84, 38.13) | 23.88 (12.56, 43.11) |
| Mongolia | 690.26 (380.30, 1073.22) | 38.19 (25.22, 51.55) | 813.99 (620.32, 1027.98) | 24.75 (19.13, 31.11) |
| Myanmar | 2,819.97 (1,386.27, 5,127.72) | 7.97 (4.53, 12.88) | 2,257.54 (1,680.06, 3,214.98) | 4.34 (3.28, 6.04) |
| Nauru | 3.22 (1.42, 6.46) | 42.03 (22.9, 73.19) | 2.12 (0.91, 4.1) | 27.18 (13.97, 47.82) |
| Nepal | 3,779.64 (2,182.8, 5,902.97) | 28.52 (18.28, 47.84) | 3,819.28 (2,682.59, 5781.4) | 15.65 (10.93, 23.88) |
| Niue | 0.65 (0.43, 1.00) | 29.80 (19.61, 46.61) | 0.35 (0.20, 0.52) | 19.00 (10.29, 29.66) |
| Northern Mariana Islands | 12.07 (7.64, 18.79) | 34.73 (23.54, 50.03) | 5.13 (3.57, 9.45) | 11.56 (7.82, 20.63) |
| Pakistan | 16,234.03 (9,791.17, 26,747.75) | 21.52 (12.69, 38.34) | 27,286.26 (18,727.07, 38,378.55) | 17.80 (12.91, 25.37) |
| Palau | 3.35 (1.55, 6.59) | 26.21 (13.51, 47.84) | 3.85 (1.63, 6.48) | 19.34 (8.32, 32.27) |
| Papua New Guinea | 660.79 (335.45, 1,139.91) | 19.34 (11.20, 29.45) | 1,258.15 (757.75, 2,033.71) | 14.68 (9.50, 21.87) |
| Philippines | 3,743.12 (2,419.05, 4,550.77) | 8.94 (5.36, 11.07) | 4,555.8 (3,797.20, 5,980.64) | 4.87 (4.06, 6.26) |
| Republic of Korea | 9,497.96 (6,903.37, 11,453.94) | 31.72 (18.30, 38.85) | 10,451.4 (7,828.58, 13,342.91) | 15.00 (11.13, 19.33) |
| Samoa | 30.66 (19.67, 45.85) | 27.95 (18.61, 40.58) | 30.33 (19.36, 45.04) | 17.97 (11.92, 26.18) |
| Seychelles | 5.22 (3.51, 6.68) | 8.38 (5.69, 10.68) | 6.87 (5.27, 8.61) | 6.28 (4.77, 7.85) |
| Singapore | 375.05 (278.76, 490.17) | 12.19 (9.33, 15.58) | 612.43 (427.39, 838.33) | 8.16 (5.69, 11.25) |
| Solomon Islands | 52.56 (23.61, 101.13) | 24.39 (12.55, 43.26) | 85.81 (47.75, 156.26) | 17.28 (10.22, 29.48) |
| Sri Lanka | 812.99 (609.35, 1,032.24) | 5.73 (4.38, 7.09) | 895.19 (683.78, 1,147.11) | 3.77 (2.86, 4.79) |
| Taiwan (Province of China) | 2,623.11 (1,919.12, 2,947.09) | 17.35 (12.32, 19.43) | 4,337.77 (3,498.97, 5,415.83) | 12.4 (10.06, 15.44) |
| Tajikistan | 1,691.06 (852.52, 2,831.45) | 30.29 (19.52, 43.28) | 1,646.14 (1,219.49, 2,185.01) | 20.6 (15.55, 26.9) |
| Thailand | 2,898.7 (2,029.16, 3,945.77) | 6.06 (4.39, 7.89) | 3,271.7 (2,452.85, 4,259.28) | 3.64 (2.76, 4.68) |
| Timor-Leste | 58.66 (28.76, 102.28) | 11.64 (5.58, 22.42) | 86.35 (47.88, 138.05) | 9.14 (5.17, 14.89) |
| Tokelau | 0.36 (0.21, 0.54) | 25.84 (15.77, 38.77) | 0.21 (0.15, 0.31) | 16.11 (11.19, 22.98) |
| Tonga | 21.23 (13.79, 29.93) | 31.30 (19.82, 44.12) | 18.84 (13.42, 26.67) | 21.58 (15.55, 30.10) |
| Turkmenistan | 765.20 (493.23, 1049.00) | 24.59 (17.78, 31.67) | 1,120.13 (804.65, 1,438.07) | 22.90 (16.63, 29.27) |
| Tuvalu | 2.72 (1.34, 4.83) | 33.30 (17.86, 54.06) | 2.27 (1.40, 3.61) | 21.17 (13.37, 33.22) |
| Uzbekistan | 4,299.20 (3,005.54, 5,336.50) | 23.48 (17.64, 29.48) | 6,868.98 (5,390.98, 8,500.41) | 21.90 (17.37, 27.16) |
| Vanuatu | 33.38 (16.64, 55.06) | 34.45 (18.68, 54.26) | 57.27 (32.81, 92.36) | 25.36 (15.29, 38.79) |
| Viet Nam | 6,650.49 (4,129.36, 8,927.91) | 13.87 (8.53, 18.99) | 7,419.38 (5,509.58, 9,603.55) | 7.60 (5.65, 9.80) |

Appendix table 5. Joinpoint regression analysis of ASIR, ASPR, ASDR and ASMR for IBD in Asia from 1990 to 2019.

| Gender | ASIR | | | ASPR | | | ASMR | | |
| --- | --- | --- | --- | --- | --- | --- | --- | --- | --- |
|  | Period | APC (95% CI) | AAPC (95% CI) | Period | APC (95% CI) | AAPC (95% CI) | Period | APC (95% CI) | AAPC (95% CI) |
| Both | 1990-1996 | 1.3 (1.2, 1.4) | 0.8 (0.7, 0.9) | 1990-1993 | 2.6 (2.1, 3.1) | 0.9 (0.8, 1.1) | 1990-1999 | -1.5 (-1.6, -1.5) | -2.5 (-2.5, -2.4) |
|  | 1996-1999 | 2.1 (1.6, 2.7) |  | 1993-1996 | 1.5 (0.6, 2.4) |  | 1999-2004 | -2.5 (-2.7, -2.3) |  |
|  | 1999-2005 | 0.3 (0.2, 0.4) |  | 1996-1999 | 3.9 (3, 4.7) |  | 2004-2009 | -4.5 (-4.7, -4.4) |  |
|  | 2005-2014 | 0.8 (0.7, 0.9) |  | 1999-2002 | 0.8 (0, 1.6) |  | 2009-2013 | -3.7 (-3.9, -3.5) |  |
|  | 2014-2017 | -0.6 (-1.2, -0.1) | | 2002-2013 | 0.3 (0.3, 0.4) |  | 2013-2016 | -2.1 (-2.5, -1.7) |  |
|  | 2017-2019 | 0.8 (0.3, 1.4) |  | 2013-2019 | -0.4 (-0.5, -0.2) |  | 2016-2019 | -0.5 (-0.7, -0.3) |  |
| Female | 1990-1996 | 1.2 (1.1, 1.2) | 0.9 (0.9, 0.9） | 1990-1992 | 2.6 (1.6, 3.7) | 1.0 (0.8, 1.1) | 1990-2000 | -1.7 (-1.8, -1.6) | -2.7 (-2.8, -2.6) |
|  | 1996-1999 | 2.6 (2.3, 2.8) |  | 1992-1996 | 1.6 (1.1, 2.1) |  | 2000-2004 | -2.5 (-3.0, -2.1) |  |
|  | 1999-2006 | 0.5 (0.5, 0.6) |  | 1996-1999 | 3.8 (2.9, 4.8) |  | 2004-2010 | -5.3 (-5.5, -5.1) |  |
|  | 2006-2014 | 0.9 (0.9, 1.0) |  | 1999-2002 | 0.9 (0.0, 1.7) |  | 2010-2013 | -4.0 (-4.5-3.4) |  |
|  | 2014-2017 | -0.5 (-0.8, -0.3) | | 2002-2014 | 0.4 (0.4, 0.5) |  | 2013-2016 | -2.2 (-2.8, -1.7) |  |
|  | 2017-2019 | 0.9 (0.6, 1.2) |  | 2014-2019 | -0.4 (-0.6, -0.2) |  | 2016-2019 | -0.1 (-0.4, 0.2) |  |
| Male | 1990-2000 | 1.5 (1.4, 1.5) | 0.7 (0.6, 0.9) | 1990-1993 | 2.7 (2.3, 3.2) | 0.9 (0.8, 1.1) | 1990-1997 | -1.2 (-1.4, -1.0) | -2.2 (-2.3, -2.1) |
|  | 2000-2005 | 0.1 (-0.2, 0.4) |  | 1993-1996 | 1.6 (0.7, 2.5) |  | 1997-2002 | -2.1 (-2.5, -1.8) |  |
|  | 2005-2014 | 0.7 (0.6, 0.8) |  | 1996-1999 | 3.9 (3.1, 4.7) |  | 2002-2013 | -3.3 (-3.3, -3.2) |  |
|  | 2014-2017 | -0.8 (-1.6, 0.1) | | 1999-2002 | 0.8 (0.1, 1.6) |  | 2013-2016 | -1.9 (-2.5, -1.3) |  |
|  | 2017-2019 | 0.8 (-0.1, 1.7) |  | 2002-2013 | 0.2 (0.2, 0.3) |  | 2016-2019 | -0.9 (-1.3, -0.6) |  |
|  |  |  |  | 2013-2019 | -0.4 (-0.6, -0.3) |  |  |  |  |

AAPC, average annual percent change presented for the whole period; APC, annual percent change; CI, confidence interval.

Appendix table 6. Long age of APC analysis across to different regions.

| LongAge | Asia | | | | East Asia | | | Southeast Asia | | | South Asia | | | Central Asia | | | High-income | | |
| --- | --- | --- | --- | --- | --- | --- | --- | --- | --- | --- | --- | --- | --- | --- | --- | --- | --- | --- | --- |
|  | Age | Both | Male | Female | Both | Male | Female | Both | Male | Female | Both | Male | Female | Both | Male | Female | Both | Male | Female |
|  |  | Rate(95%CI) | Rate(95%CI) | Rate(95%CI) | Rate(95%CI) | Rate(95%CI) | Rate(95%CI) | Rate(95%CI) | Rate(95%CI) | Rate(95%CI) | Rate(95%CI) | Rate(95%CI) | Rate(95%CI) | Rate(95%CI) | Rate(95%CI) | Rate(95%CI) | Rate(95%CI) | Rate(95%CI) | Rate(95%CI) |
|  | 0~4  year | 0.02 (0.01, 0.04) | 0.02 (0.01, 0.04) | 0.01 (0.01, 0.03) | 0.01 (0.01, 0.01) | 0.01 (0.00, 0.01) | 0.01 (0.00, 0.01) | 0.01 (0.01, 0.01) | 0.01 (0.01, 0.01) | 0.01 (0.00, 0.01) | 0.02 (0.01, 0.03) | 0.02 (0.01, 0.03) | 0.02 (0.01, 0.02) | 0.04 (0.02, 0.07) | 0.03 (0.01, 0.07) | 0.03 (0.01, 0.07) | 0.03 (0.00, 1.78) | 0.04 (0.00, 2.18) | 0.03 (0.00, 1.41) |
|  | 5~9  year | 0.26 (0.21, 0.31) | 0.27 (0.21, 0.34) | 0.24 (0.20, 0.29) | 0.14 (0.13, 0.15) | 0.15 (0.14, 0.17) | 0.12 (0.11, 0.14) | 0.11 (0.10, 0.12) | 0.12 (0.11, 0.14) | 0.10 (0.09, 0.12) | 0.27 (0.24, 0.29) | 0.28 (0.26, 0.31) | 0.25 (0.23, 0.27) | 0.66 (0.58, 0.74) | 0.66 (0.56, 0.78) | 0.64 (0.54, 0.77) | 0.85 (0.41, 1.74) | 0.86 (0.40, 1.85) | 0.85 (0.44, 1.66) |
|  | 10~14  year | 1.21 (1.10, 1.33) | 1.31 (1.17, 1.46) | 1.10 (1.01, 1.20) | 0.69 (0.66, 0.72) | 0.78 (0.74, 0.82) | 0.59 (0.56, 0.62) | 0.42 (0.40, 0.44) | 0.46 (0.42, 0.49) | 0.39 (0.36, 0.42) | 1.04 (0.99, 1.10) | 1.13 (1.07, 1.2) | 0.95 (0.91, 0.99) | 3.33 (3.15, 3.53) | 3.37 (3.11, 3.65) | 3.28 (3.02, 3.56) | 5.89 (4.44, 7.82) | 6.28 (4.67, 8.44) | 5.56 (4.26, 7.25) |
|  | 15~19  year | 4.50 (4.25, 4.76) | 4.49 (4.20, 4.81) | 4.49 (4.28, 4.71) | 2.54 (2.48, 2.60) | 2.60 (2.52, 2.69) | 2.46 (2.38, 2.54) | 1.21 (1.16, 1.25) | 1.13 (1.07, 1.19) | 1.28 (1.22, 1.35) | 3.22 (3.12, 3.33) | 3.14 (3.02, 3.26) | 3.31 (3.22, 3.41) | 12.39 (11.97, 12.82) | 11.06 (10.52, 11.62) | 13.75 (13.12, 14.4) | 29.15 (25.39, 33.48) | 30.51 (26.35, 35.32) | 28.15 (24.76, 32.01) |
|  | 20~24  year | 11.62 (11.16, 12.10) | 12.19 (11.63, 12.78) | 11.01 (10.63, 11.39) | 6.26 (6.16, 6.37) | 6.76 (6.61, 6.91) | 5.74 (5.61, 5.87) | 2.47 (2.40, 2.54) | 2.27 (2.17, 2.37) | 2.66 (2.55, 2.76) | 7.14 (6.97, 7.31) | 7.08 (6.88, 7.29) | 7.20 (7.04, 7.35) | 29.43 (28.7, 30.18) | 26.7 (25.73, 27.70) | 32.14 (31.05, 33.26) | 92.39 (84.43, 101.11) | 100.76 (91.7, 110.72) | 84.25 (77.39, 91.73) |
|  | 25~29  year | 22.97 (22.23, 23.74) | 24.64 (23.72, 25.58) | 21.22 (20.63, 21.82) | 12.79 (12.62, 12.95) | 13.85 (13.61, 14.10) | 11.66 (11.45, 11.87) | 4.24 (4.14, 4.35) | 3.84 (3.70, 3.99) | 4.63 (4.48, 4.79) | 13.36 (13.10, 13.63) | 13.39 (13.08, 13.71) | 13.32 (13.09, 13.56) | 54.48 (53.38, 55.61) | 50.32 (48.83, 51.86) | 58.56 (56.94, 60.24) | 188.47 (175.32, 202.6) | 215.17 (199.58, 231.98) | 161.4 (150.69, 172.87) |
|  | 30~34  year | 36.87 (35.84, 37.93) | 39.65 (38.37, 40.96) | 33.92 (33.09, 34.76) | 22.17 (21.92, 22.42) | 24.18 (23.82, 24.55) | 20.06 (19.75, 20.37) | 6.30 (6.16, 6.45) | 5.61 (5.42, 5.81) | 6.97 (6.76, 7.19) | 21.96 (21.59, 22.34) | 22.02 (21.59, 22.47) | 21.88 (21.55, 22.22) | 84.15 (82.66, 85.66) | 78.67 (76.65, 80.75) | 89.5 (87.33, 91.73) | 282.89 (265.4, 301.53) | 326.11 (305.17, 348.49) | 238.64 (224.62, 253.54) |
|  | 35~39  year | 52.56 (51.28, 53.86) | 54.84 (53.3, 56.41) | 50.06 (49.01, 51.12) | 36.32 (35.97, 36.66) | 37.51 (37.03, 37.99) | 34.90 (34.44, 35.36) | 8.46 (8.29, 8.64) | 7.38 (7.15, 7.61) | 9.53 (9.27, 9.80) | 32.21 (31.74, 32.68) | 32.07 (31.53, 32.62) | 32.32 (31.90, 32.75) | 115.66 (113.91, 117.44) | 107.94 (105.55, 110.37) | 123.13 (120.57, 125.74) | 353.31 (334.09, 373.64) | 402.76 (379.9, 427.00) | 303.01 (287.44, 319.41) |
|  | 40~44  year | 65.22 (63.76, 66.71) | 69.31 (67.54, 71.13) | 60.81 (59.62, 62.01) | 50.19 (49.76, 50.62) | 52.88 (52.27, 53.49) | 47.24 (46.68, 47.80) | 10.48 (10.28, 10.69) | 8.92 (8.66, 9.19) | 12.02 (11.72, 12.34) | 42.62 (42.06, 43.19) | 42.02 (41.37, 42.68) | 43.24 (42.72, 43.77) | 144.01 (141.95, 146.11) | 134.5 (131.70, 137.37) | 153.07 (150.07, 156.12) | 367.69 (349.00, 387.37) | 441.05 (417.9, 465.48) | 292.04 (277.8, 307.01) |
|  | 45~49  year | 76.55 (74.91, 78.23) | 82.67 (80.64, 84.74) | 70.06 (68.74, 71.40) | 64.05 (63.54, 64.56) | 70.15 (69.41, 70.90) | 57.65 (57.01, 58.30) | 11.48 (11.26, 11.7) | 10.14 (9.86, 10.44) | 12.79 (12.47, 13.12) | 50.3 (49.65, 50.95) | 50.64 (49.89, 51.41) | 49.9 (49.31, 50.50) | 150.83 (148.67, 153.03) | 158.28 (155.05, 161.58) | 143.89 (140.99, 146.84) | 379.89 (360.88, 399.90) | 443.71 (420.62, 468.07) | 314.91 (299.97, 330.60) |
|  | 50~54  year | 85.10(83.26, 86.99) | 92.97 (90.67, 95.33) | 76.88 (75.42, 78.37) | 80.99 (80.36, 81.62) | 88.94 (88.02, 89.87) | 72.71 (71.92, 73.51) | 11.86 (11.63, 12.1) | 11.11 (10.79, 11.44) | 12.60 (12.27, 12.94) | 51.96 (51.27, 52.66) | 56.39 (55.54, 57.26) | 47.15 (46.56, 47.74) | 169.93 (167.50, 172.39) | 179.6 (175.95, 183.32) | 161.21 (157.99, 164.50) | 358.21 (339.54, 377.92) | 408.05 (385.73, 431.67) | 308.04 (292.99, 323.87) |
|  | 55~59  year | 90.31 (88.25, 92.42) | 97.93 (95.37, 100.56) | 82.55 (80.91, 84.22) | 101.03 (100.23, 101.84) | 109.14 (107.99, 110.32) | 92.72 (91.69, 93.75) | 12.60 (12.34, 12.87) | 11.69 (11.33, 12.07) | 13.46 (13.09, 13.85) | 53.83 (53.08, 54.59) | 57.57 (56.65, 58.51) | 49.93 (49.28, 50.59) | 185.03 (182.27, 187.83) | 195.82 (191.65, 200.08) | 175.69 (172.04, 179.42) | 300.89 (283.68, 319.15) | 344.20 (323.49, 366.23) | 257.89 (244.1, 272.45) |
|  | 60~64  year | 90.77 (88.51, 93.09) | 98.45 (95.64, 101.35) | 83.24 (81.45, 85.08) | 120.61 (119.60, 121.64) | 129.03 (127.57, 130.51) | 112.32 (111.01, 113.65) | 12.80 (12.50, 13.12) | 11.80 (11.38, 12.24) | 13.73 (13.29, 14.18) | 51.65 (50.85, 52.46) | 54.43 (53.45, 55.42) | 48.92 (48.22, 49.62) | 192.55 (189.38, 195.77) | 204.21 (199.37, 209.17) | 183.01 (178.85, 187.26) | 233.58 (218.34, 249.88) | 273.58 (254.87, 293.65) | 194.73 (182.81, 207.43) |
|  | 65~69  year | 87.26 (84.52, 90.09) | 94.91 (91.49, 98.46) | 80.11 (77.93, 82.36) | 136.91 (135.43, 138.41) | 146.16 (144.04, 148.30) | 128.45 (126.51, 130.41) | 12.47 (12.08, 12.87) | 11.50 (10.96, 12.06) | 13.34 (12.79, 13.92) | 44.88 (43.99, 45.79) | 47.35 (46.26, 48.47) | 42.54 (41.77, 43.33) | 189.5 (185.58, 193.51) | 202.08 (196.00 , 208.35) | 179.75 (174.67, 184.98) | 174.49 (160.55, 189.65) | 206.86 (189.48, 225.82) | 144.3 (133.51, 155.95) |
|  | 70~74  year | 83.1 (79.99, 86.34) | 90.78 (86.86, 94.88) | 76.39 (73.92, 78.95) | 147.2 (145.33, 149.10) | 158.08 (155.39, 160.82) | 137.99 (135.54, 140.47) | 11.97 (11.51, 12.44) | 11.07 (10.43, 11.75) | 12.72 (12.08, 13.40) | 38.54 (37.59, 39.52) | 40.59 (39.42, 41.79) | 36.71 (35.88, 37.55) | 175.82 (171.51, 180.24) | 189.89 (182.99, 197.06) | 165.71 (160.25, 171.37) | 129.87 (117.34, 143.74) | 153.16 (137.41, 170.71) | 109.53 (99.82, 120.19) |
|  | 75~79  year | 77.52 (73.91, 81.30) | 86.07 (81.41, 91.00) | 70.69 (67.88, 73.62) | 147.66 (145.36, 150.00) | 162.40 (159.00 , 165.87) | 136.25 (133.31, 139.26) | 11.41 (10.86, 11.98) | 10.64 (9.86, 11.48) | 12.04 (11.29, 12.84) | 34.70 (33.59, 35.85) | 36.69 (35.31, 38.12) | 33.03 (32.07, 34.01) | 153.08 (148.46, 157.85) | 168.51 (160.73, 176.67) | 143.25 (137.57, 149.17) | 97.16 (85.61, 110.28) | 115.20 (100.28, 132.34) | 83.01 (74.15, 92.92) |
|  | 80~84  year | 68.30 (64.00, 72.89) | 78.43 (72.58, 84.75) | 61.20 (57.97, 64.62) | 135.24 (132.39, 138.14) | 157.74 (153.22, 162.39) | 120.32 (116.87, 123.87) | 10.7 (10.01, 11.43) | 10.1 (9.10, 11.21) | 11.18 (10.26, 12.19) | 31.71 (30.27, 33.23) | 33.48 (31.68, 35.39) | 30.3 (29.06, 31.59) | 127.32 (121.87, 133.01) | 143.93 (134.05, 154.54) | 118.54 (112.11, 125.34) | 68.58 (57.91, 81.22) | 83.57 (68.97, 101.26) | 58.74 (50.79, 67.95) |
|  | 85~89  year | 56.70 (51.10, 62.91) | 67.94 (59.64, 77.40) | 50.44 (46.43, 54.79) | 113.99 (110.02, 118.11) | 147.67 (140.27, 155.46) | 98.98 (94.54, 103.63) | 9.99 (9.04, 11.05) | 9.54 (8.12, 11.21) | 10.32 (9.07, 11.75) | 29.67 (27.47, 32.04) | 31.36 (28.61, 34.38) | 28.38 (26.49, 30.40) | 104.48 (97.39, 112.09) | 120.41 (106.55, 136.07) | 96.99 (88.97, 105.74) | 45.37 (35.28, 58.34) | 58.23 (43.12, 78.64) | 38.80 (31.55, 47.71) |
|  | 90~95  year | 46.49 (38.04, 56.82) | 57.2 (43.20, 75.73) | 42.26 (36.44, 49.00) | 89.98 (83.15, 97.37) | 133.37 (115.51, 153.98) | 83.30 (76.21, 91.04) | 9.47 (7.93, 11.31) | 8.90 (6.63, 11.93) | 9.73 (7.76, 12.20) | 28.60 (24.5, 33.37) | 30.32 (25.20, 36.49) | 27.28 (23.78, 31.29) | 89.17 (78.54, 101.24) | 103.39 (81.37, 131.38) | 83.17 (71.56, 96.66) | 30.33 (19.79, 46.51) | 42.36 (24.67, 72.73) | 25.75 (18.39, 36.06) |
|  | 95  plus | 41.35 (26.95, 63.44) | 53.16 (28.01, 100.92) | 37.51 (27.67, 50.83) | 76.42 (62.40, 93.59) | 109.12 (70.73, 168.33) | 74.23 (59.87, 92.04) | 9.47 (6.70, 13.38) | 8.86 (4.95, 15.88) | 9.6 (6.18, 14.91) | 29.01 (19.58, 42.99) | 30.74 (19.12, 49.45) | 27.27 (19.23, 38.67) | 83.32 (64.37, 107.84) | 85.7 (48.53, 151.34) | 76.27 (56.52, 102.91) | 22.19 (9.97, 49.40) | 33.49 (11.31, 99.19) | 19.09 (10.44, 34.92) |

Appendix table 7. Period RR of APC analysis across to different regions.

| Period  RR | Asia | | | | East Asia | | | Southeast Asia | | | South Asia | | | Central Asia | | | High-income | | |
| --- | --- | --- | --- | --- | --- | --- | --- | --- | --- | --- | --- | --- | --- | --- | --- | --- | --- | --- | --- |
|  | Period | Both | Male | Female | Both | Male | Female | Both | Male | Female | Both | Male | Female | Both | Male | Female | Both | Male | Female |
|  |  | Rate(95%CI) | Rate(95%CI) | Rate(95%CI) | Rate(95%CI) | Rate(95%CI) | Rate(95%CI) | Rate(95%CI) | Rate(95%CI) | Rate(95%CI) | Rate(95%CI) | Rate(95%CI) | Rate(95%CI) | Rate(95%CI) | Rate(95%CI) | Rate(95%CI) | Rate(95%CI) | Rate(95%CI) | Rate(95%CI) |
|  | 1900~1994 | 0.81 (0.79,0.84) | 0.80 0.78,0.83) | 0.82 (0.80,0.84) | 0.63 (0.62,0.63) | 0.63 (0.62,0.64) | 0.62 (0.61,0.63) | 0.73 (0.71,0.74) | 0.75 (0.73,0.77) | 0.71 (0.69,0.73) | 0.94 (0.92,0.96) | 0.94 (0.92,0.96) | 0.94 (0.93,0.96) | 1.01 (0.99,1.02) | 1.00 (0.97,1.02) | 1.01 (0.99,1.03) | 0.80 (0.74,0.87) | 0.79 (0.73,0.86) | 0.83 (0.77,0.89) |
|  | 1995~1999 | 0.92 (0.90,0.94) | 0.92 (0.89,0.94) | 0.92 (0.90,0.94) | 0.78 (0.77,0.78) | 0.78 (0.77,0.79) | 0.77 (0.76,0.78) | 0.85 (0.83,0.86) | 0.86 (0.84,0.88) | 0.84 (0.82,0.86) | 0.96 (0.95,0.98) | 0.96 (0.94,0.97) | 0.97 (0.96,0.98) | 1.00 (0.99,1.02) | 1.00 (0.98,1.02) | 1.01 (0.99,1.02) | 0.99 (0.94,1.04) | 0.98 (0.93,1.04) | 0.99 (0.95,1.05) |
|  | 2000~2004 | 1.00 (1.00,1.00) | 1.00 (1.00,1.00) | 1.00 (1.00,1.00) | 1.00 (1.00,1.00) | 1.00 (1.00,1.00) | 1.00 (1.00,1.00) | 1.00 (1.00,1.00) | 1.00 (1.00,1.00) | 1.00 (1.00,1.00) | 1.00 (1.00,1.00) | 1.00 (1.00,1.00) | 1.00 (1.00,1.00) | 1.00 (1.00,1.00) | 1.00 (1.00,1.00) | 1.00 (1.00,1.00) | 1.00 (1.00,1.00) | 1.00 (1.00,1.00) | 1.00 (1.00,1.00) |
|  | 2005~2009 | 1.01 (0.99,1.03) | 1.00 (0.98,1.03) | 1.01 (0.99,1.03) | 1.07 (1.06,1.08) | 1.06 (1.05,1.08) | 1.08 (1.07,1.09) | 1.02 (1.00,1.04) | 1.02 (1.00,1.05) | 1.02 (1.00,1.04) | 1.03 (1.02,1.05) | 1.04 (1.02,1.05) | 1.03 (1.02,1.04) | 1.01 (1.00,1.02) | 1.01 (0.99,1.03) | 1.01 (0.99,1.03) | 0.97 (0.92,1.03) | 0.97 (0.92,1.03) | 0.97 (0.92,1.02) |
|  | 2010~2014 | 1.01 (0.99,1.04) | 1.01 (0.98,1.04) | 1.02 (1.00,1.04) | 1.13 (1.12,1.15) | 1.12 (1.1,1.14) | 1.15 (1.14,1.17) | 1.04 (1.02,1.06) | 1.03 (1.00,1.06) | 1.04 (1.01,1.06) | 1.06 (1.04,1.08) | 1.06 (1.03,1.08) | 1.07 (1.05,1.08) | 1.03 (1.01,1.04) | 1.03 (1.01,1.06) | 1.02 (1.00,1.05) | 0.94 (0.87,1.02) | 0.94 (0.87,1.02) | 0.94 (0.88,1.01) |
|  | 2015~2019 | 0.99 (0.96,1.03) | 0.99 (0.95,1.03) | 1.01 (0.98,1.03) | 1.18 (1.17,1.20) | 1.17 (1.14,1.19) | 1.21 (1.18,1.23) | 1.04 (1.02,1.07) | 1.04 (1.00,1.08) | 1.05 (1.02,1.08) | 0.99 (0.97,1.01) | 0.97 (0.95,1.00) | 1.00 (0.98,1.03) | 1.03 (1.01,1.05) | 1.04 (1.01,1.07) | 1.03 (1.00,1.05) | 0.9 (0.81,0.99) | 0.88 (0.79,0.99) | 0.9 (0.82,0.99) |

Appendix table 8. Cohort RR of APC analysis across to different regions

| Cohort | Asia | | | | East Asia | | | Southeast Asia | | | South Asia | | | Central Asia | | | High-income | | |
| --- | --- | --- | --- | --- | --- | --- | --- | --- | --- | --- | --- | --- | --- | --- | --- | --- | --- | --- | --- |
|  | Year | Both | Male | Female | Both | Male | Female | Both | Male | Female | Both | Male | Female | Both | Male | Female | Both | Male | Female |
|  |  | Rate(95%CI) | Rate(95%CI) | Rate(95%CI) | Rate(95%CI) | Rate(95%CI) | Rate(95%CI) | Rate(95%CI) | Rate(95%CI) | Rate(95%CI) | Rate(95%CI) | Rate(95%CI) | Rate(95%CI) | Rate(95%CI) | Rate(95%CI) | Rate(95%CI) | Rate(95%CI) | Rate(95%CI) | Rate(95%CI) |
|  | 1895~1899 | 0.96 (0.20, 4.66) | 0.96 (0.09, 9.78) | 0.95 (0.31, 2.93) | 0.41 (0.18, 0.93) | 0.48 (0.09, 2.52) | 0.38 (0.16, 0.91) | 0.56 (0.17, 1.84) | 0.80 (0.10, 6.16) | 0.50 (0.12, 2.12) | 0.62 (0.12, 3.25) | 0.58 (0.08, 4.27) | 0.65 (0.15, 2.86) | 0.85 (0.48, 1.5) | 0.97 (0.34, 2.75) | 0.87 (0.44, 1.73) | 3.36 (0.16, 69.51) | 3.37 (0.06, 181.91) | 3.23 (0.32, 32.78) |
|  | 1900~1904 | 0.9 (0.49, 1.66) | 0.87 (0.37, 2.06) | 0.91 (0.58, 1.41) | 0.40 (0.30, 0.53) | 0.39 (0.23, 0.66) | 0.38 (0.28, 0.52) | 0.55 (0.33, 0.92) | 0.62 (0.27, 1.45) | 0.53 (0.28, 0.99) | 0.65 (0.36, 1.14) | 0.63 (0.32, 1.22) | 0.66 (0.39, 1.11) | 0.86 (0.67, 1.11) | 0.88 (0.56, 1.39) | 0.87 (0.64, 1.18) | 2.73 (0.85, 8.78) | 2.92 (0.66, 12.9) | 2.60 (1.04, 6.49) |
|  | 1905~1909 | 0.82 (0.62, 1.09) | 0.79 (0.54, 1.15) | 0.84 (0.68, 1.04) | 0.39 (0.35, 0.43) | 0.39 (0.33, 0.47) | 0.38 (0.33, 0.43) | 0.54 (0.42, 0.7) | 0.61 (0.41, 0.92) | 0.50 (0.35, 0.7) | 0.71 (0.56, 0.90) | 0.70 (0.53, 0.92) | 0.70 (0.56, 0.87) | 0.89 (0.78, 1.02) | 0.89 (0.70, 1.12) | 0.90 (0.77, 1.06) | 2.26 (1.26, 4.04) | 2.46 (1.21, 5.02) | 2.14 (1.34, 3.43) |
|  | 1910~1914 | 0.78 (0.67, 0.91) | 0.76 (0.63, 0.93) | 0.79 (0.69, 0.89) | 0.38 (0.36, 0.4) | 0.40 (0.36, 0.43) | 0.36 (0.34, 0.39) | 0.56 (0.48, 0.65) | 0.6 (0.47, 0.76) | 0.51 (0.41, 0.63) | 0.75 (0.67, 0.85) | 0.75 (0.65, 0.87) | 0.74 (0.66, 0.83) | 0.92 (0.85, 1.00) | 0.91 (0.79, 1.05) | 0.93 (0.84, 1.03) | 1.84 (1.31, 2.58) | 2.01 (1.35, 3.00) | 1.75 (1.33, 2.32) |
|  | 1915~1919 | 0.75 (0.68, 0.83) | 0.75 (0.66, 0.85) | 0.75 (0.69, 0.81) | 0.38 (0.37, 0.4) | 0.41 (0.39, 0.43) | 0.36 (0.35, 0.38) | 0.57 (0.52, 0.64) | 0.62 (0.53, 0.73) | 0.54 (0.47, 0.62) | 0.79 (0.73, 0.85) | 0.79 (0.72, 0.86) | 0.78 (0.73, 0.84) | 0.94 (0.89, 1.00) | 0.94 (0.85, 1.03) | 0.96 (0.90, 1.03) | 1.55 (1.23, 1.95) | 1.68 (1.28, 2.19) | 1.49 (1.22, 1.81) |
|  | 1920~1924 | 0.74 (0.69, 0.8) | 0.74 (0.68, 0.81) | 0.74 (0.69, 0.78) | 0.41 (0.40, 0.42) | 0.44 (0.42, 0.45) | 0.38 (0.37, 0.40) | 0.61 (0.57, 0.66) | 0.65 (0.58, 0.72) | 0.59 (0.53, 0.65) | 0.83 (0.79, 0.87) | 0.83 (0.78, 0.88) | 0.82 (0.78, 0.86) | 0.95 (0.91, 1.00) | 0.95 (0.88, 1.02) | 0.98 (0.92, 1.03) | 1.33 (1.11, 1.58) | 1.41 (1.15, 1.71) | 1.31 (1.13, 1.52) |
|  | 1925~1929 | 0.78 (0.74, 0.82) | 0.79 (0.74, 0.84) | 0.76 (0.73, 0.8) | 0.46 (0.45, 0.47) | 0.48 (0.47, 0.5) | 0.43 (0.42, 0.44) | 0.66 (0.62, 0.69) | 0.68 (0.63, 0.74) | 0.64 (0.59, 0.69) | 0.86 (0.83, 0.9) | 0.87 (0.83, 0.90) | 0.85 (0.83, 0.88) | 0.97 (0.94, 1.00) | 0.96 (0.92, 1.01) | 0.99 (0.95, 1.03) | 1.21 (1.06, 1.37) | 1.25 (1.09, 1.45) | 1.19 (1.06, 1.33) |
|  | 1930~1934 | 0.81 (0.78, 0.84) | 0.82 (0.79, 0.87) | 0.79 (0.76, 0.82) | 0.51 (0.51, 0.52) | 0.54 (0.53, 0.55) | 0.48 (0.47, 0.49) | 0.70 (0.67, 0.73) | 0.73 (0.68, 0.77) | 0.68 (0.65, 0.72) | 0.89 (0.87, 0.92) | 0.89 (0.87, 0.92) | 0.89 (0.86, 0.91) | 0.98 (0.96, 1.01) | 0.97 (0.93, 1.00) | 0.99 (0.96, 1.03) | 1.13 (1.02, 1.25) | 1.16 (1.04, 1.3) | 1.10 (1.00, 1.21) |
|  | 1935~1939 | 0.85 (0.82, 0.88) | 0.87 (0.83, 0.9) | 0.82 (0.79, 0.84) | 0.58 (0.57, 0.58) | 0.6 (0.59, 0.61) | 0.55 (0.54, 0.56) | 0.74 (0.72, 0.77) | 0.76 (0.72, 0.8) | 0.72 (0.69, 0.76) | 0.92 (0.90, 0.94) | 0.92 (0.90, 0.94) | 0.91 (0.89, 0.93) | 0.99 (0.96, 1.01) | 0.97 (0.94, 1.00) | 1.00 (0.97, 1.02) | 1.06 (0.98, 1.16) | 1.09 (1.00, 1.2) | 1.04 (0.96, 1.12) |
|  | 1940~1944 | 0.9 (0.87, 0.92) | 0.91 (0.88, 0.94) | 0.87 (0.85, 0.9) | 0.65 (0.64, 0.66) | 0.67 (0.66, 0.68) | 0.63 (0.62, 0.64) | 0.77 (0.75, 0.80) | 0.79 (0.75, 0.82) | 0.76 (0.73, 0.79) | 0.94 (0.92, 0.96) | 0.94 (0.92, 0.96) | 0.94 (0.92, 0.95) | 0.99 (0.98, 1.01) | 0.98 (0.96, 1.01) | 1.00 (0.98, 1.03) | 1.04 (0.97, 1.12) | 1.06 (0.98, 1.14) | 1.02 (0.95, 1.09) |
|  | 1945~1949 | 0.96 (0.94, 0.99) | 0.97 (0.94, 1.00) | 0.95 (0.93, 0.97) | 0.76 (0.75, 0.77) | 0.77 (0.76, 0.78) | 0.74 (0.73, 0.75) | 0.83 (0.81, 0.85) | 0.83 (0.80, 0.87) | 0.82 (0.79, 0.85) | 0.96 (0.95, 0.98) | 0.96 (0.94, 0.98) | 0.97 (0.95, 0.98) | 1.00 (0.98, 1.02) | 0.99 (0.96, 1.01) | 1.01 (0.98, 1.04) | 1.05 (0.98, 1.12) | 1.06 (0.99, 1.13) | 1.04 (0.98, 1.10) |
|  | 1950~1954 | 1.02 (1.00, 1.05) | 1.03 (1.00, 1.06) | 1.01 (0.99, 1.03) | 0.87 (0.87, 0.88) | 0.88 (0.87, 0.89) | 0.87 (0.85, 0.88) | 0.90 (0.88, 0.92) | 0.90 (0.87, 0.93) | 0.90 (0.87, 0.92) | 0.98 (0.97, 1.00) | 0.98 (0.96, 1.00) | 0.98 (0.97, 1) | 1.00 (0.99, 1.02) | 1.00 (0.97, 1.02) | 1.01 (0.98, 1.03) | 1.06 (1.00, 1.12) | 1.06 (1.00, 1.12) | 1.06 (1.00, 1.12) |
|  | 1955~1959 | 1.00 (1.00, 1.00) | 1.00 (1.00, 1.00) | 1.00 (1.00, 1.00) | 1.00 (1.00, 1.00) | 1.00 (1.00, 1.00) | 1.00 (1.00, 1.00) | 1.00 (1.00, 1.00) | 1.00 (1.00, 1.00) | 1.00 (1.00, 1.00) | 1.00 (1.00, 1.00) | 1.00 (1.00, 1.00) | 1.00 (1.00, 1.00) | 1.00 (1.00, 1.00) | 1.00 (1.00, 1.00) | 1.00 (1.00, 1.00) | 1.00 (1.00, 1.00) | 1.00 (1.00, 1.00) | 1.00 (1.00, 1.00) |
|  | 1960~1964 | 1.03 (1.01, 1.06) | 1.02 (1.00, 1.05) | 1.04 (1.02, 1.07) | 1.15 (1.14, 1.16) | 1.13 (1.12, 1.14) | 1.18 (1.17, 1.19) | 1.10 (1.08, 1.12) | 1.09 (1.06, 1.13) | 1.10 (1.07, 1.13) | 1.01 (1.00, 1.03) | 1.01 (1.00, 1.03) | 1.02 (1, 1.03) | 1.00 (0.99, 1.02) | 1.01 (0.99, 1.03) | 1.00 (0.98, 1.02) | 1.01 (0.96, 1.07) | 1.01 (0.96, 1.08) | 1.01 (0.95, 1.06) |
|  | 1965~1969 | 1.1 (1.07, 1.13) | 1.09 (1.06, 1.12) | 1.12 (1.09, 1.14) | 1.34 (1.33, 1.35) | 1.30 (1.29, 1.32) | 1.39 (1.38, 1.41) | 1.18 (1.16, 1.20) | 1.18 (1.14, 1.21) | 1.18 (1.15, 1.21) | 1.02 (1.01, 1.04) | 1.02 (1.00, 1.04) | 1.03 (1.01, 1.04) | 1.01 (0.99, 1.02) | 1.01 (0.99, 1.04) | 1.00 (0.98, 1.02) | 1.13 (1.07, 1.2) | 1.15 (1.08, 1.22) | 1.11 (1.05, 1.17) |
|  | 1970~1974 | 1.21 (1.18, 1.24) | 1.20 (1.16, 1.23) | 1.22 (1.20, 1.25) | 1.57 (1.55, 1.58) | 1.50 (1.48, 1.52) | 1.65 (1.63, 1.67) | 1.27 (1.25, 1.30) | 1.27 (1.23, 1.31) | 1.28 (1.24, 1.31) | 1.03 (1.01, 1.04) | 1.02 (1.00, 1.04) | 1.03 (1.02, 1.05) | 1.01 (0.99, 1.03) | 1.02 (1.00, 1.04) | 1.00 (0.98, 1.02) | 1.27 (1.2, 1.35) | 1.30 (1.22, 1.38) | 1.23 (1.16, 1.30) |
|  | 1975~1979 | 1.29 (1.26, 1.33) | 1.29 (1.25, 1.33) | 1.3 (1.27, 1.33) | 1.81 (1.79, 1.83) | 1.74 (1.71, 1.76) | 1.91 (1.88, 1.94) | 1.38 (1.35, 1.41) | 1.37 (1.32, 1.42) | 1.39 (1.35, 1.44) | 1.03 (1.01, 1.04) | 1.02 (1.00, 1.04) | 1.04 (1.02, 1.06) | 1.01 (1.00, 1.03) | 1.03 (1.00, 1.05) | 1.00 (0.98, 1.03) | 1.42 (1.33, 1.51) | 1.46 (1.37, 1.56) | 1.35 (1.27, 1.44) |
|  | 1980~1984 | 1.31 (1.27, 1.35) | 1.30 (1.25, 1.35) | 1.32 (1.28, 1.35) | 2.11 (2.09, 2.14) | 2.03 (2.00, 2.07) | 2.22 (2.18, 2.25) | 1.50 (1.47, 1.54) | 1.48 (1.42, 1.53) | 1.53 (1.48, 1.58) | 1.02 (1.00, 1.04) | 1.01 (0.98, 1.03) | 1.04 (1.02, 1.06) | 1.02 (1.00, 1.04) | 1.03 (1.00, 1.07) | 1.01 (0.98, 1.04) | 1.48 (1.38, 1.59) | 1.53 (1.42, 1.65) | 1.40 (1.30, 1.50) |
|  | 1985~1989 | 1.33 (1.28, 1.38) | 1.32 (1.27, 1.38) | 1.34 (1.30, 1.39) | 2.41 (2.38, 2.45) | 2.35 (2.31, 2.4) | 2.49 (2.44, 2.54) | 1.64 (1.59, 1.69) | 1.60 (1.54, 1.67) | 1.68 (1.62, 1.74) | 1.01 (0.99, 1.04) | 0.99 (0.97, 1.02) | 1.04 (1.01, 1.06) | 1.03 (1.00, 1.05) | 1.04 (1.01, 1.08) | 1.01 (0.98, 1.05) | 1.63 (1.49, 1.77) | 1.71 (1.56, 1.87) | 1.51 (1.39, 1.64) |
|  | 1990~1994 | 1.36 (1.30, 1.43) | 1.36 (1.29, 1.44) | 1.37 (1.32, 1.43) | 2.72 (2.67, 2.77) | 2.70 (2.63, 2.77) | 2.76 (2.69, 2.83) | 1.76 (1.70, 1.82) | 1.71 (1.63, 1.8) | 1.81 (1.73, 1.89) | 1.01 (0.98, 1.04) | 0.98 (0.95, 1.02) | 1.03 (1.01, 1.06) | 1.03 (1.00, 1.06) | 1.05 (1.01, 1.09) | 1.02 (0.98, 1.06) | 1.73 (1.55, 1.93) | 1.82 (1.62, 2.04) | 1.60 (1.44, 1.77) |
|  | 1995~1999 | 1.41 (1.32, 1.5) | 1.41 (1.31, 1.53) | 1.40 (1.32, 1.48) | 3.06 (2.98, 3.15) | 3.06 (2.95, 3.18) | 3.06 (2.95, 3.18) | 1.81 (1.73, 1.88) | 1.76 (1.66, 1.87) | 1.85 (1.75, 1.96) | 1.00 (0.96, 1.04) | 0.97 (0.93, 1.02) | 1.03 (1, 1.06) | 1.04 (0.99, 1.08) | 1.05 (0.99, 1.12) | 1.02 (0.97, 1.08) | 1.88 (1.61, 2.2) | 2.01 (1.71, 2.37) | 1.70 (1.46, 1.98) |
|  | 2000~2004 | 1.44 (1.30, 1.6) | 1.46 (1.29, 1.65) | 1.43 (1.30, 1.56) | 3.43 (3.28, 3.59) | 3.40 (3.20, 3.61) | 3.47 (3.26, 3.69) | 1.87 (1.76, 1.98) | 1.81 (1.66, 1.97) | 1.92 (1.77, 2.08) | 1.00 (0.95, 1.06) | 0.97 (0.91, 1.04) | 1.03 (0.99, 1.09) | 1.04 (0.97, 1.11) | 1.06 (0.96, 1.16) | 1.02 (0.94, 1.12) | 2.05 (1.55, 2.72) | 2.26 (1.69, 3.02) | 1.79 (1.37, 2.33) |
|  | 2005~2009 | 1.50 (1.23, 1.84) | 1.51 (1.20, 1.90) | 1.49 (1.25, 1.78) | 3.80 (3.49, 4.14) | 3.76 (3.36, 4.20) | 3.82 (3.37, 4.34) | 1.98 (1.80, 2.18) | 1.91 (1.68, 2.18) | 2.04 (1.77, 2.34) | 1.02 (0.93, 1.12) | 0.99 (0.89, 1.1) | 1.05 (0.96, 1.14) | 1.04 (0.92, 1.18) | 1.06 (0.90, 1.26) | 1.02 (0.85, 1.22) | 2.26 (1.19, 4.26) | 2.44 (1.26, 4.74) | 2.01 (1.10, 3.66) |
|  | 2010~2014 | 1.58 (1.00, 2.49) | 1.59 (0.95, 2.68) | 1.57 (1.05, 2.33) | 4.13 (3.39, 5.03) | 4.10 (3.18, 5.30) | 4.16 (3.12, 5.55) | 2.10 (1.74, 2.54) | 2.03 (1.57, 2.63) | 2.16 (1.63, 2.86) | 1.04 (0.85, 1.27) | 1.02 (0.81, 1.28) | 1.07 (0.89, 1.29) | 1.05 (0.80, 1.38) | 1.06 (0.72, 1.55) | 1.05 (0.70, 1.56) | 2.36 (0.41, 13.51) | 2.56 (0.40, 16.44) | 2.10 (0.42, 10.43) |
|  | 2015~2019 | 1.65 (0.21, 13.16) | 1.65 (0.15, 18.02) | 1.64 (0.27, 9.87) | 4.56 (1.90, 10.94) | 4.59 (1.45, 14.5) | 4.52 (1.3, 15.76) | 2.16 (0.96, 4.88) | 2.08 (0.71, 6.1) | 2.24 (0.65, 7.77) | 1.09 (0.46, 2.6) | 1.02 (0.37, 2.82) | 1.06 (0.47, 2.39) | 1.03 (0.29, 3.61) | 1.80 (0.35, 9.31) | 0.89 (0.10, 7.62) | 2.62 (0.00, 43266.86) | 2.86 (0.00, 63999.28) | 2.30 (0.00, 26029.88) |

Appendix table 9. Net drift and Local drift of APC analysis across to different regions.

| Local drift | Asia | | | | East Asia | | | Southeast Asia | | | South Asia | | | Central Asia | | | High-income | | |
| --- | --- | --- | --- | --- | --- | --- | --- | --- | --- | --- | --- | --- | --- | --- | --- | --- | --- | --- | --- |
|  | Age | Both | Male | Female | Both | Male | Female | Both | Male | Female | Both | Male | Female | Both | Male | Female | Both | Male | Female |
|  |  | Percent per year(95%CI) | Percent per year(95%CI) | Percent per year(95%CI) | Percent per year(95%CI) | Percent per year(95%CI) | Percent per year(95%CI) | Percent per year(95%CI) | Percent per year(95%CI) | Percent per year(95%CI) | Percent per year(95%CI) | Percent per year(95%CI) | Percent per year(95%CI) | Percent per year(95%CI) | Percent per year(95%CI) | Percent per year(95%CI) | Percent per year(95%CI) | Percent per year(95%CI) | Percent per year(95%CI) |
|  | 0~4 | 0.77 (-5.11, 7.03) | 0.78 (-5.96, 8.02) | 0.74 (-4.37, 6.12) | 2.07 (-0.49, 4.69) | 2.1 (-1.25, 5.56) | 2.01 (-1.62, 5.78) | 0.88 (-1.47, 3.3) | 0.83 (-2.28, 4.04) | 0.91 (-2.66, 4.62) | 0.3 (-2.19, 2.86) | 0.20 (-2.71, 3.2) | 0.14 (-2.21, 2.54) | 0.01 (-3.57, 3.73) | 1.57 (-3.17, 6.53) | -0.35 (-6.35, 6.03) | 1.63 (-23.21, 34.52) | 1.77 (-23.79, 35.9) | 1.49 (-22.46, 32.83) |
|  | 5~9 | 0.67 (-0.71, 2.07) | 0.73 (-0.85, 2.33) | 0.6 (-0.61, 1.82) | 2.20 (1.59, 2.81) | 2.24 (1.45, 3.03) | 2.12 (1.24, 3.01) | 0.93 (0.34, 1.52) | 0.88 (0.09, 1.68) | 0.95 (0.09, 1.82) | 0.1 (-0.51, 0.71) | 0.09 (-0.61, 0.79) | 0.12 (-0.44, 0.69) | 0.08 (-0.74, 0.92) | 0.07 (-1.08, 1.23) | 0.10 (-1.10, 1.32) | 1.58 (-3.56, 6.99) | 1.74 (-3.72, 7.52) | 1.38 (-3.35, 6.34) |
|  | 10~14 | 0.55 (-0.07, 1.18) | 0.61 (-0.09, 1.33) | 0.48 (-0.07, 1.03) | 2.38 (2.11, 2.65) | 2.49 (2.14, 2.84) | 2.21 (1.82, 2.61) | 1.03 (0.73, 1.33) | 0.97 (0.56, 1.38) | 1.07 (0.64, 1.51) | -0.03 (-0.33, 0.26) | -0.09 (-0.43, 0.25) | 0.03 (-0.25, 0.3) | 0.08 (-0.3, 0.46) | 0.1 (-0.42, 0.63) | 0.05 (-0.50, 0.60) | 1.67 (-0.27, 3.64) | 1.88 (-0.14, 3.95) | 1.38 (-0.44, 3.24) |
|  | 15~19 | 0.46 (0.12, 0.79) | 0.52 (0.13, 0.91) | 0.38 (0.10, 0.67) | 2.56 (2.42, 2.71) | 2.74 (2.55, 2.94) | 2.35 (2.15, 2.55) | 1.23 (1.04, 1.41) | 1.15 (0.88, 1.41) | 1.30 (1.05, 1.56) | -0.11 (-0.28, 0.07) | -0.18 (-0.39, 0.02) | -0.03 (-0.18, 0.13) | 0.09 (-0.11, 0.3) | 0.12 (-0.17, 0.42) | 0.08 (-0.2, 0.36) | 1.52 (0.65, 2.40) | 1.76 (0.85, 2.68) | 1.18 (0.36, 2.01) |
|  | 20~24 | 0.53 (0.32, 0.75) | 0.58 (0.33, 0.82) | 0.48 (0.30, 0.66) | 2.73 (2.64, 2.82) | 2.92 (2.80, 3.05) | 2.50 (2.37, 2.62) | 1.48 (1.34, 1.61) | 1.37 (1.18, 1.57) | 1.58 (1.39, 1.76) | -0.11 (-0.23, 0.01) | -0.2 (-0.34, -0.05) | -0.03 (-0.13, 0.08) | 0.1 (-0.02, 0.23) | 0.14 (-0.05, 0.32) | 0.09 (-0.09, 0.26) | 1.53 (1.03, 2.04) | 1.70 (1.18, 2.23) | 1.26 (0.79, 1.74) |
|  | 25~29 | 0.79 (0.63, 0.94) | 0.81 (0.64, 0.99) | 0.76 (0.62, 0.89) | 2.89 (2.82, 2.95) | 2.99 (2.90, 3.07) | 2.78 (2.69, 2.86) | 1.64 (1.53, 1.74) | 1.52 (1.36, 1.68) | 1.75 (1.60, 1.89) | -0.06 (-0.16, 0.03) | -0.15 (-0.26, -0.04) | 0.02 (-0.06, 0.1) | 0.10 (0.01, 0.19) | 0.14 (0.01, 0.28) | 0.07 (-0.05, 0.2) | 1.68 (1.33, 2.04) | 1.84 (1.47, 2.21) | 1.42 (1.08, 1.76) |
|  | 30~34 | 1.06 (0.94, 1.19) | 1.08 (0.94, 1.23) | 1.04 (0.93, 1.15) | 3.02 (2.96, 3.07) | 2.98 (2.91, 3.05) | 3.06 (2.99, 3.13) | 1.62 (1.52, 1.71) | 1.53 (1.39, 1.68) | 1.71 (1.58, 1.83) | 0.00 (-0.07, 0.08) | -0.07 (-0.16, 0.02) | 0.07 (0.01, 0.14) | 0.08 (0.01, 0.16) | 0.14 (0.02, 0.25) | 0.05 (-0.06, 0.15) | 1.90 (1.61, 2.19) | 2.08 (1.78, 2.37) | 1.62 (1.34, 1.89) |
|  | 35~39 | 1.21 (1.10, 1.32) | 1.21 (1.07, 1.34) | 1.22 (1.13, 1.32) | 3.05 (3.00, 3.09) | 2.88 (2.82, 2.94) | 3.25 (3.18, 3.31) | 1.61 (1.52, 1.70) | 1.55 (1.42, 1.69) | 1.67 (1.55, 1.79) | 0.09 (0.02, 0.15) | 0.02 (-0.06, 0.1) | 0.15 (0.09, 0.21) | 0.07 (0.00, 0.15) | 0.13 (0.03, 0.24) | 0.03 (-0.07, 0.13) | 1.78 (1.52, 2.04) | 1.94 (1.67, 2.20) | 1.53 (1.29, 1.77) |
|  | 40~44 | 1.04 (0.93, 1.15) | 1.00 (0.87, 1.12) | 1.11 (1.01, 1.20) | 2.99 (2.94, 3.03) | 2.76 (2.70, 2.82) | 3.27 (3.21, 3.33) | 1.70 (1.60, 1.79) | 1.66 (1.52, 1.80) | 1.73 (1.61, 1.86) | 0.18 (0.12, 0.24) | 0.13 (0.06, 0.21) | 0.23 (0.17, 0.29) | 0.05 (-0.02, 0.12) | 0.12 (0.02, 0.22) | -0.01 (-0.11, 0.09) | 1.32 (1.08, 1.56) | 1.45 (1.20, 1.69) | 1.12 (0.89, 1.34) |
|  | 45~49 | 0.81 (0.70, 0.91) | 0.72 (0.60, 0.85) | 0.93 (0.83, 1.02) | 2.93 (2.88, 2.97) | 2.68 (2.63, 2.74) | 3.24 (3.18, 3.30) | 1.77 (1.67, 1.87) | 1.73 (1.58, 1.88) | 1.80 (1.67, 1.94) | 0.25 (0.19, 0.32) | 0.24 (0.17, 0.32) | 0.28 (0.22, 0.34) | 0.04 (-0.03, 0.12) | 0.13 (0.02, 0.23) | -0.04 (-0.14, 0.07) | 0.67 (0.43, 0.92) | 0.73 (0.47, 0.99) | 0.57 (0.33, 0.80) |
|  | 50~54 | 0.70 (0.58, 0.82) | 0.59 (0.46, 0.72) | 0.86 (0.76, 0.96) | 2.90 (2.85, 2.94) | 2.68 (2.62, 2.74) | 3.21 (3.15, 3.28) | 1.77 (1.66, 1.88) | 1.69 (1.52, 1.85) | 1.84 (1.69, 1.99) | 0.34 (0.27, 0.41) | 0.33 (0.25, 0.41) | 0.36 (0.30, 0.43) | 0.04 (-0.03, 0.12) | 0.12 (0.02, 0.23) | -0.03 (-0.13, 0.07) | 0.14 (-0.12, 0.42) | 0.12 (-0.16, 0.4) | 0.15 (-0.10, 0.41) |
|  | 55~59 | 0.80 (0.67, 0.93) | 0.67 (0.53, 0.82) | 0.97 (0.86, 1.08) | 2.83 (2.78, 2.87) | 2.62 (2.56, 2.69) | 3.12 (3.05, 3.19) | 1.62 (1.50, 1.75) | 1.50 (1.31, 1.69) | 1.74 (1.56, 1.91) | 0.40 (0.33, 0.48) | 0.39 (0.31, 0.48) | 0.43 (0.36, 0.50) | 0.07 (-0.01, 0.14) | 0.13 (0.02, 0.24) | 0.01 (-0.1, 0.12) | -0.21 (-0.51, 0.1) | -0.31 (-0.63, 0.01) | -0.11 (-0.4, 0.17) |
|  | 60~64 | 0.97 (0.83, 1.12) | 0.88 (0.72, 1.05) | 1.10 (0.97, 1.23) | 2.74 (2.68, 2.79) | 2.55 (2.48, 2.62) | 3.00 (2.92, 3.07) | 1.39 (1.24, 1.54) | 1.25 (1.02, 1.47) | 1.52 (1.31, 1.72) | 0.46 (0.37, 0.55) | 0.45 (0.34, 0.55) | 0.49 (0.41, 0.57) | 0.08 (0.00, 0.17) | 0.14 (0.01, 0.26) | 0.04 (-0.08, 0.16) | -0.35 (-0.7, 0.01) | -0.48 (-0.86, -0.11) | -0.23 (-0.56, 0.1) |
|  | 65~69 | 1.11 (0.93, 1.29) | 1.08 (0.87, 1.28) | 1.17 (1.01, 1.32) | 2.62 (2.56, 2.69) | 2.42 (2.33, 2.50) | 2.87 (2.78, 2.96) | 1.21 (1.02, 1.39) | 1.06 (0.77, 1.34) | 1.33 (1.08, 1.57) | 0.51 (0.39, 0.63) | 0.50 (0.36, 0.63) | 0.56 (0.45, 0.67) | 0.12 (0.01, 0.22) | 0.14 (-0.02, 0.3) | 0.08 (-0.06, 0.22) | -0.51 (-0.94, -0.08) | -0.66 (-1.13, -0.19) | -0.44 (-0.83, -0.05) |
|  | 70~74 | 1.02 (0.78, 1.25) | 1.05 (0.77, 1.33) | 0.98 (0.78, 1.17) | 2.46 (2.38, 2.54) | 2.25 (2.14, 2.36) | 2.65 (2.54, 2.77) | 1.17 (0.93, 1.42) | 1.00 (0.63, 1.38) | 1.30 (0.97, 1.62) | 0.59 (0.42, 0.75) | 0.58 (0.39, 0.77) | 0.63 (0.48, 0.78) | 0.17 (0.03, 0.31) | 0.15 (-0.08, 0.39) | 0.12 (-0.06, 0.31) | -0.95 (-1.52, -0.39) | -1.13 (-1.76, -0.5) | -0.95 (-1.44, -0.46) |
|  | 75~79 | 0.76 (0.44, 1.09) | 0.84 (0.45, 1.24) | 0.65 (0.38, 0.91) | 2.18 (2.06, 2.29) | 2.00 (1.84, 2.15) | 2.28 (2.13, 2.43) | 1.22 (0.87, 1.56) | 1.01 (0.49, 1.55) | 1.36 (0.91, 1.82) | 0.68 (0.44, 0.92) | 0.69 (0.41, 0.98) | 0.71 (0.49, 0.93) | 0.22 (0.03, 0.41) | 0.18 (-0.13, 0.5) | 0.16 (-0.07, 0.4) | -1.54 (-2.28, -0.79) | -1.77 (-2.62, -0.92) | -1.51 (-2.14, -0.87) |
|  | 80~84 | 0.4 (-0.09, 0.89) | 0.55 (-0.07, 1.17) | 0.23 (-0.16, 0.62) | 1.78 (1.60, 1.96) | 1.71 (1.45, 1.98) | 1.75 (1.52, 1.99) | 1.21 (0.70, 1.72) | 1.01 (0.22, 1.80) | 1.45 (0.78, 2.12) | 0.80 (0.41, 1.18) | 0.81 (0.36, 1.26) | 0.82 (0.47, 1.17) | 0.29 (0.02, 0.55) | 0.26 (-0.2, 0.72) | 0.25 (-0.08, 0.58) | -2.14 (-3.19, -1.08) | -2.41 (-3.64, -1.17) | -2.05 (-2.92, -1.17) |
|  | 85~89 | -0.06 (-0.92, 0.81) | 0.16 (-0.97, 1.31) | -0.24 (-0.9, 0.42) | 1.17 (0.82, 1.51) | 1.30 (0.76, 1.84) | 1.01 (0.59, 1.43) | 1.08 (0.27, 1.91) | 0.74 (-0.52, 2.02) | 1.35 (0.27, 2.44) | 0.94 (0.21, 1.67) | 0.96 (0.13, 1.81) | 0.95 (0.28, 1.62) | 0.39 (-0.03, 0.81) | 0.34 (-0.4, 1.09) | 0.39 (-0.13, 0.90) | -2.76 (-4.5, -0.99) | -3.01 (-5.12, -0.86) | -2.61 (-4.02, -1.18) |
|  | 90~94 | -0.63 (-2.43, 1.2) | -0.41 (-2.94, 2.19) | -0.74 (-2.06, 0.6) | 0.52 (-0.33, 1.37) | 0.85 (-0.73, 2.45) | 0.33 (-0.6, 1.27) | 0.73 (-0.81, 2.3) | 0.37 (-2.17, 2.97) | 0.87 (-1.06, 2.85) | 1.15 (-0.56, 2.88) | 1.25 (-0.74, 3.28) | 1.06 (-0.5, 2.65) | 0.48 (-0.3, 1.27) | 0.38 (-1.04, 1.82) | 0.52 (-0.43, 1.47) | -3.29 (-6.65, 0.19) | -3.42 (-7.65, 1.00) | -3.13 (-5.78, -0.41) |
|  | 95~99 | -1.09 (-5.6, 3.63) | -0.99 (-7.55, 6.04) | -1.09 (-4.34, 2.26) | -0.08 (-2.44, 2.34) | -0.14 (-4.89, 4.84) | -0.08 (-2.61, 2.52) | 0.32 (-3.16, 3.94) | -0.63 (-6.49, 5.6) | 0.55 (-3.74, 5.04) | 1.24 (-3.58, 6.3) | 1.45 (-4.3, 7.54) | 0.99 (-3.29, 5.47) | 0.5 (-1.23, 2.25) | 0.06 (-3.09, 3.32) | 0.53 (-1.55, 2.65) | -3.68 (-11.92, 5.33) | -3.5 (-14.2, 8.53) | -3.59 (-9.97, 3.24) |
| Net drift |  | 0.74 (0.55, 0.94) | 0.75 (0.50, 0.99) | 0.76 (0.60, 0.91) | 2.54 (2.46, 2.63) | 2.45 (2.31, 2.60) | 2.65 (2.54, 2.76) | 1.41 (1.28, 1.54) | 1.27 (1.07, 1.48) | 1.51 (1.34, 1.68) | 0.33 (0.19, 0.47) | 0.30 (0.14, 0.47) | 0.36 (0.24, 0.49) | 0.12 (0.02, 0.23) | 0.18 (0.02, 0.33) | 0.08 (-0.08, 0.23) | 0.22 (-0.43, 0.87) | 0.22 (-0.47, 0.92) | 0.14 (-0.47, 0.74) |

Appendix table 10. Changes in incidence number according to population-level determinants and causes from 1990 to 2019 across to location

| location | Overall difference ^a^ | Change due to population-level determinants (% contribution to the total changes) | | |
| --- | --- | --- | --- | --- |
|  |  | Aging ^b^ | Population ^c^ | Epidemiological change ^d^ |
| Asia | 80,792.98 | 20,648.38(25.56%) | 35,496.48 (43.94%) | 24,648.12(30.51%) |
| East Asia | 34,751.47 | 6,213.86(17.88%) | 6,048.70 (17.41%) | 22,488.91(64.71%) |
| South Asia | 21,265.33 | 6,052.42 (28.46%) | 13,668.19(64.27%) | 1,544.72(7.26%) |
| Southeast Asia | 3,098.03 | 583.50 (18.83%) | 1,16790 (37.7%) | 1,346.63 (43.47%) |
| Central Asia | 2,947.19 | 829.90 (28.16%) | 1,447.02 (49.1%) | 670.27 (22.74%) |
| a Change in incidence number between 2019 and 1990. | | | | |
| b Change in incidence number due to change in the age structure. | | | | |
| c Change in incidence number due to change in population number. | | | | |
| d Change in incidence number due to epidemiologic changes. Epidemiologic changes refer to the incidence number change when age structure and population hold constant. | | | | |
| Appendix table 11. Changes in deaths number according to population-level determinants and causes from 1990 to 2019 across to location   \| location \| Overall difference ^a^ \| Change due to population-level determinants (% contribution to the total changes) \| \| \| \| --- \| --- \| --- \| --- \| --- \| \|  \|  \| Aging ^b^ \| Population ^c^ \| Epidemiological change ^d^ \| \| Asia \| 1,927.80 \| 7,145.34 (370.65%) \| 4,934.26 (255.95%) \| -10,151.80 (-526.6%) \| \| East Asia \| -751.48 \| 4,334.41 (6199.552%) \| 1,113.67 (576.78%) \| -6,199.55 (-824.98%) \| \| South Asia \| 1,838.30 \| 1,904.42 (103.6%) \| 2,427.58 (132.06%) \| -2,493.71 (-135.65%) \| \| Southeast Asia \| 268.00 \| 624.49 (233.02%) \| 522.74 (195.05%) \| -879.23 (-328.07%) \| \| Central Asia \| 33.80 \| 23.46 (69.41%) \| 70.06 (207.27%) \| -59.72 (-176.69%) \| \| a Change in deaths number between 2019 and 1990. \| \| \| \| \| \| b Change in deaths number due to change in the age structure. \| \| \| \| \| \| c Change in deaths number due to change in population number. \| \| \| \| \| \| d Change in deaths number due to epidemiologic changes. Epidemiologic changes refer to the deaths number change when age structure and population hold constant. \| \| \| \| \| | | | | |

Appendix table 12. Frontier analysis of 52 Asia countries.

| Location | 1990 | | | | 2019 | | | |
| --- | --- | --- | --- | --- | --- | --- | --- | --- |
|  | ASDR | SDI | frontier | eff_diff | ASDR | SDI | frontier | eff_diff |
| American Samoa | 44.49 | 0.606 | 4.48 | 40.02 | 15.67 | 0.712 | 3.56 | 12.12 |
| Armenia | 19.99 | 0.536 | 4.62 | 15.37 | 20.50 | 0.689 | 3.58 | 16.92 |
| Azerbaijan | 17.65 | 0.576 | 4.67 | 12.98 | 16.20 | 0.683 | 3.44 | 12.76 |
| Bangladesh | 31.40 | 0.267 | 21.05 | 10.35 | 18.10 | 0.483 | 5.03 | 13.07 |
| Bhutan | 21.23 | 0.228 | 21.23 | 0.00 | 15.82 | 0.455 | 6.09 | 9.73 |
| Brunei Darussalam | 36.51 | 0.676 | 3.65 | 32.86 | 32.76 | 0.823 | 3.46 | 29.30 |
| Cambodia | 14.51 | 0.266 | 14.51 | 0.00 | 8.98 | 0.469 | 5.92 | 3.07 |
| China | 24.47 | 0.433 | 6.33 | 18.14 | 13.10 | 0.686 | 3.46 | 9.64 |
| Cook Islands | 50.74 | 0.625 | 4.38 | 46.36 | 24.94 | 0.764 | 3.46 | 21.48 |
| Democratic People's Republic of Korea | 17.24 | 0.431 | 6.16 | 11.08 | 11.10 | 0.558 | 4.62 | 6.48 |
| Fiji | 10.67 | 0.527 | 4.59 | 6.08 | 8.17 | 0.664 | 3.67 | 4.50 |
| Georgia | 20.37 | 0.654 | 3.88 | 16.49 | 20.25 | 0.702 | 3.46 | 16.79 |
| Guam | 9.01 | 0.693 | 3.59 | 5.42 | 5.39 | 0.813 | 3.58 | 1.81 |
| India | 20.11 | 0.327 | 7.66 | 12.45 | 13.04 | 0.566 | 4.59 | 8.45 |
| Indonesia | 19.48 | 0.452 | 5.77 | 13.71 | 11.66 | 0.66 | 3.70 | 7.96 |
| Japan | 27.14 | 0.791 | 3.59 | 23.55 | 46.58 | 0.87 | 3.55 | 43.03 |
| Kazakhstan | 30.23 | 0.602 | 4.60 | 25.63 | 26.97 | 0.723 | 3.55 | 23.41 |
| Kiribati | 53.42 | 0.425 | 6.29 | 47.13 | 31.83 | 0.527 | 4.55 | 27.27 |
| Kyrgyzstan | 28.00 | 0.532 | 4.58 | 23.42 | 17.37 | 0.596 | 4.51 | 12.86 |
| Lao People's Democratic Republic | 13.04 | 0.268 | 13.04 | 0.00 | 6.97 | 0.49 | 4.86 | 2.10 |
| Malaysia | 5.01 | 0.542 | 4.74 | 0.28 | 4.92 | 0.737 | 3.60 | 1.32 |
| Maldives | 13.21 | 0.303 | 7.96 | 5.25 | 7.20 | 0.562 | 4.59 | 2.60 |
| Marshall Islands | 38.63 | 0.398 | 6.52 | 32.11 | 24.94 | 0.544 | 4.57 | 20.37 |
| Mauritius | 5.30 | 0.527 | 4.63 | 0.67 | 7.70 | 0.705 | 3.55 | 4.15 |
| Micronesia (Federated States of) | 40.59 | 0.447 | 5.72 | 34.87 | 23.88 | 0.58 | 4.60 | 19.28 |
| Mongolia | 38.19 | 0.465 | 5.39 | 32.80 | 24.75 | 0.606 | 4.50 | 20.25 |
| Myanmar | 7.97 | 0.284 | 7.97 | 0.00 | 4.34 | 0.521 | 4.34 | 0.00 |
| Nauru | 42.03 | 0.499 | 4.68 | 37.35 | 27.18 | 0.618 | 4.46 | 22.72 |
| Nepal | 28.52 | 0.198 | 28.52 | 0.00 | 15.65 | 0.422 | 6.34 | 9.31 |
| Niue | 29.80 | 0.566 | 4.61 | 25.19 | 19.00 | 0.711 | 3.46 | 15.54 |
| Northern Mariana Islands | 34.73 | 0.692 | 3.45 | 31.28 | 11.56 | 0.771 | 3.46 | 8.10 |
| Pakistan | 21.52 | 0.247 | 21.42 | 0.11 | 17.80 | 0.449 | 6.07 | 11.73 |
| Palau | 26.21 | 0.621 | 4.37 | 21.85 | 19.34 | 0.738 | 3.59 | 15.75 |
| Papua New Guinea | 19.34 | 0.292 | 7.87 | 11.47 | 14.68 | 0.394 | 6.50 | 8.18 |
| Philippines | 8.94 | 0.497 | 4.97 | 3.97 | 4.87 | 0.623 | 4.28 | 0.59 |
| Republic of Korea | 31.72 | 0.686 | 3.55 | 28.16 | 15.00 | 0.878 | 3.55 | 11.45 |
| Samoa | 27.95 | 0.531 | 4.63 | 23.32 | 17.97 | 0.641 | 4.00 | 13.97 |
| Seychelles | 8.38 | 0.567 | 4.68 | 3.69 | 6.28 | 0.724 | 3.55 | 2.73 |
| Singapore | 12.19 | 0.688 | 3.44 | 8.74 | 8.16 | 0.861 | 3.44 | 4.72 |
| Solomon Islands | 24.39 | 0.279 | 15.05 | 9.34 | 17.28 | 0.407 | 6.41 | 10.86 |
| Sri Lanka | 5.73 | 0.504 | 4.66 | 1.07 | 3.77 | 0.69 | 3.44 | 0.33 |
| Taiwan (Province of China) | 17.35 | 0.667 | 3.66 | 13.69 | 12.40 | 0.868 | 3.55 | 8.85 |
| Tajikistan | 30.29 | 0.468 | 5.32 | 24.97 | 20.60 | 0.539 | 4.63 | 15.98 |
| Thailand | 6.06 | 0.508 | 4.64 | 1.42 | 3.64 | 0.687 | 3.45 | 0.19 |
| Timor-Leste | 11.64 | 0.274 | 11.64 | 0.00 | 9.14 | 0.514 | 4.75 | 4.39 |
| Tokelau | 25.84 | 0.427 | 6.35 | 19.50 | 16.11 | 0.626 | 4.27 | 11.85 |
| Tonga | 31.30 | 0.51 | 4.70 | 26.60 | 21.58 | 0.636 | 3.92 | 17.66 |
| Turkmenistan | 24.59 | 0.548 | 4.43 | 20.16 | 22.90 | 0.67 | 3.76 | 19.14 |
| Tuvalu | 33.30 | 0.426 | 6.28 | 27.02 | 21.17 | 0.589 | 4.66 | 16.52 |
| Uzbekistan | 23.48 | 0.49 | 5.00 | 18.48 | 21.90 | 0.631 | 4.17 | 17.73 |
| Vanuatu | 34.45 | 0.361 | 6.92 | 27.53 | 25.36 | 0.485 | 5.07 | 20.29 |
| Viet Nam | 13.87 | 0.39 | 6.45 | 7.41 | 7.60 | 0.617 | 4.46 | 3.13 |

Appendix table 13. Incidence and Deaths projections of inflammatory bowel disease across different regions from 2020 to 2044.

| Year | Asia | | | | East Asia | | | | Southeast Asia | | | | South Asia | | | | Central Asia | | | | High-income | | | |
| --- | --- | --- | --- | --- | --- | --- | --- | --- | --- | --- | --- | --- | --- | --- | --- | --- | --- | --- | --- | --- | --- | --- | --- | --- |
|  | Incidence Number | Incidence Rate (per 100000) | Death number | Death Rate (per 100000) | Incidence Number | Incidence Rate (per 100000) | Death number | Death Rate (per 100000) | Incidence Number | Incidence Rate (per 100000) | Death number | Death Rate (per 100000) | Incidence Number | Incidence Rate (per 100000) | Death number | Death Rate (per 100000) | Incidence Number | Incidence Rate (per 100000) | Death number | Death Rate (per 100000) | Incidence Number | Incidence Rate (per 100000) | Death number | Death Rate (per 100000) |
| 2020 | 149,904.500 | 2.94 | 14,063.660 | 0.31 | 53,578.390 | 2.98 | 5,100.211 | 0.29 | 5,073.456 | 0.7 | 1,517.004 | 0.27 | 39,884.170 | 2.24 | 5,646.848 | 0.42 | 6,587.888 | 6.88 | 254.631 | 0.31 | 27,167.250 | 15.09 | 664.712 | 0.13 |
| 2021 | 152,038.200 | 2.94 | 14,250.110 | 0.31 | 54,731.690 | 3.03 | 5,096.556 | 0.28 | 5,187.500 | 0.71 | 1,541.279 | 0.27 | 40,100.940 | 2.2 | 5,740.305 | 0.42 | 6,718.189 | 6.9 | 257.978 | 0.31 | 27,120.850 | 15.17 | 676.088 | 0.13 |
| 2022 | 154,139.800 | 2.95 | 14,402.010 | 0.3 | 55,864.220 | 3.09 | 5,065.252 | 0.26 | 5,301.649 | 0.72 | 1,563.941 | 0.27 | 40,294.630 | 2.17 | 5,827.904 | 0.41 | 6,847.85 | 6.92 | 261.096 | 0.31 | 27,069.650 | 15.24 | 684.596 | 0.13 |
| 2023 | 155,987.900 | 2.95 | 14,694.570 | 0.3 | 56,682.040 | 3.13 | 5,136.778 | 0.26 | 5,403.459 | 0.72 | 1,598.274 | 0.26 | 40,614.880 | 2.14 | 5,943.722 | 0.41 | 6,964.849 | 6.94 | 264.891 | 0.3 | 26,989.140 | 15.3 | 700.064 | 0.13 |
| 2024 | 157,801.900 | 2.95 | 15,017.630 | 0.29 | 57,470.110 | 3.16 | 5,225.372 | 0.25 | 5,505.085 | 0.73 | 1,636.211 | 0.26 | 40,921.370 | 2.12 | 6,068.860 | 0.41 | 7,081.237 | 6.95 | 268.870 | 0.3 | 26,899.980 | 15.37 | 716.434 | 0.13 |
| 2025 | 159,576.500 | 2.95 | 15,349.710 | 0.29 | 58,233.080 | 3.2 | 5,316.023 | 0.25 | 5,606.288 | 0.73 | 1,675.922 | 0.26 | 41,208.950 | 2.09 | 6,197.357 | 0.4 | 7,197.475 | 6.96 | 272.9545 | 0.3 | 26,805.160 | 15.43 | 732.713 | 0.13 |
| 2026 | 161,312.500 | 2.96 | 15,676.260 | 0.29 | 58,977.080 | 3.24 | 5,398.962 | 0.24 | 5,706.867 | 0.74 | 1,716.025 | 0.25 | 41,474.280 | 2.06 | 6,325.284 | 0.4 | 7,313.662 | 6.98 | 277.076 | 0.3 | 26,707.240 | 15.49 | 748.191 | 0.13 |
| 2027 | 163,017.700 | 2.96 | 15,987.920 | 0.29 | 59,710.830 | 3.28 | 5,468.032 | 0.24 | 5,806.742 | 0.74 | 1,755.521 | 0.25 | 41,715.950 | 2.04 | 6,450.178 | 0.4 | 7,429.539 | 6.99 | 281.173 | 0.3 | 26,608.080 | 15.55 | 762.335 | 0.13 |
| 2028 | 164,478.100 | 2.96 | 16,438.250 | 0.28 | 60,094.070 | 3.3 | 5,625.015 | 0.23 | 5,892.357 | 0.75 | 1,806.720 | 0.25 | 42,085.410 | 2.02 | 6,604.113 | 0.39 | 7,533.265 | 6.99 | 286.308 | 0.29 | 26,480.300 | 15.59 | 781.708 | 0.13 |
| 2029 | 1652,898.600 | 2.96 | 16,921.090 | 0.28 | 60,451.060 | 3.32 | 5,797.239 | 0.23 | 5,977.004 | 0.75 | 1,861.912 | 0.25 | 42,440.410 | 2 | 6,767.214 | 0.39 | 7,636.935 | 6.99 | 291.781 | 0.29 | 26,346.250 | 15.64 | 801.941 | 0.13 |
| 2030 | 167,274.800 | 2.95 | 17,419.480 | 0.28 | 60,793.860 | 3.34 | 5,977.406 | 0.23 | 6,060.476 | 0.75 | 1,919.295 | 0.25 | 42,774.190 | 1.98 | 6,935.042 | 0.39 | 7,740.586 | 6.99 | 297.390 | 0.29 | 26,208.000 | 15.68 | 822.172 | 0.13 |
| 2031 | 168,607.800 | 2.95 | 17,920.520 | 0.28 | 61,135.620 | 3.36 | 6,159.501 | 0.23 | 6,142.325 | 0.76 | 1,977.306 | 0.24 | 43,082.860 | 1.96 | 7,105.243 | 0.39 | 7,843.899 | 7 | 302.962 | 0.29 | 26,066.760 | 15.72 | 841.657 | 0.13 |
| 2032 | 169,908.100 | 2.95 | 18,413.400 | 0.28 | 61,499.060 | 3.39 | 6,338.665 | 0.23 | 6,222.373 | 0.76 | 2,034.650 | 0.24 | 43,362.080 | 1.94 | 7,275.178 | 0.39 | 7,945.968 | 7 | 308.325 | 0.29 | 25,921.860 | 15.77 | 859.735 | 0.13 |
| 2033 | 170,944.400 | 2.95 | 19,054.200 | 0.28 | 61,467.600 | 3.39 | 6,617.483 | 0.23 | 6,287.37 | 0.76 | 2,104.805 | 0.24 | 43,772.340 | 1.93 | 7,475.225 | 0.39 | 8,035.985 | 6.99 | 314.762 | 0.29 | 25,753.720 | 15.8 | 883.016 | 0.13 |
| 2034 | 171,926.700 | 2.94 | 19,729.570 | 0.28 | 61407.730 | 3.39 | 6,921.263 | 0.23 | 6,351.595 | 0.76 | 2,178.682 | 0.24 | 44,165.590 | 1.91 | 7,683.546 | 0.39 | 8,126.072 | 6.99 | 321.351 | 0.29 | 25,584.400 | 15.82 | 906.756 | 0.13 |
| 2035 | 172,853.100 | 2.94 | 20,423.150 | 0.29 | 61,333.300 | 3.4 | 7,239.139 | 0.23 | 6,414.577 | 0.76 | 2,254.463 | 0.24 | 44,536.180 | 1.9 | 7,897.426 | 0.39 | 8,216.021 | 6.98 | 327.985 | 0.29 | 25,413.880 | 15.85 | 930.356 | 0.14 |
| 2036 | 173,724.200 | 2.93 | 21,120.490 | 0.29 | 61257.820 | 3.4 | 7,560.238 | 0.23 | 6,475.95 | 0.77 | 2,330.538 | 0.24 | 44,879.810 | 1.89 | 8,114.7,12 | 0.39 | 8,305.078 | 6.97 | 334.565 | 0.28 | 25,242.030 | 15.88 | 953.291 | 0.14 |
| 2037 | 174,549.900 | 2.93 | 21,809.590 | 0.29 | 61,202.200 | 3.4 | 7,874.462 | 0.23 | 6,535.24 | 0.77 | 2,405.442 | 0.24 | 45,193.230 | 1.88 | 8,333.767 | 0.39 | 8,392.455 | 6.96 | 341.007 | 0.28 | 25,065.980 | 15.91 | 975.100 | 0.14 |
| 2038 | 175,283.300 | 2.92 | 22,536.460 | 0.29 | 61048.440 | 3.41 | 8,205.402 | 0.23 | 6,590.145 | 0.77 | 2,482.283 | 0.24 | 45,519.610 | 1.86 | 8,560.930 | 0.39 | 8,476.903 | 6.96 | 347.625 | 0.28 | 24,883.800 | 15.93 | 998.154 | 0.14 |
| 2039 | 175,953.7 | 2.92 | 23,292.15 | 0.29 | 60,870.210 | 3.41 | 8,554.751 | 0.24 | 6,643.865 | 0.77 | 2,561.397 | 0.24 | 45,824.280 | 1.85 | 8,796.964 | 0.39 | 8,561.068 | 6.95 | 354.411 | 0.28 | 24,702.790 | 15.96 | 1,021.521 | 0.14 |
| 2040 | 176,559.200 | 2.92 | 24,062.940 | 0.29 | 60,678.480 | 3.41 | 8,911.872 | 0.24 | 6,695.949 | 0.77 | 2,641.276 | 0.24 | 46,101.740 | 1.84 | 9,039.721 | 0.39 | 8,644.528 | 6.94 | 361.206 | 0.28 | 24,523.360 | 15.98 | 1,044.628 | 0.14 |
| 2041 | 177,100.900 | 2.91 | 24,836.510 | 0.29 | 60,484.180 | 3.42 | 9,266.356 | 0.24 | 6,746.151 | 0.77 | 2,720.603 | 0.24 | 46,347.500 | 1.83 | 9,287.243 | 0.39 | 8,726.374 | 6.93 | 367.865 | 0.28 | 24,345.610 | 16 | 1,066.939 | 0.14 |
| 2042 | 177,588.600 | 2.91 | 25,602.610 | 0.3 | 60,305.000 | 3.42 | 9,608.597 | 0.24 | 6,794.247 | 0.77 | 2,798.224 | 0.24 | 46,558.340 | 1.82 | 9,538.008 | 0.39 | 8,805.684 | 6.93 | 374.271 | 0.28 | 24,167.470 | 16.03 | 1,087.988 | 0.15 |
| 2043 | 177,990.400 | 2.9 | 26,380.790 | 0.3 | 60,084.830 | 3.42 | 9,950.216 | 0.24 | 6,840.738 | 0.78 | 2,875.903 | 0.24 | 46,742.650 | 1.81 | 9,795.806 | 0.39 | 8,883.793 | 6.92 | 380.615 | 0.28 | 23,989.570 | 16.05 | 1,108.228 | 0.15 |
| 2044 | 178,323.900 | 2.9 | 27,187.150 | 0.3 | 59,835.540 | 3.43 | 10,303.31 | 0.24 | 6,885.736 | 0.78 | 2,954.954 | 0.24 | 46,902.000 | 1.79 | 10,063.40 | 0.39 | 8,960.455 | 6.91 | 387.040 | 0.28 | 23,810.380 | 16.07 | 1,128.208 | 0.15 |
